# Efficacy and Safety of Ravulizumab in IgA Nephropathy: A Phase 2 Randomized Double-Blind Placebo-Controlled Trial

**DOI:** 10.1681/ASN.0000000534

**Published:** 2024-10-25

**Authors:** Richard Lafayette, James Tumlin, Roberta Fenoglio, Jessica Kaufeld, Miguel Ángel Pérez Valdivia, Mai-Szu Wu, Shih-Han Susan Huang, Eric Alamartine, Sung Gyun Kim, Min Yee, Andreas Kateifides, Kara Rice, Katherine Garlo, Jonathan Barratt

**Affiliations:** 1 Stanford Glomerular Disease Center, Stanford University Medical Center, Stanford, California; 2 Department of Nephrology, Emory University School of Medicine, Atlanta, Georgia; 3 University Center of Excellence on Nephrological, Rheumatological and Rare Diseases including Nephrology and Dialysis Unit and Center of Immuno-Rheumatology and Rare Diseases, Coordinating Center of the Inter-regional Network for Rare Diseases of Piedmont and Aosta Valley (North-West Italy), San Giovanni Bosco Hub Hospital, ASL Città di Torino, Department of Clinical and Biological Sciences of the University of Turin, Turin, Italy; 4 Hannover Medical School, Department of Nephrology and Hypertension, Hannover, Germany; 5 Servicio de Nefrología, UGC Urología-Nefrología, Hospital Virgen del Rocío, Seville, Spain; 6 Division of Nephrology, Shuang-Ho Hospital, Taipei Medical University, Taipei, Taiwan; 7 Kidney Clinical Research Unit, London Health Sciences Center, East London, Ontario, Canada; 8 Service de Néphrologie, Hôpital Nord CHU Saint-Étienne, Saint-Étienne, France; 9 Department of Internal Medicine, Hallym University Sacred Heart Hospital, Anyang, Korea; 10 Alexion, AstraZeneca Rare Disease, Boston, Massachusetts; 11 Department of Cardiovascular Sciences, University of Leicester, Leicester, United Kingdom

**Keywords:** chronic GN, CKD, clinical trial, complement, GN, IgA nephropathy, randomized controlled trials, glomerular diseases

## Abstract

**Key Points:**

- This phase 2, double-blind, randomized controlled trial evaluated the complement C5 inhibitor, ravulizumab, in adults with IgA nephropathy.
- A 30.1% (90% confidence interval, 13.7% to 43.5%) relative reduction in proteinuria for ravulizumab versus placebo was observed at approximately 6 months.
- Treatment with ravulizumab was well tolerated.

**Background:**

The complement system plays a central role in the pathogenesis of IgA nephropathy. We present findings from a phase 2 trial of ravulizumab, a complement C5 inhibitor.

**Methods:**

The Study of Ravulizumab in Proliferative Lupus Nephritis or IgA Nephropathy (NCT04564339) was a randomized, double-blind, placebo-controlled trial of ravulizumab in addition to standard of care. Adults with IgA nephropathy, proteinuria ≥1 g/d, and eGFR ≥30 ml/min per 1.73 m^2^, and on stable renin-angiotensin blockade were randomized 2:1 to ravulizumab (intravenous every 8 weeks) or placebo for 26 weeks. From week 26–50, all participants received open-label ravulizumab. The primary end point was percentage change in proteinuria from baseline to week 26. Secondary end points included change in proteinuria at week 50 and eGFR. Safety, pharmacokinetics, and pharmacodynamics were evaluated.

**Results:**

Forty-three patients were randomized to ravulizumab and 23 to placebo. At week 26, a statistically significant reduction in proteinuria was observed with ravulizumab versus placebo: −41.9% (95% confidence interval [CI], −50.2% to −32.0%) change in urine protein with ravulizumab and −16.8% (95% CI, −31.8% to 1.6%) change with placebo (30.1% treatment effect; *P* = 0.005). At week 50, there was a −44.8% (95% CI, −55.1% to −32.1%) change from baseline in urine protein with ravulizumab, and in patients who crossed over from placebo to ravulizumab at week 26, the change from baseline (week 0) to week 50 was −45.1% (−58.0% to −28.4%). The least squares mean change in eGFR from baseline to week 26 with ravulizumab was 0.2 (95% CI, −2.3 to 2.7) ml/min per 1.73 m^2^ and with placebo was −4.5 (−7.9 to −1.1) ml/min per 1.73 m^2^. From baseline to week 50, the least squares mean change in eGFR with ravulizumab was −3.9 (95% CI, −6.4 to−1.3) ml/min per 1.73 m^2^, and in patients who crossed over from placebo to ravulizumab at week 26, it was −6.3 (−9.7 to −2.9) ml/min per 1.73 m^2^. Ravulizumab was well tolerated, with an adverse event profile similar to that for placebo.

**Conclusions:**

An early, sustained, and clinically meaningful reduction in proteinuria and trend toward stabilization of eGFR were observed with ravulizumab versus placebo. A phase 3 trial (NCT06291376) is enrolling.

**Clinical Trial registry name and registration number:**

Study of Ravulizumab in Proliferative Lupus Nephritis or IgA Nephropathy, NCT04564339.

**Podcast:**

This article contains a podcast at https://dts.podtrac.com/redirect.mp3/www.asn-online.org/media/podcast/JASN/2024_10_26_KTS_October2024.mp3

## Visual Abstract

**Figure:**
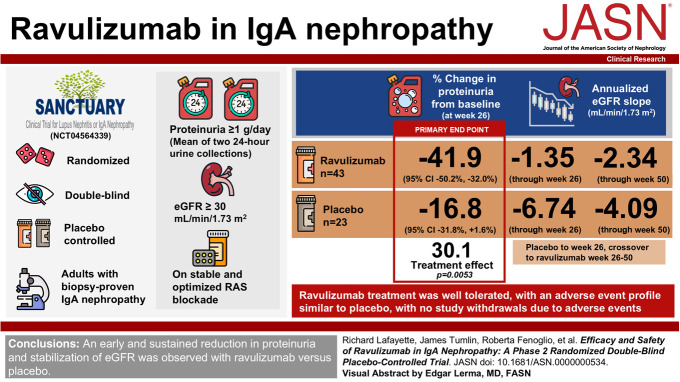

## Introduction

IgA nephropathy is a progressive GN and globally is an important cause of CKD. While the disease is heterogeneous with a variable clinical course,^1^ it affects many individuals during early to middle adulthood, and in a large registry study, approximately half reached kidney failure within 10 years of diagnosis at a median age of 55 years.^2^ Several other observational studies have demonstrated the substantial burden of IgA nephropathy,^3–7^ and timely treatment to modify the disease pathogenesis and prevent progression of CKD is crucial.

Treatment options for IgA nephropathy have largely been limited to supportive care, primarily renin-angiotensin system (RAS) blockade and immunosuppression with corticosteroids. Proteinuria over time, and particularly at ≥1.0 g/d, is predictive of a decline in kidney function and kidney failure.^8,9^ Large cohort studies have recently found that even those with proteinuria between 0.5 and 1.0 g/d have high rates of kidney failure.^8,9^ Conversely, a reduction in proteinuria is linked to improved kidney outcomes^10^ and is an accepted surrogate end point for use in registrational clinical trials in IgA nephropathy, with confirmatory data.^11^

In December 2023, targeted-release budesonide (Nefecon) became the first drug fully approved by the US Food and Drug Administration (FDA) for IgA nephropathy.^12^ Nefecon acts on the gut-associated lymphoid tissue in the distal ileum to block the production of galactose-deficient IgA1 (Gd-IgA1) by B cells.^13^ In addition, sparsentan, a dual endothelin and angiotensin receptor antagonist, received accelerated approval by the FDA for use in patients at high risk of disease progression.^14^ Iptacopan, a factor B inhibitor acting on the alternative complement pathway, has also received FDA accelerated approval for use in patients at risk of rapid disease progression.^15^ Sodium-glucose cotransporter-2 (SGLT2) inhibitors are increasingly used in IgA nephropathy,^16^ as secondary analyses of large phase 3 CKD trials have found proteinuria reduction and less kidney disease progression in patients with advanced IgA nephropathy.^17,18^

The pathogenesis of IgA nephropathy is believed to be a multihit process, beginning with an increase in levels of specific O-glycoforms of IgA1, commonly termed Gd-IgA1. This Gd-IgA1 leads to the production of IgG, IgA, or IgM autoantibodies that recognize the hinge region O-glycans of IgA1 and bind to form immune complexes. The immune complexes deposit in the glomerulus, leading to mesangial proliferation, production of extracellular matrix, release of proinflammatory cytokines, and complement activation. Complement activation may also occur in response to immune complexes in circulation.^19–21^

In IgA nephropathy, initial complement activation is believed to occur primarily through the alternative and lectin pathways and less commonly through the classical pathway.^19^ Regardless of the initiating pathway, complement component 3 (C3) convertase is activated, generating C3a and C3b, the latter of which combines with C4b2a or C3bBb to form C5 convertase, engaging the terminal pathway.^19,22^ C5 convertase cleaves C5 into C5a (a highly potent anaphylatoxin) and C5b, which leads to formation of C5b–9 (membrane attack complex). Insertion of C5b–9 in glomerular mesangial cells leads to the production of inflammatory cytokines, extracellular matrix, and fibrotic factors, and drives cell apoptosis. The glomerular basement membrane is damaged, and the inflammation and cellular damage manifests as hematuria and proteinuria.^19,22–25^ Complement activation is also implicated in the development of tubular atrophy and interstitial inflammation and fibrosis.^26,27^ Several studies have provided evidence of the involvement of the terminal pathway of complement in the progression of IgA nephropathy.^26–29^

Ravulizumab, a humanized mAb, is a second-generation C5 inhibitor engineered from eculizumab; it is designed to achieve immediate, complete, and sustained inhibition of C5 at an extended weight-based dosing regimen. By binding to C5, ravulizumab blocks its activation by C5 convertase and prevents the formation of C5a and C5b, therefore inhibiting the terminal complement pathway and leaving the proximal pathways intact. By targeting the final, common pathway in the complement cascade, inhibition of complement is achieved regardless of the initiating pathway (alternative, lectin, or classical). Ravulizumab is already approved to treat complement-mediated diseases (atypical hemolytic uremic syndrome, paroxysmal nocturnal hemoglobinuria, generalized myasthenia gravis, and neuromyelitis optica spectrum disorder).^30^ In this study, we report results from the primary evaluation and extension periods through week 50 of the Study of Ravulizumab in Proliferative Lupus Nephritis or IgA Nephropathy, evaluating the efficacy and safety of ravulizumab in adults with IgA nephropathy.

## Methods

### Study Design

The Study of Ravulizumab in Proliferative Lupus Nephritis or IgA Nephropathy (NCT04564339, date of first registration September 2020) was a phase 2, multicenter, randomized, double-blind, placebo-controlled trial of ravulizumab (intravenous weight-based dosing every 8 weeks [Q8W]) in addition to concomitant standard-of-care therapy. The trial included two disease cohorts, IgA nephropathy and lupus nephritis, the latter of which is not presented here. Participants underwent an eligibility screening period of up to 6 weeks and were randomized 2:1 to ravulizumab or placebo using interactive response technology. Randomization was stratified by mean proteinuria (1.0–2.0 versus >2.0 g/d) from the mean of two 24-hour urine collections during the screening period.

In the initial evaluation period, patients received ravulizumab or placebo for 26 weeks. From weeks 26 to 50 (the extension period), all participants received open-label ravulizumab (Supplemental Figure 1). Participants received a weight-based loading dose on day 1, weight-based maintenance dose on day 15, followed by weight-based dosing every 8 weeks (Supplemental Table 1). In the extension period, participants who received placebo in the initial evaluation period received a blinded weight-based loading dose of ravulizumab, and those who had received ravulizumab in the initial evaluation period received a blinded bridging dose of 900 mg ravulizumab. Starting at week 28, all participants received open-label ravulizumab every 8 weeks. The bridging dose was designed to maintain the blind-to-treatment allocation during the primary evaluation period. Throughout the trial, participants continued concomitant therapy consistent with the standard of care (RAS inhibition).

Participants, investigative site personnel, and employees or designees from the study sponsor directly associated with the conduct of the study were blinded to treatment assignments. Placebo had identical appearance to ravulizumab, and identical study drug kits and labels were used. The study sponsor was unblinded at week 26 to conduct the primary analysis.

The study was conducted in accordance with consensus ethical principles from international guidelines, including the Declaration of Helsinki and the Council for International Organizations of Medical Sciences International Ethical Guidelines and applicable International Council for Harmonization of Technical Requirements for Pharmaceuticals for Human Use Good Clinical Practice Guidelines. This clinical trial was evaluated and approved by the institutional review board or independent ethics committee at each participating center.

### Participants

Eligibility criteria included age 18–75 years, biopsy-proven IgA nephropathy at any time before or during screening, mean proteinuria ≥1.0 g/d on two complete and valid 24-hour urine collections during the screening period, and adherence to a stable and optimal dose of RAS inhibition (to include the maximum allowed or tolerated dose of angiotensin-converting enzyme inhibitor and/or angiotensin II receptor blocker for ≥3 months before screening with no projected change during the study period). If patients were on SGLT2 inhibitors, they must have been on a stable dose for ≥3 months with no planned change in dose during the study. All participants were required to be vaccinated against meningococcal infection within 3 years before randomization. Participants who did not meet this requirement were vaccinated against *Neisseria meningitides* before randomization and received prophylactic antibiotics for at least 2 weeks after vaccination if randomization occurred <2 weeks after initial vaccination.

Key exclusion criteria included eGFR <30 ml/min per 1.73 m^2^, secondary causes of IgA nephropathy, significant concomitant kidney disease, rapidly progressive GN (as measured by eGFR loss ≥30% over a period of 3 months before or during screening), BP ≥140/90 mm Hg, treatment with prednisone or a prednisone equivalent >20 mg/d for at least 2 weeks, or any other systemic immunosuppression ≤6 months before screening. Full eligibility criteria are given in the Supplemental Material. Informed consent was gathered from all study participants before performing any study-related procedures.

### Outcomes

The primary efficacy end point was percentage change in proteinuria from baseline to week 26, on the basis of absolute urine protein (g/d). This was derived from the mean of two 24-hour urine collections during the screening period and within 2 weeks of the week 26 visit. Proteinuria was also assessed by urine protein-creatinine ratio (UPCR) in g/g.

Secondary end points included percentage change in proteinuria from baseline to week 50, change from baseline in eGFR (calculated using the Chronic Kidney Disease Epidemiology Collaboration formula), percentage of participants with >30% and >50% reduction in proteinuria, percentage of participants meeting the criteria for partial remission (defined as mean proteinuria <1.0 g/24 hours on the basis of two valid 24-hour urine collections), and changes in serum C3 and C4. Annualized eGFR slope was an exploratory end point. Other prespecified analyses included proteinuria change by morning spot urine samples, percentage change in proteinuria by subgroups of baseline demographic and clinical characteristics, and percentage change in 24-hour urine albumin-creatinine ratio. A *post hoc* hematuria analysis evaluated the percentage of patients who had <5 red blood cells per high-power field on urine sediment from spot samples reported by a central pathology laboratory. All efficacy end points were based on the full analysis set (all randomized participants who received ≥1 dose of the study intervention); participants were analyzed as randomized. Safety outcomes (adverse events [AEs] and serious AEs), immunogenicity (anti-drug antibodies), and pharmacokinetics/pharmacodynamics were evaluated. Further details are provided in the Supplemental Material.

### Statistical Analyses

A 20%–30% reduction in the geometric mean of proteinuria has been proposed as likely to be necessary to ensure a significant treatment effect on the clinical outcome in early CKD.^31^ Sample size calculations were based on a one-sided, two-sample *t* test of log-transformed proteinuria values. A common SD for change in log-transformed proteinuria values was assumed to be 0.60.^32^ Under these assumptions and an anticipated 10% dropout rate, a sample size of 60 participants (40 randomized to ravulizumab, 20 randomized to placebo) was calculated to provide approximately 80% power to detect at least a 30% relative treatment effect with a one-sided significance level of 0.05.

To reduce skewness, natural logarithm was used to transform proteinuria values before analysis. An analysis of covariance model was used for the primary end point and included change from baseline in log-transformed proteinuria as the response variable and was adjusted for baseline log proteinuria and the randomization stratification factor. Data collected on or after treatment discontinuation were assumed to be missing at random and imputed using multiple imputation (number of imputations=1000). The treatment effect was evaluated using the least squares mean difference between treatment groups. The point estimate and two-sided 90% confidence interval (CI) for the mean difference of log-transformed proteinuria was back-transformed (by exponentiation) to obtain the geometric mean ratio and corresponding two-sided 90% CI. The values were then expressed as adjusted percentage change (*e.g*., [geometric mean ratio−1]×100%) from baseline in proteinuria at week 26.

Secondary end point analyses were based on the full analysis set and were descriptive, and no adjustment for multiplicity was performed. A mixed-effect model for repeated measures was used for percentage change in proteinuria at week 50 and for change in eGFR. Because patients on placebo switched to ravulizumab at week 26, the percentage change in proteinuria at week 50 was summarized descriptively without a formal treatment comparison. The slope of eGFR was computed using a mixed-effect model with random patient effects for intercepts and slopes. Further details are provided in the Supplemental Material.

## Results

### Patient Disposition, Demographics, and Baseline Characteristics

**Figure 1:**
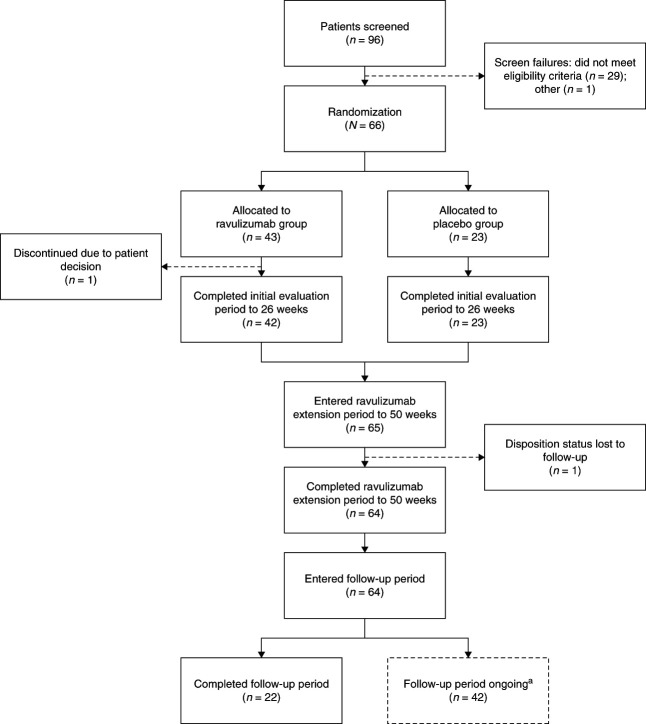
**Participant disposition.** ^a^As of February 2024.

Ninety-six patients were screened, 66 of whom met eligibility criteria and were randomized between May 2021 and November 2022. Patients were enrolled and treated at 38 sites across 11 countries in North America, Europe, and Asia Pacific. Forty-three participants were randomized to ravulizumab and 23 to placebo. One participant in the ravulizumab arm did not complete the initial evaluation period because of withdrawal of consent, and another participant in the ravulizumab arm was lost to follow-up during the extension period (Figure 1).

Overall, 46% of participants were femal, the mean (SD) age was 40 (11) years, 71% were White, and 21% were Asian (Table 1). Mean baseline eGFR was higher in the ravulizumab group. Nine participants (21%) in the ravulizumab arm and four (17%) in the placebo arm used an SGLT2 inhibitor during the initial evaluation period and extension period; one additional participant (on ravulizumab) started SGLT2 inhibition during the extension period. Baseline biopsy characteristics (local pathology) are shown in Supplemental Tables 2 and 3.

**Table 1:** Baseline demographics and clinical characteristics of study participants

Variable	Ravulizumab (*n*=43)	Placebo (*n*=23)	Total (*N*=66)
**Sex, *n* (%)**			
Male	21 (49)	15 (65)	36 (55)
Female	22 (51)	8 (35)	30 (46)
**Race, *n* (%)^a^**			
Asian	7 (16)	7 (30)	14 (21)
Not reported or other	5 (12)	0 (0)	5 (8)
White	31 (72)	16 (70)	47 (71)
**Geographical region, *n* (%)**			
Asia Pacific	5 (12)	6 (26)	11 (17)
Europe	30 (70)	12 (52)	42 (64)
North America	8 (19)	5 (22)	13 (20)
**Age at screening, yr**			
Mean (SD)	41 (10)	39 (12)	40 (11)
Median (Q1–Q3)	40 (33–49)	36 (32–44)	38 (33–48)
Min, max	21, 59	22, 69	21, 69
**Baseline weight, kg**			
Mean (SD)	77 (17)	78 (18)	77 (18)
Median (Q1–Q3)	72 (65–88)	82 (62–88)	74 (65–88)
Min, max	48, 129	46, 110	46, 129
**Duration of disease, yr^b^**			
Mean (SD)	5.0 (5.6)	3.9 (5.2)	4.6 (5.4)
Median (Q1–Q3)	2.6 (0.8–8.7)	1.4 (0.4–5.3)	2.5 (0.6–5.7)
Min, max	0.2, 24.9	0.1, 18.9	0.1, 24.9
**Baseline eGFR, ml/min per 1.73 m** ^**2**^			
Mean (SD)	76 (30)	70 (30)	74 (30)
Median (Q1–Q3)	74 (48–105)	63 (47–100)	65 (48–102)
eGFR 30 to ≤60, *n* (%)	19 (44)	11 (48)	30 (46)
eGFR >60 to ≤90, *n* (%)	7 (16)	6 (26)	13 (20)
eGFR >90, *n* (%)	17 (40)	6 (26)	23 (35)
**Baseline 24-h urine protein, g/d**			
Mean (SD)	2.6 (1.3)	3.0 (1.8)	2.8 (1.5)
Median (Q1–Q3)	2.3 (1.6–3.5)	2.7 (1.7–4.1)	2.4 (1.6–3.6)
1 to ≤2 g/d, *n* (%)	22 (51)	11 (48)	33 (50)
>2 g/d, *n* (%)	21 (49)	12 (52)	33 (50)
**Baseline 24-h UPCR, g/g**			
Mean (SD)	1.7 (0.9)	1.8 (1.0)	1.7 (0.9)
Median (Q1–Q3)	1.4 (1.1–2.3)	1.5 (1.0–2.7)	1.4 (1.1–2.3)
**Background therapies, *n* (%)**			
ACE inhibitors	20 (47)	7 (30)	27 (41)
ARBs	25 (58)	16 (70)	41 (62)
SGLT2 inhibitors	9 (21)	4 (17)	13 (20)
Hypertension, *n* (%)	20 (47)	13 (57)	33 (50)
Type 2 diabetes mellitus, *n* (%)	1 (2)	1 (4)	2 (3)

ACE, angiotensin-converting enzyme; ARBs, angiotensin II receptor blockers; IQR, interquartile range; SGLT2, sodium-glucose cotransporter-2; UPCR, urine protein-creatinine ratio.

a All other categories (American Indian or Alaska Native, Black, Native Hawaiian or Other Pacific Islander, unknown, and missing) had *n*=0. In the ravulizumab group, there were four patients with race not reported and one patient with race reported as other.

b Disease duration is defined as the duration from the date of disease diagnosis to the first dose of the study drug.

### Primary Outcome: Proteinuria Reduction at Week 26

**Figure 2:**
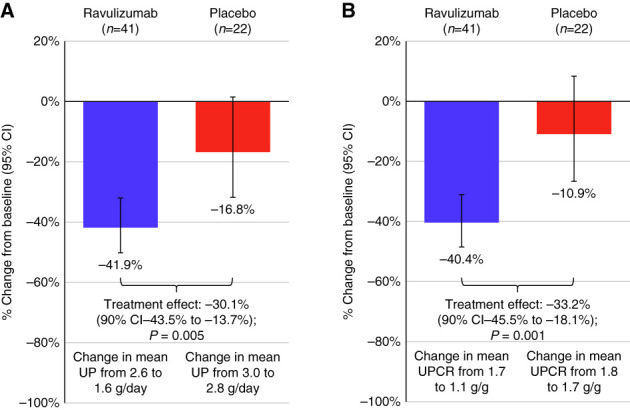
**At week 26, a statistically significant reduction in proteinuria was observed with ravulizumab versus placebo**. (A) Percentage change in proteinuria from baseline to week 26 measured by 24-hour urine protein (g/d; primary outcome). (B) Percentage change in proteinuria from baseline to week 26 measured by 24-hour UPCR (g/g; sensitivity analysis). CI, confidence interval; UP, urine protein; UPCR, urine protein-creatinine ratio.

At week 26, a statistically significant reduction in proteinuria from baseline was observed with ravulizumab versus placebo (Figure 2). For the primary end point, a −41.9% (95% CI, −50.2% to −32.0%) change from baseline in 24-hour urine protein was observed with ravulizumab and a −16.8% change (95% CI, −31.8% to 1.6%) was seen with placebo. This yielded a −30.1% relative treatment effect (90% CI, −43.5% to −13.7%; *P* = 0.005). In the ravulizumab arm, the mean (SD) 24-hour urine protein was 2.6 (1.3) g/d at baseline and 1.6 (0.9) g/d at week 26; in the placebo arm, the mean (SD) 24-hour urine protein was 3.0 (1.8) g/d at baseline and 2.8 (2.5) g/d at week 26. The 24-hour UPCR was also assessed: Patients in the ravulizumab arm experienced a −40.4% (95% CI, −48.5% to −31.1%) change from baseline, and patients in the placebo arm had a −10.9% (95% CI, −26.6% to 8.3%) change from baseline, for a treatment effect of −33.2% (90% CI, −45.5% to −18.1%; *P* = 0.001). In the ravulizumab arm, the mean (SD) 24-hour UPCR was 1.7 (0.9) g/g at baseline and 1.1 (0.6) g/g at week 26; in the placebo arm, the mean (SD) 24-hour UPCR was 1.8 (1.0) g/g at baseline and 1.7 (1.2) g/g at week 26.

### Secondary and Exploratory End Points

**Figure 3:**
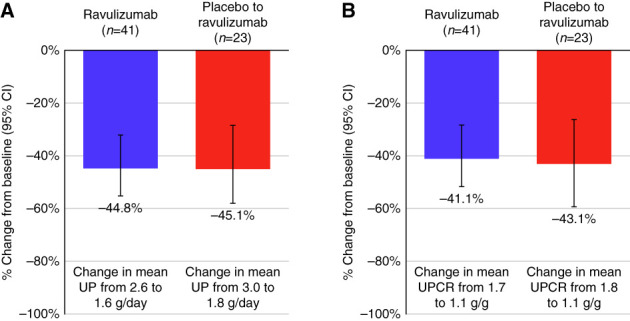
**Proteinuria reduction was sustained to week 50, and patients who crossed over from placebo to ravulizumab at week 26 had a similar decrease in proteinuria by week 50.** (A) Percentage change in proteinuria from baseline to week 50 in patients treated with ravulizumab and patients who crossed over from placebo to ravulizumab at week 26, measured by 24-hour UP (g/d; secondary end point). (B) Percentage change in proteinuria from baseline to week 50 in patients treated with ravulizumab and patients who crossed over from placebo to ravulizumab at week 26, measured by 24-hour UPCR (g/g; sensitivity analysis).

**Figure 4:**
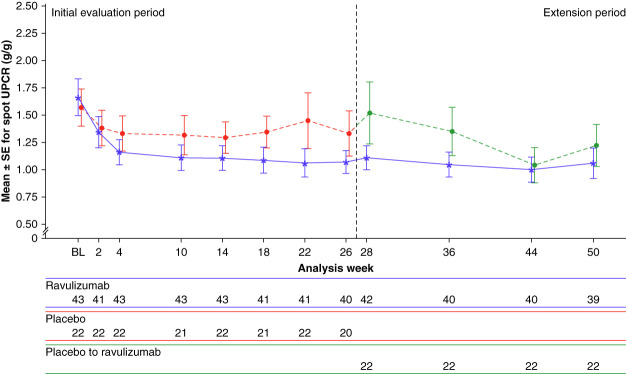
**Proteinuria reduction with ravulizumab occurred early and was sustained: mean (SEM) spot UPCR from baseline to week 50 by treatment group (exploratory end point).** BL, baseline.

At the end of the open-label extension period (week 50), the change from baseline in 24-hour UPCR with ravulizumab was −41.1% (95% CI, −51.7% to −28.3%, mean absolute change from 1.7 to 1.1 g/g) (Figure 3). Among patients who crossed over from placebo to ravulizumab at week 26, reduction from baseline (week 0) to week 50 was −43.1% (95% CI, −56.2% to −26.2%, mean absolute change from 1.8 to 1.1 g/g). The mean spot UPCR assessment over 50 weeks is shown in Figure 4.

**Figure 5:**
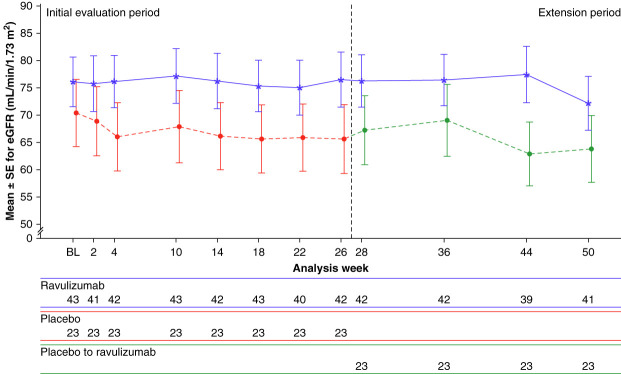
Mean (SEM) eGFR from baseline to week 50 by treatment group (secondary end point).

The eGFR over 50 weeks is shown in Figure 5. Mean (SD) eGFR values in the ravulizumab arm were 76 (30) ml/min per 1.73 m^2^ at baseline, 76 (33) ml/min per 1.73 m^2^ at week 26, and 72 (32) ml/min per 1.73 m^2^ at week 50; mean (SD) eGFR values in the placebo arm were 70 (30) ml/min per 1.73 m^2^ at baseline, 66 (30) ml/min per 1.73 m^2^ at week 26, and 64 (29) ml/min per 1.73 m^2^ at week 50 (following crossover to ravulizumab at week 26). Change from baseline in eGFR over time is presented in Supplemental Table 4. The annualized eGFR slope with ravulizumab was –1.35 ml/min per 1.73 m^2^ per year through 26 weeks and –2.34 ml/min per 1.73 m^2^ per year through 50 weeks. In patients on placebo, the annualized eGFR slope was –6.74 ml/min per 1.73 m^2^ per year through 26 weeks; after crossover to ravulizumab at week 26, the annualized slope was –4.09 ml/min per 1.73 m^2^ per year through 50 weeks (Table 2).

**Table 2:** Annualized slope of eGFR through week 26 and week 50 (exploratory end point)

Timepoint/Statistics	Ravulizumab (*n*=43)	Placebo to Week 26; Change to Ravulizumab Week 26–50 (*n*=23)
**Week 0–26**		
Slope of eGFR (ml/min per 1.73 m^2^ per year)	−1.35	−6.74
95% CI of slope	−5.51 to 2.81	−12.37 to −1.10
**Week 0–50**		
Slope of eGFR (ml/min per 1.73 m^2^ per year)	−2.34	−4.09
95% CI of slope	−4.94 to 0.26	−7.59 to −0.59

The slope of eGFR was computed using a mixed-effect model including data through week 26 or week 50. The model included eGFR as the response variable, random patient effects for intercepts and slopes, fixed categorical effects of treatment group, randomization stratification factor, and treatment group-by-visit interaction as well as a fixed, continuous effect of visit (study year). eGFR calculation is based on the Chronic Kidney Disease Epidemiology Collaboration formula. CI, confidence interval.

In the ravulizumab group, 44% (95% CI, 29% to 60%) achieved >50% reduction in proteinuria at week 26 (compared with 18% [95% CI, 5% to 40%] in the placebo group) (Supplemental Figure 2). Proteinuria <1.0 g/d was achieved in 27% (95% CI, 14% to 43%) and 18% (95% CI, 5% to 40%) of patients in the ravulizumab and placebo arms, respectively, at week 26 (Supplemental Figure 3). A statistically significant reduction in proteinuria with ravulizumab versus placebo was seen across several subgroups of patients, for example, proteinuria 1.0–2.0 and >2.0 g/d and eGFR >60–90 and >90 ml/min per 1.73 m^2^ (Supplemental Figure 4). The percentage change in urine albumin-creatinine ratio is listed in Supplemental Table 5. There was no change in mean serum C3 and C4 from baseline to week 26 (Supplemental Table 6). In a *post hoc* analysis, the percentage of patients with absence of hematuria increased over time with ravulizumab treatment (Supplemental Figure 5).

### Safety

Most of the AEs were mild and assessed by the investigator as not being related to study intervention (Table 3). One serious AE, involving hospitalization because of coronavirus disease 2019 infection, occurred in a patient in the ravulizumab arm; the event was assessed by the investigator as unrelated to ravulizumab and resolved during the study. There were no meningococcal infections, deaths, or AEs leading to withdrawal of the study drug reported during the study. Nasopharyngitis was the most common AE in patients treated with ravulizumab.

**Table 3:** Adverse events and safety outcomes during the initial evaluation and extension periods

Summary of AEs	Initial Evaluation Period		Extension Period	
	Ravulizumab, *n* (%), (*n*=43)	Placebo, *n* (%), (*n*=23)	Ravulizumab, *n* (%), (*n*=43)	Placebo to Ravulizumab, *n* (%), (*n*=23)
AE	32 (74)	19 (83)	26 (61)	10 (44)
SAE	1^a^ (2)	0 (0)	0 (0)	0 (0)
Death	0 (0)	0 (0)	0 (0)	0 (0)
AE/SAE leading to withdrawal of the study drug	0 (0)	0 (0)	0 (0)	0 (0)
**AE by relationship (investigator assessed)**				
Related	9 (21)	6 (26)	2 (5)	0 (0)
Not related	23 (54)	13 (57)	24 (56)	10 (44)
**AE by toxicity**				
Grade 1	25 (58)	13 (57)	18 (42)	7 (30)
Grade 2	7 (16)	5 (22)	8 (19)	3 (13)
Grade 3	0 (0)	1 (4)	0 (0)	0 (0)
Grade 4	0 (0)	0 (0)	0 (0)	0 (0)
Grade 5	0 (0)	0 (0)	0 (0)	0 (0)
**TEAEs occurring in ≥5% of patients in at least one of the treatment groups during each treatment period**				
Nasopharyngitis	5 (12)	1 (4)	6 (14)	0 (0)
COVID-19	4 (9)	0 (0)	3 (7)	2 (9)
Diarrhea	4 (9)	1 (4)	0 (0)	0 (0)
Cough	3 (7)	1 (4)	2 (5)	1 (4)
Fatigue	3 (7)	1 (4)	0 (0)	0 (0)
Influenza	2 (5)	2 (9)	1 (2)	0 (0)
Infusion-related reaction	2 (5)	2 (9)	0 (0)	1 (4)
Headache	1 (2)	1 (4)	3 (7)	0 (0)
Nausea	1 (2)	2 (9)	0 (0)	1 (4)
Peripheral edema	0 (0)	2 (9)	1 (2)	0 (0)
Pruritus	0 (0)	2 (9)	1 (2)	0 (0)
Rash	0 (0)	3 (13)	0 (0)	1 (4)

AE, adverse event; COVID-19, coronavirus disease 2019; SAE, serious adverse event; TEAE, treatment-emergent adverse event.

a Coronavirus disease 2019 infection was assessed by the investigator as unrelated to the study drug and resolved during the study.

In the ravulizumab arm, one patient had a treatment-boosted anti-drug antibody response and one patient had a transient treatment-emergent anti-drug antibody response. Neither patient had neutralizing antibodies. These anti-drug antibody responses were not associated with any adverse reactions and did not affect pharmacokinetics or efficacy. For further information, see the Supplemental Material.

### Pharmacokinetics and Pharmacodynamics

Throughout the study period, >99% of serum ravulizumab concentrations were above the target threshold of >175 *μ*g/ml (Supplemental Figure 6). Immediate and complete inhibition of terminal complement was observed with ravulizumab treatment because nearly all serum-free C5 concentrations were <0.5 *μ*g/ml (threshold for complete C5 inhibition) (Supplemental Figure 7).

## Discussion

In recent years, there has been increased optimism for new and more effective treatment options for patients with IgA nephropathy. Multiple pharmacological interventions are in clinical trials, targeting various points in the pathophysiology of IgA nephropathy. In this phase 2 trial of the complement C5 inhibitor ravulizumab, a statistically significant −30.1% (90% CI, −43.5% to −13.7%) treatment effect on proteinuria reduction versus placebo was observed at approximately 6 months. Importantly, the study population included patients already receiving the standard of care (RAS inhibition) and SGLT2 inhibitors (*n*=13). This reduction in proteinuria was sustained to the week 50 visit, and patients who crossed over from placebo to ravulizumab at week 26 had a similar decrease in proteinuria by week 50. These results suggest that ravulizumab may be a potential new treatment in IgA nephropathy, pending confirmation from a phase 3 trial.

Spot UPCR measurements indicated separation between ravulizumab and placebo as early as week 4, suggestive of an early treatment effect. This is indeed plausible considering the pharmacodynamic profile and evidence for the role of terminal complement activation in disease pathogenesis. With ravulizumab, less decline in eGFR slope versus placebo was observed at week 26. In patients who crossed over from placebo to ravulizumab at week 26, an attenuation of their earlier decline in eGFR slope was observed. Analyses by Inker *et al.* and Pitcher *et al.* have demonstrated the relationship between eGFR slope and long-term clinical outcomes in IgA nephropathy.^8,33^

Ravulizumab was well tolerated; no patients discontinued treatment because of AEs. Meningococcal infection caused by *Neisseria meningitidis* is a known risk with terminal complement inhibition, but this is mitigated by vaccination (and if needed, prophylactic antibiotics). Ravulizumab has been approved to treat four other conditions that require long-term treatment, leading to years of experience with a successful risk management program. In all patients receiving ravulizumab during this study, no cases of meningococcal infection occurred. There were also no cases of clinically relevant immunogenicity, and there was a low incidence of infusion-related reactions.

Despite advances in recent years, patients with IgA nephropathy are still progressing toward the eventual need for KRT. Therapeutic approaches under investigation for IgA nephropathy include complement inhibition and B-cell or plasma cell targeting to reduce the production of Gd-IgA1.^34–42^ In a phase 3 trial, the factor B (complement alternative pathway) inhibitor iptacopan reduced proteinuria by 38.3% versus placebo at 9 months.^34^ In phase 2 trials, sibeprenlimab, a humanized mAb to the cytokine “a proliferation-inducing ligand,” and the a proliferation-inducing ligand/B-cell–activating factor antagonist atacicept significantly reduced proteinuria.^37,38^

This analysis has the limitations inherent to any phase 2 trial, including sample size. No adjustments for multiplicity were conducted on secondary end points. Because 83% of patients were in either North America or Europe and most patients were White, geographic and racial generalizability of the results is limited. A global phase 3 trial of ravulizumab in patients with IgA nephropathy (I CAN; NCT06291376) is currently enrolling.

In conclusion, clinically meaningful reductions in proteinuria and a trend toward stabilization of eGFR were observed with ravulizumab. An early onset of effect was also seen—notable in a disease such as IgA nephropathy—where time with ongoing disease activity is associated with irreversible loss of organ function. Safety data were generally similar in patients treated with ravulizumab or placebo and consistent with the safety profile from years of use of ravulizumab in other diseases. Data from the phase 3 trial will provide further insight into terminal complement inhibition in IgA nephropathy with ravulizumab. In the future, complement inhibition may be used for patients at high risk of disease progression as a therapeutic option for reducing immune complex–mediated inflammation in the kidney. Evolving therapeutic options targeting different pathogenic drivers—or combinations thereof—may bring about advances in the management of a disease that continues to reduce the quality and expectancy of life for too many people worldwide.

## Supplementary Material

## Data Availability

Partial restrictions to the data and/or materials apply. Alexion, AstraZeneca Rare Disease will consider requests for disclosure of clinical study participant-level data provided that participant privacy is assured through methods like data deidentification, pseudonymization, or anonymization (as required by applicable law), and if such disclosure was included in the relevant study informed consent form or similar documentation. Qualified academic investigators may request participant-level clinical data and supporting documents (statistical analysis plan and protocol) pertaining to Alexion-sponsored studies. Further details regarding data availability and instructions for requesting information are available in the Alexion Clinical Trials Disclosure and Transparency Policy at https://www.alexionclinicaltrialtransparency.com/data-requests.

## Supplemental Material

Supplemental Summary 1. List of study investigators.

Study Methodology. Additional detail

Supplemental Results. Immunogenicity.

Supplemental Figure 1. Study design.

Supplemental Figure 2. Percentage of patients with >30% and >50% reduction in proteinuria. (A) Percentage of patients with >30% and >50% proteinuria reduction on the basis of 24-hour urine protein (g/d) at week 26 (secondary end point). (B) Percentage of patients with >30% and >50% proteinuria reduction on the basis of 24-hour urine protein (g/d) at week 50 (secondary end point).

Supplemental Figure 3. Percentage of patients meeting the criteria for partial remission (<1 g/d) on the basis of 24-hour urine protein at week 26 and week 50 (secondary end point).

Supplemental Figure 4. A statistically significant reduction in proteinuria with ravulizumab versus placebo was seen across several subgroups of patients: difference in change in proteinuria (and 90% confidence interval) from baseline to week 26, ravulizumab versus placebo in subgroups of patients (on the basis of 24-hour urine protein) (exploratory end point).

Supplemental Figure 5. Percentage of participants with absence of hematuria: ≤5 red blood cells/high-power field on urine sediment from spot samples reported by the central lab, from baseline to week 50 (*post hoc* analysis).

Supplemental Figure 6. Pharmacokinetics: mean±SD ravulizumab concentration over time by treatment group.

Supplemental Figure 7. Pharmacodynamics: serum-free C5 concentration over time by treatment group.

Supplemental Table 1. Weight-based dosing of ravulizumab.

Supplemental Table 2. Baseline biopsy characteristics and MEST-C scores in ravulizumab and placebo groups.

Supplemental Table 3. Baseline complement staining in ravulizumab and placebo groups.

Supplemental Table 4. Change from baseline in eGFR over time, by treatment group (secondary end point).

Supplemental Table 5. Percentage change from baseline in 24-hour urine albumin-creatinine ratio (g/mol) (exploratory end point).

Supplemental Table 6. Change from baseline to week 26 in serum C3 and C4 (secondary end point).

Plain Language Summary

Statistical Analysis Plan
