# Supplementary material for: Efficacy and Safety of Ravulizumab in IgA Nephropathy: A Phase 2 Randomized Double-Blind Placebo-Controlled Trial
Source: J Am Soc Nephrol. 2024 Oct 25;36(4):645–56. doi: 10.1681/ASN.0000000534 (PMC11975245; doi:10.1681/ASN.0000000534)
Supplement: Supplementary file 1 [file jasn-36-645-s001.pdf]

ASN American Society of Nephrology
JASN Journal of the American Society of Nephrology
KIDNEY360 Accessing Our World From Every Angle
CJASN Clinical Journal of the American Society of Nephrology

ASN Journal Disclosure Form

As per ASN journal policy, I have disclosed any financial relationships or commitments I have held in the past 36 months as included below. I have listed my Current Employer below to indicate there is a relationship requiring disclosure. If no relationship exists, my Current Employer is not listed.

E. Alamartine reports the following:
Employer: CHU SAINT-ETIENNE; and Consultancy: ALEXION; OTSUKA.

I understand that the information above will be published within the journal article, if accepted, and that failure to comply and/or to accurately and completely report the potential financial conflicts of interest could lead to the following: 1) Prior to publication, article rejection, or 2) Post-publication, sanctions ranging from, but not limited to, issuing a correction, reporting the inaccurate information to the authors' institution, banning authors from submitting work to ASN journals for varying lengths of time, and/or retraction of the published work.

Name: Eric Alamartine
Manuscript ID: JASN-2024-001106
Manuscript Title: LBCT: Efficacy and Safety of Ravulizumab in IgA Nephropathy: Results of a Phase 2 Randomized Double-Blind Placebo-Controlled Trial
Date of Completion: August 29, 2024
Disclosure Updated Date: August 29, 2024

ASN American Society of Nephrology | JASN Journal of the American Society of Nephrology | KIDNEY360 Accessing Our World From Every Angle | CJASN Clinical Journal of the American Society of Nephrology

ASN Journal Disclosure Form

As per ASN journal policy, I have disclosed any financial relationships or commitments I have held in the past 36 months as included below. I have listed my Current Employer below to indicate there is a relationship requiring disclosure. If no relationship exists, my Current Employer is not listed.

J. Barratt reports the following:
Employer: UNIVERSITY OF LEICESTER; Consultancy: Alexion, Astellas, Alebund, Alnylam, Alpine, Argenx, BioCryst, Calliditas, Chinook, Dimerix, HiBio, Kira, Novartis, Omeros, Otsuka, Travere Therapeutics, Q32 Bio, Roche, Sanofi, Takeda, Vera Therapeutics, Vifor, Visterra; Research Funding: Alexion, Novartis; GlaxoSmithKline; Calliditas, Visterra, Chinook, Omeros, Galapagos, argenx, Travere Therapeutics; and Advisory or Leadership Role: Editorial Board of Kidney International, CJASN, Glomerular Diseases & Clinical Science; Treasurer International IgA Nephropathy Network.

I understand that the information above will be published within the journal article, if accepted, and that failure to comply and/or to accurately and completely report the potential financial conflicts of interest could lead to the following: 1) Prior to publication, article rejection, or 2) Post-publication, sanctions ranging from, but not limited to, issuing a correction, reporting the inaccurate information to the authors' institution, banning authors from submitting work to ASN journals for varying lengths of time, and/or retraction of the published work.

Name: Jonathan Barratt
Manuscript ID: JASN-2024-001106
Manuscript Title: LBCT: Efficacy and Safety of Ravulizumab in IgA Nephropathy: Results of a Phase 2 Randomized Double-Blind Placebo-Controlled Trial
Date of Completion: August 28, 2024
Disclosure Updated Date: August 28, 2024

ASN American Society of Nephrology
JASN Journal of the American Society of Nephrology
KIDNEY360 Accessing Our World From Every Angle
CJASN Clinical Journal of the American Society of Nephrology

ASN Journal Disclosure Form

As per ASN journal policy, I have disclosed any financial relationships or commitments I have held in the past 36 months as included below. I have listed my Current Employer below to indicate there is a relationship requiring disclosure. If no relationship exists, my Current Employer is not listed.

R. Fenoglio reports the following:
Employer: San Giovanni Hospital, Turin

I understand that the information above will be published within the journal article, if accepted, and that failure to comply and/or to accurately and completely report the potential financial conflicts of interest could lead to the following: 1) Prior to publication, article rejection, or 2) Post-publication, sanctions ranging from, but not limited to, issuing a correction, reporting the inaccurate information to the authors' institution, banning authors from submitting work to ASN journals for varying lengths of time, and/or retraction of the published work.

Name: Roberta Fenoglio
Manuscript ID: JASN-2024-001106
Manuscript Title: LBCT: Efficacy and Safety of Ravulizumab in IgA Nephropathy: Results of a Phase 2 Randomized Double-Blind Placebo-Controlled Trial
Date of Completion: August 30, 2024
Disclosure Updated Date: August 20, 2024

ASN
American Society of Nephrology
JASN
Journal of the American Society of Nephrology
KIDNEY360
Accessing Our World From Every Angle
CJASN
Clinical Journal of the American Society of Nephrology

ASN Journal Disclosure Form

As per ASN journal policy, I have disclosed any financial relationships or commitments I have held in the past 36 months as included below. I have listed my Current Employer below to indicate there is a relationship requiring disclosure. If no relationship exists, my Current Employer is not listed.

K. Garlo reports the following:
Employer: Alexion rare disease unit of AstraZeneca Pharmaceuticals; and Ownership Interest: Alexion/AZ as above.

I understand that the information above will be published within the journal article, if accepted, and that failure to comply and/or to accurately and completely report the potential financial conflicts of interest could lead to the following: 1) Prior to publication, article rejection, or 2) Post-publication, sanctions ranging from, but not limited to, issuing a correction, reporting the inaccurate information to the authors' institution, banning authors from submitting work to ASN journals for varying lengths of time, and/or retraction of the published work.

Name: Katherine Garlo
Manuscript ID: JASN-2024-001106R1
Manuscript Title: LBCT: Efficacy and Safety of Ravulizumab in IgA Nephropathy: A Phase 2 Randomized Double-Blind Placebo-Controlled Trial
Date of Completion: September 25, 2024
Disclosure Updated Date: April 11, 2024

ASN American Society of Nephrology
JASN Journal of the American Society of Nephrology
KIDNEY360 Accessing Our World From Every Angle
CJASN Clinical Journal of the American Society of Nephrology

ASN Journal Disclosure Form

As per ASN journal policy, I have disclosed any financial relationships or commitments I have held in the past 36 months as included below. I have listed my Current Employer below to indicate there is a relationship requiring disclosure. If no relationship exists, my Current Employer is not listed.

S. Huang reports the following:
Employer: London Health Sciences Centre; Honoraria: Alexion; Sanofi; Ultragenic; and Speakers Bureau: Alexion;.

I understand that the information above will be published within the journal article, if accepted, and that failure to comply and/or to accurately and completely report the potential financial conflicts of interest could lead to the following: 1) Prior to publication, article rejection, or 2) Post-publication, sanctions ranging from, but not limited to, issuing a correction, reporting the inaccurate information to the authors' institution, banning authors from submitting work to ASN journals for varying lengths of time, and/or retraction of the published work.

Name: Shih-Han S. Huang
Manuscript ID: JASN-2024-001106
Manuscript Title: LBCT: Efficacy and Safety of Ravulizumab in IgA Nephropathy: Results of a Phase 2 Randomized Double-Blind Placebo-Controlled Trial
Date of Completion: August 28, 2024
Disclosure Updated Date: August 28, 2024

ASN American Society of Nephrology | JASN Journal of the American Society of Nephrology | KIDNEY360 Accessing Our World From Every Angle | CJASN Clinical Journal of the American Society of Nephrology

ASN Journal Disclosure Form

As per ASN journal policy, I have disclosed any financial relationships or commitments I have held in the past 36 months as included below. I have listed my Current Employer below to indicate there is a relationship requiring disclosure. If no relationship exists, my Current Employer is not listed.

A. Kateifides reports the following:
Employer: Alexion AstraZeneca Rare Disease, Vertex Pharmaceuticals; and Ownership Interest: AstraZeneca; Vertex Pharmaceuticals.

I understand that the information above will be published within the journal article, if accepted, and that failure to comply and/or to accurately and completely report the potential financial conflicts of interest could lead to the following: 1) Prior to publication, article rejection, or 2) Post-publication, sanctions ranging from, but not limited to, issuing a correction, reporting the inaccurate information to the authors' institution, banning authors from submitting work to ASN journals for varying lengths of time, and/or retraction of the published work.

Name: Andreas Kateifides
Manuscript ID: JASN-2024-001106
Manuscript Title: LBCT: Efficacy and Safety of Ravulizumab in IgA Nephropathy: Results of a Phase 2 Randomized Double-Blind Placebo-Controlled Trial
Date of Completion: September 16, 2024
Disclosure Updated Date: September 16, 2024

ASN Journal Disclosure Form

As per ASN journal policy, I have disclosed any financial relationships or commitments I have held in the past 36 months as included below. I have listed my Current Employer below to indicate there is a relationship requiring disclosure. If no relationship exists, my Current Employer is not listed.

J. Kaufeld reports the following:
Employer: Medical School of Hannover; and Honoraria: Amicus Therapeutics; Sanofi; Chiesi Pharma, Takeda, Alexion, Novartis, AstraZeneca.

I understand that the information above will be published within the journal article, if accepted, and that failure to comply and/or to accurately and completely report the potential financial conflicts of interest could lead to the following: 1) Prior to publication, article rejection, or 2) Post-publication, sanctions ranging from, but not limited to, issuing a correction, reporting the inaccurate information to the authors' institution, banning authors from submitting work to ASN journals for varying lengths of time, and/or retraction of the published work.

Name: Jessica Katharina Kaufeld
Manuscript ID: JASN-2024-001106R1
Manuscript Title: LBCT: Efficacy and Safety of Ravulizumab in IgA Nephropathy: A Phase 2 Randomized Double-Blind Placebo-Controlled Trial
Date of Completion: September 26, 2024
Disclosure Updated Date: September 26, 2024

ASN American Society of Nephrology JASN Journal of the American Society of Nephrology KIDNEY360 Accessing Our World From Every Angle CJASN Clinical Journal of the American Society of Nephrology

ASN Journal Disclosure Form

As per ASN journal policy, I have disclosed any financial relationships or commitments I have held in the past 36 months as included below. I have listed my Current Employer below to indicate there is a relationship requiring disclosure. If no relationship exists, my Current Employer is not listed.

S. Kim reports the following:
Employer: Hallym University Sacred Heart Hospital; Consultancy: Alpine; AstraZeneca; Billy; GSK; Alexion; Bayer; Research Funding: Fibrogen; GSK; JW; VALOR; Alexion; Roche; Bayer;; Honoraria: GSK; bayer; Alpine; Alexion; and Advisory or Leadership Role: KSN.

I understand that the information above will be published within the journal article, if accepted, and that failure to comply and/or to accurately and completely report the potential financial conflicts of interest could lead to the following: 1) Prior to publication, article rejection, or 2) Post-publication, sanctions ranging from, but not limited to, issuing a correction, reporting the inaccurate information to the authors' institution, banning authors from submitting work to ASN journals for varying lengths of time, and/or retraction of the published work.

Name: Sung Gyun Kim
Manuscript ID: JASN-2024-001106R1
Manuscript Title: LBCT: Efficacy and Safety of Ravulizumab in IgA Nephropathy: A Phase 2 Randomized Double-Blind Placebo-Controlled Trial
Date of Completion: September 25, 2024
Disclosure Updated Date: September 13, 2024

ASN American Society of Nephrology
JASN Journal of the American Society of Nephrology
KIDNEY360 Accessing Our World From Every Angle
CJASN Clinical Journal of the American Society of Nephrology

ASN Journal Disclosure Form

As per ASN journal policy, I have disclosed any financial relationships or commitments I have held in the past 36 months as included below. I have listed my Current Employer below to indicate there is a relationship requiring disclosure. If no relationship exists, my Current Employer is not listed.

R. Lafayette reports the following:
Employer: Stanford University; Consultancy: Calliditas, Inc; Chinook, Inc: Omeros, Inc; Otsuka, Inc. ; Alexion, Inc, Travere, Inc., Vera, inc. , Novartis, Aurinia; Visterra, Alpine Bio, Beigene.; and Research Funding: NIH, Pfizer, Roche, Amgen, Otsuka, Omeros, Calliditas, Travere, Apellis, Chinook, Vera, Beigene, .

I understand that the information above will be published within the journal article, if accepted, and that failure to comply and/or to accurately and completely report the potential financial conflicts of interest could lead to the following: 1) Prior to publication, article rejection, or 2) Post-publication, sanctions ranging from, but not limited to, issuing a correction, reporting the inaccurate information to the authors' institution, banning authors from submitting work to ASN journals for varying lengths of time, and/or retraction of the published work.

Name: Richard A. Lafayette
Manuscript ID: 2024-001106
Manuscript Title: LBCT: Efficacy and Safety of Ravulizumab in IgA Nephropathy: Results of a Phase 2 Randomized Double-Blind Placebo-Controlled Trial
Date of Completion: September 16, 2024
Disclosure Updated Date: May 1, 2024

ASN American Society of Nephrology · JASN Journal of the American Society of Nephrology · KIDNEY360 Accessing Our World From Every Angle · CJASN Clinical Journal of the American Society of Nephrology

ASN Journal Disclosure Form

As per ASN journal policy, I have disclosed any financial relationships or commitments I have held in the past 36 months as included below. I have listed my Current Employer below to indicate there is a relationship requiring disclosure. If no relationship exists, my Current Employer is not listed.

M. Pérez valdivia reports the following:
Employer: Hospital Virgen del Rocío; Consultancy: Alexion; and Advisory or Leadership Role: Vifor.

I understand that the information above will be published within the journal article, if accepted, and that failure to comply and/or to accurately and completely report the potential financial conflicts of interest could lead to the following: 1) Prior to publication, article rejection, or 2) Post-publication, sanctions ranging from, but not limited to, issuing a correction, reporting the inaccurate information to the authors' institution, banning authors from submitting work to ASN journals for varying lengths of time, and/or retraction of the published work.

Name: Miguel angel Pérez valdivia
Manuscript ID: JASN-2024-001106
Manuscript Title: Efficacy and Safety of Ravulizumab in IgA Nephropathy: Results of a Phase 2 Randomized Double-Blind Placebo-Controlled Tria
Date of Completion: August 29, 2024
Disclosure Updated Date: August 29, 2024

ASN American Society of Nephrology | JASN Journal of the American Society of Nephrology | KIDNEY360 Accessing Our World From Every Angle | CJASN Clinical Journal of the American Society of Nephrology

ASN Journal Disclosure Form

As per ASN journal policy, I have disclosed any financial relationships or commitments I have held in the past 36 months as included below. I have listed my Current Employer below to indicate there is a relationship requiring disclosure. If no relationship exists, my Current Employer is not listed.

K. Rice reports the following:
Employer: Alexion Pharmaceuticals; AstraZeneca; and Ownership Interest: Alexion Pharmaceuticals; AstraZeneca.

I understand that the information above will be published within the journal article, if accepted, and that failure to comply and/or to accurately and completely report the potential financial conflicts of interest could lead to the following: 1) Prior to publication, article rejection, or 2) Post-publication, sanctions ranging from, but not limited to, issuing a correction, reporting the inaccurate information to the authors' institution, banning authors from submitting work to ASN journals for varying lengths of time, and/or retraction of the published work.

Name: Kara Rice
Manuscript ID: JASN-2024-001106
Manuscript Title: LBCT: Efficacy and Safety of Ravulizumab in IgA Nephropathy: Results of a Phase 2 Randomized Double-Blind Placebo-Controlled Trial
Date of Completion: August 28, 2024
Disclosure Updated Date: April 30, 2024

ASN American Society of Nephrology
JASN Journal of the American Society of Nephrology
KIDNEY360 Accessing Our World From Every Angle
CJASN Clinical Journal of the American Society of Nephrology

ASN Journal Disclosure Form

As per ASN journal policy, I have disclosed any financial relationships or commitments I have held in the past 36 months as included below. I have listed my Current Employer below to indicate there is a relationship requiring disclosure. If no relationship exists, my Current Employer is not listed.

J. Tumlin reports the following:
Employer: NephroNet Clinical Trials Consortium; Consultancy: NephroNet Clinical Trials Consortium, Mallinckrodt Pharmaceuticals, Liliy Pharmaceuticals, Relypsa Pharmaceuticals, Astra Zeneca Pharmaceuticals, Alexion Pharmaceuticals, Astra Zeneca Pharmaceuticals, Alpine Pharmaceuticals, Vera Pharmaceuticals, Vertex Therapeutics. Alexion Pharmaceuticals, HIBAR Microsciences LLC, Calliditas Therapeutics. Travere Pharmaceuticals.; Research Funding: NephroNet Clinical Trials Consortium, Mallinckrodt Pharmaceuticals, Johnson& Johnson, LaJolla Pharmaceuticals, Abbvie Pharmaceuticals, ZS Pharmaceuticals. Achillion Pharmaceuticals, Relypsa Pharmaceuticals, Epizon Pharmaceuticals, Gilead Pharmaceuticals, Astra Zeneca; Akebia Pharmaceuticals. Vera Pharmaceuticals., Alpine Pharmaceuticals, Horizon Pharmaceuticals; Novartis Pharmaceuticals; Honoraria: Mallinckrodt Pharmaceuticals, Genentech, Alexion Pharmaceuticals, Genzyme Corp. Astra Zeneca Pharmaceuticals.; Aurinia Pharmaceuticals; Astra Zeneca Pharmaceuticals. Bayer Pharmaceuticals Alpine Pharmaceuticals, Calliditis Therapeutics; Patents or Royalties: HIBAR Microsciences LLC; Advisory or Leadership Role: Achillion Pharmaceuticals, Relypsa Pharmaceuticals, Epizon Pharmaceuticals, Gilead Pharmaceuticals, KBP Pharmaceuticals.; Alexion Pharmaceuticals; Bayer Pharmaceuticals, Chemocentryx and Bayer Corp. Aurinia Pharmaceuticals, Vera Therapeutics, Novartis; and Speakers Bureau: Mallinckrodt, La Jolla Pharmaceuticals and Alexion Pharmaceuticals, Astra Zeneca Pharmaceuticals; Aurinia Pharmaceuticals Bayer Pharmaceuticals, Bayer Corporation, Callidatis Pharmaceuticals Travere Pharmaceuticals.

I understand that the information above will be published within the journal article, if accepted, and that failure to comply and/or to accurately and completely report the potential financial conflicts of interest could lead to the following: 1) Prior to publication, article rejection, or 2) Post-publication, sanctions ranging from, but not limited to, issuing a correction, reporting the inaccurate information to the authors' institution, banning authors from submitting work to ASN journals for varying lengths of time, and/or retraction of the published work.

Name: James A. Tumlin
Manuscript ID: JASN-2024-001106
Manuscript Title: "LBCT: Efficacy and Safety of Ravulizumab in IgA Nephropathy: Results of a Phase 2 Randomized Double-Blind Placebo-Controlled Trial,"
Date of Completion: August 28, 2024
Disclosure Updated Date: August 28, 2024

ASN
American Society of Nephrology
JASN
Journal of the American Society of Nephrology
KIDNEY360
Accessing Our World From Every Angle
CJASN
Clinical Journal of the American Society of Nephrology

ASN Journal Disclosure Form

As per ASN journal policy, I have disclosed any financial relationships or commitments I have held in the past 36 months as included below. I have listed my Current Employer below to indicate there is a relationship requiring disclosure. If no relationship exists, my Current Employer is not listed.

M. Wu reports the following:
Employer: Taipei Medical University; Consultancy: AZ; and Honoraria: AZ; Novartis; Takeda; Sanofi; Baxter.

I understand that the information above will be published within the journal article, if accepted, and that failure to comply and/or to accurately and completely report the potential financial conflicts of interest could lead to the following: 1) Prior to publication, article rejection, or 2) Post-publication, sanctions ranging from, but not limited to, issuing a correction, reporting the inaccurate information to the authors' institution, banning authors from submitting work to ASN journals for varying lengths of time, and/or retraction of the published work.

Name: Mai-Szu Wu
Manuscript ID: JASN-2024-001106
Manuscript Title: Efficacy and Safety of Ravulizumab in IgA Nephropathy: Results of a Phase 2 Randomized Double-Blind Placebo-Controlled Trial
Date of Completion: August 28, 2024
Disclosure Updated Date: August 28, 2024

ASN American Society of Nephrology | JASN Journal of the American Society of Nephrology | KIDNEY360 Accessing Our World From Every Angle | CJASN Clinical Journal of the American Society of Nephrology

ASN Journal Disclosure Form

As per ASN journal policy, I have disclosed any financial relationships or commitments I have held in the past 36 months as included below. I have listed my Current Employer below to indicate there is a relationship requiring disclosure. If no relationship exists, my Current Employer is not listed.

M. Yee reports the following:
Employer: Alexion, AstraZeneca Rare Disease; and Ownership Interest: Alexion, AstraZeneca Rare Disease.

I understand that the information above will be published within the journal article, if accepted, and that failure to comply and/or to accurately and completely report the potential financial conflicts of interest could lead to the following: 1) Prior to publication, article rejection, or 2) Post-publication, sanctions ranging from, but not limited to, issuing a correction, reporting the inaccurate information to the authors' institution, banning authors from submitting work to ASN journals for varying lengths of time, and/or retraction of the published work.

Name: Min Yee
Manuscript ID: JASN-2024-001106
Manuscript Title: Efficacy and Safety of Ravulizumab in IgA Nephropathy: Results of a Phase 2 Randomized Double-Blind Placebo-Controlled Trial
Date of Completion: August 28, 2024
Disclosure Updated Date: August 28, 2024
