# Supplementary material for: Efficacy and Safety of Ravulizumab in IgA Nephropathy: A Phase 2 Randomized Double-Blind Placebo-Controlled Trial
Source: J Am Soc Nephrol. 2024 Oct 25;36(4):645–56. doi: 10.1681/ASN.0000000534 (PMC11975245; doi:10.1681/ASN.0000000534)
Supplement: Supplementary file 2 [file jasn-36-645-s002.pdf]

Supplemental Material

Supplemental Material Table of Contents

List of Study Investigators

Study Methodology: Additional Detail

Supplemental Results – Immunogenicity

Supplemental Figure 1. Study design

Supplemental Figure 2. Percentage of patients with >30% and >50% reduction in proteinuria

A) Percentage of patients with >30% and >50% proteinuria reduction based on 24-hour urine protein (g/day) at week 26 (secondary endpoint)

B) Percentage of patients with >30% and >50% proteinuria reduction based on 24-hour urine protein (g/day) at week 50 (secondary endpoint)

Supplemental Figure 3. Percentage of patients meeting the criteria for partial remission (<1 g/day) based on 24-hour urine protein at week 26 and week 50 (secondary endpoint).

Supplemental Figure 4. A statistically significant reduction in proteinuria with ravulizumab versus placebo was seen across several subgroups of patients: Difference in change in proteinuria (and 90% confidence interval) from baseline to week 26, ravulizumab versus placebo in subgroups of patients (based on 24-hour urine protein) (exploratory endpoint).

Supplemental Figure 5. Percentage of participants with absence of hematuria: ≤5 red blood cells/high-power field on urine sediment from spot samples reported by the central lab, from baseline to week 50 (*post hoc* analysis)

Supplemental Figure 6. Pharmacokinetics: mean (standard deviation) ravulizumab concentration over time by treatment group

Supplemental Figure 7. Pharmacodynamics: serum free C5 concentration over time by treatment group

Supplemental Table 1. Weight-based dosing of ravulizumab

Supplemental Table 2. Baseline biopsy characteristics and MEST-C scores in ravulizumab and placebo groups

Supplemental Table 3. Baseline complement staining in ravulizumab and placebo groups

Supplemental Table 4. Change from baseline in eGFR from over time, by treatment group (secondary endpoint)

Supplemental Table 5. Percentage change from baseline in 24-hour urine albumin-to-creatinine ratio (g/mol) (exploratory endpoint)

Supplemental Table 6. Change from baseline to week 26 in serum C3 and C4 (secondary endpoint)

Plain Language Summary

Statistical Analysis Plan

List of Study Investigators

Sites and investigators who randomized patients

Australia: Dwarakanathan Ranganathan (PI), Martin Wolley (Royal Brisbane and Women' Hospital, Herston); Sven-Jean Tan (PI), Kevin Chow, Mandy Man Tin Law, Kathleen Nichols, Emily Jan See, Adam Glenn Steinberg, Mark Tiong (The Royal Melbourne Hospital, Parkville)

Canada: Shih-Han Susan Huang (PI), Arsh Jain (London Health Sciences Center, East London, Ontario); Louis-Phillipe Laurin (PI), Jean-Phillipe Lafrance, Caroline Lamarche (Hopital Masonneuve – Rosemont, Montreal, Quebec); David Philibert (PI), Mohsen Agharazii, Fabrice Mac-Way (CHU de Quebec-Université Laval, Quebec City, Quebec)

France: Eric Alamartine (PI), Nicolas Maillard, Christophe Mariat, Ingrid Masson (Hôpital Nord CHU Saint Etienne, Saint Etienne); Jean-Jacques Boffa (PI), Alexandre Cez, Emmanuel Esteve, Armance Marchal (Hôpital Tenon, Paris); Dominique Chauveau (PI), Julie Belliere, Pauline Bernadet Monrozeis, Eloise Colliou, Stanislas Faguer, Antoine Huart, David Ribes (CHU-Hôpital Rangueil, Toulouse); Cyril Garrouste (PI), Anne-Elisabeth Heng, Valentin Mayet, Carole Philipponnet (CHU Gabriel Montpied, Clermont-Ferrand); Bruno Moulin (PI), Laura Braun Parvez, Gabriela Gautier Vargas, Peggy Perrin, Sophie Ohlmann, Jerome Olagne (University Hospital of Strasbourg, France)

Germany: Herman Haller (PI), Kai Schmidt-Ott (PI), Elisabeth Bahlmann-Kroll, Jessica Kaufeld, Martin Reinhardt (Medizinische Hochschule Hannover); Anja Gaeckler (PI), Sebastian Dolff, Benjamin Wilde (Universitaetsklinikum Essen); Adrian Schreiber (PI), Nadine Koch, Evelyn Seelow (Charité-Universitätsmedizin Berlin)

Italy: Federico Alberici (PI), Stefania Affatato, Allesandro Mainardi, Federica Mescia, Francesco Scolari, Martina Tedesco, Margherita Venturini (Azienda Socio Sanitaria Territoriale degli Spedali Civili di Brescia); Calogero Lino Cirami (PI), Giulia Antognoli, Leonardo Caroti, Gabriele Gualtiero, Marcos Moscato, Giorgio Trivioli (Azienda Ospedaliero-Universitaria Careggi, Florence); Gaetano La Manna (PI), Olga Baraldi, Anita Campus, Carlo Stefanini, Gisella Vischini (Policlinico S. Orsola Malpighi, Bologna); Dario Roccatello (PI), Roberta Fenoglio, Stefania Lalloni, Savino Sciascia (San Giovanni Bosco Hub Hospital, ASL Città di Torino and Department of Clinical and Biological Sciences of the University of Turin, Turin)

Poland: Michal Nowicki (PI), Tomasz Holub, Błażej Kieszek, Agnieszka Makówka, Anna Masajtis, Piotr Piechota, Maciej Tylski (Centralny Szpital Kliniczny Uniwersytetu Medycznego w Lodzi)

South Korea: Seung Hyeok Han (PI), Sang-Won Lee, Beon Jin Lim, Jung Yoon Pyo (Severance Hospital – Yonsei University College of Medicine, Seoul; Sung Gyun Kim (PI), Jung Nam An, Byung Ha Chung, Jwa-Kyung Kim, Hyung Seok Lee, Jung Yoon Pyo, Young Il Seo, Young Rim Song (Hallym University Sacred Heart Hospital, Anyang); Hajeong Lee, Sehoon Park (PI), Seung Seok Han, Dong Ki Kim, Yong Chul Kim, Hyunjin Ryu (Seoul National University Hospital)

Spain: Irene Agraz (PI), Sheila Bermejo, Monica Bolufer, Josefina Cortés Hernandez, Maria Alejandra Gabaldon, Maria José Soler (Hospital Universitari Vall d'Hebron, Barcelona); David Arroyo (PI), Javier Carbayo, Marian Goicoechea Diezhandino, Ana Perez de Jose, Ursula Verdalles Guzman, Eduardo Verde Moreno (Hospital General Universitario Gregorio Marañón, Madrid); Olga Gracia García (PI), Zoila Stany Albines Fiestas, Daniel Joaquin Aladren Gonzalvo, Andrea Bernal Gonzalez, Luis Miguel Lou Arnal, Cristina Medrano Villarroya, Paula Mora Lopez, Eduardo Parra Moncasi (Hospital Universitario Miguel Servet, Zaragoza); Maria Luisa Martin Conde (PI), Jorge Gonzalez, Elias Jatem, Aida Moroba Estor, Alfons Segarra Medrano (Hospital Universitario Arnau de Vilanova, Lleida);

Antolina Rodriguez Moreno (PI), Dolores Sanchez de la Nieta, Clara Garcia, Mercedes Velo (Hospital Clinico San Carlos, Madrid); Jose Luis Rocha Castilla (PI), Sheila Bermejo, Manuel Lopez Mendoza, Miguel Angel Pérez Valdivia (Hospital Virgen del Rocío, Seville); Remedios Toledo Rojas (PI), Pilar Hidalgo, Teresa Vazquez Sanchez (Hospital Regional Universitario de Mlaga Carlos Haya, Malaga); Miguel Giovanni Uriol Rivera (PI), Mario Lado Fuentes (Hospital Universitari Son Espases, Palma, Illes Balears)

Taiwan: Yi-Wen Chiu (PI), Jer-Ming Chang, Hung-Chun Chen, Mei-Ju Hsiao, Chi-Chih Hung, Shang-Jih Hwang, Mei Chuan Kuo, Hung-Tien Kuo, Lee-Moay Lim, Jia-Jung Lee (Kaohsiung Medical University Chung-Ho Memorial Hospital, Kaohsiung City); Ping Chin Lai (PI), David Ray Chang, Yun-Lun Chang, I-Ru Chen, Ming-Han Hsieh, Chiu-Ching Huang, Huey-Liang Kuo, I-Kuan Wang (China Medical University Hospital, Taichung City); Mai-Szu Wu (PI), Yu-Wei Chen, I-Jen Chiu, Yung-Ho Hsu, Lie-Yee Hung, Chia-Te Laio, Gua-Hong Lin, Yuh-Feng Lin, Mei-Yi Wu, Cai-Mei Zheng (Shuang Ho Hospital, New Taipei City)

United Kingdom: Liz Lightstone (PI), Tommy Cairns, Stephen Mcadoo, Nicholas Medjeral-Thomas, Matthew Pickering (Imperial College London); Michael Robson (PI) (King's College London); Smeeta Sinha (PI), Chukwuma Chukwu, Joshua Storrar (Salford Royal NHS Foundation Trust, University of Manchester)

United States: Sadiq Ahmed (PI), Virgilius Cornea, Amina Nadim, Tyler Sims (University of Kentucky); Ahmed Awad (PI), Mohammed Aldahan, Laurie Clark, Ryan Lustig (Clinical Research Consultants LLC, Kansas City, Missouri); John Niles (PI), Anushya Jeyabalan, Reza Zonozi (Massachusetts General Hospital); James Tumlin (PI), William Paxton (Emory University, Atlanta, Georgia)

Study Methodology: Additional Detail

Eligibility Criteria

Inclusion Criteria

1. Aged 18–75 years
2. Body weight ≥40 kg at screening
3. Male or female. Female participants of childbearing potential, male participants, and male participants with female partners of childbearing potential must follow protocol specified contraception guidance
4. Capable of giving informed consent
5. To reduce the risk of meningococcal infection (*N. meningitidis*), all participants must be vaccinated against meningococcal infection from serogroups A, C, W, Y, and B within 3 years prior to, or at the time of, randomization according to national/local guidelines. Participants who do not meet this requirement will be vaccinated against meningococcal infection prior to randomization according to national/local guidelines and will receive prophylactic antibiotics for at least 2 weeks after meningococcal vaccination if randomization occurs < 2 weeks after initial vaccination.
6. All participants must also receive vaccinations for *Haemophilus influenzae* type B (Hib) and *Streptococcus pneumoniae* prior to randomization, unless previously vaccinated, according to current national/local vaccination guidelines
7. Local pathology report from the biopsy used for diagnosis must be available
8. Participants on sodium-glucose cotransporter-2 (SGLT-2) inhibitors (eg, empagliflozin) must be on a stable dose for ≥ 3 months with no planned change in dose during the study

Inclusion Criteria Specific for Lupus Nephritis (LN) Cohort

9. Clinical diagnosis of systemic lupus erythematosus (SLE) by 2019 ACR and EULAR criteria

10. Diagnosis of 2018 Revised ISN/RPS classification (active focal or diffuse proliferative LN Class III or IV confirmed by biopsy obtained ≤ 6 months prior to Screening or during Screening Period. Participants may co-exhibit Class V disease. Participants with de novo or relapsing disease may be eligible.
11. Clinically active LN at Screening requiring/receiving immunosuppression induction treatment in the opinion of the Investigator
12. Proteinuria with UPCR ≥ 1 g/g based on one 24-hour urine collection during the Screening Period

Inclusion Criteria Specific for IgA Nephropathy Cohort

13. Established diagnosis of primary Immunoglobulin A nephropathy based on kidney biopsy obtained any time prior to or during the Screening Period
14. Mean proteinuria ≥1 g/day on 2 complete and valid 24-hour urine collections during the Screening Period
15. For participants with a kidney biopsy used for eligibility >1 year prior to Screening: the presence of hematuria as defined by a positive result on urine dipstick for blood or ≥ 10 red blood cell (RBC)/hpf microscopy on urine sediment as documented by the local laboratory. The presence of hematuria documented by the central laboratory may also be acceptable.
16. Compliance with stable and optimal dose of renin-angiotensin system (RAS) inhibitor treatment including maximum allowed or tolerated angiotensin-converting enzyme (ACE) inhibitor and/or angiotensin receptor blocker (ARB) dose for ≥ 3 months prior to Screening with no expected change in dose during the study (participants with established intolerance to RAS inhibitors may be included).
17. Controlled and stable blood pressure over the past 3 months <140/90 mmHg

Exclusion Criteria:

1. Estimated glomerular filtration rate (eGFR) < 30 mL/min/1.73m^2 during Screening calculated by Chronic Kidney Disease Epidemiology Collaboration (CKD-EPI)
2. For patients with eGFR < 45 mL/min/1.73m^2 at Screening, presence of any of the following in glomeruli on most recent kidney biopsy prior to or during the Screening Period:
    - ≥ 50% interstitial fibrosis and tubular atrophy
    - ≥ 50% glomerular sclerosis
    - ≥ 50% active crescent formation
3. Concomitant significant renal disease other than LN or IgA nephropathy on the most recent biopsy prior to or during the Screening Period
4. History of kidney transplant or planned kidney transplant during the Treatment Period
5. History of other solid organ (heart, lung, small bowel, pancreas, or liver) or bone marrow transplant; or planned transplant during the Treatment Period
6. Splenectomy or functional asplenia
7. Institutionalization by administrative or court order or known medical or psychological condition(s) or risk factor that, in the opinion of the Investigator, might interfere with the participant's full participation in the study, pose any additional risk for the participant, or confound the assessment of the participant or outcome of the study
8. Known or suspected history of drug or alcohol abuse or dependence within 1 year prior to the start of the Screening Period
9. History of malignancy within 5 years of Screening with the exception of nonmelanoma skin cancer or carcinoma in situ of the cervix that has been treated with no evidence of recurrence
10. Evidence of active hepatitis B infection (positive hepatitis surface antigen [HbsAg] or positive core antibody (anti-HBc) with negative surface antibody [anti-HBs]) or active hepatitis C viral

infection (HCV antibody positive, except for patients with documented successful treatment and documented sustained virologic response [SVR]) at Screening

11. Known history of human immunodeficiency virus (HIV) infection as documented by HIV-1/HIV-2 testing or positive HIV-1/HIV-2 antibody titer at Screening
12. Bone marrow insufficiency with absolute neutrophil count < 1.3 x 10^3/µL; thrombocytopenia (platelet count < 50,000/mm^3)
13. Active systemic bacterial, viral, or fungal infection within 14 days prior to randomization
14. History of *N meningitidis* infection
15. Hypersensitivity to any ingredient contained in the study drug, including hypersensitivity to murine proteins or inability to take or tolerate the standard-of-care allowed concomitant therapies, with the exception of RAS inhibitors for the IgA nephropathy cohort
16. Received biologic, including but not limited to belimumab or rituximab, ≤ 6 months prior to Screening
17. Previously received a complement inhibitor (e.g., eculizumab) at any time
18. Participation in another investigational drug or investigational device study within 30 days before initiation of study drug on Day 1 in this study or within 5 half-lives of that investigational product, whichever is greater
19. Pregnant, breastfeeding, or intending to conceive during the course of the study

<u>Exclusion Criteria Specific for LN Cohort</u>

20. Participants who have initiated any of the following treatments for the current active LN flare:
    a. Cyclophosphamide ≤ 6 months prior to Screening
    b. Calcineurin inhibitors ≤ 3 months prior to Screening
    c. A cumulative dose of IV methylprednisolone > 3 g

d. Mycophenolate mofetil > 2 g/day (or equivalent) for ≥ 4 consecutive weeks prior to Screening

e. Oral corticosteroids ≥ 0.5 mg/kg/day for ≥ 4 consecutive weeks prior to Screening

21. Uncontrolled hypertension (systolic blood pressure > 160 or diastolic blood pressure > 110 mmHg) on 2 or more measurements during the Screening Period
22. Clinically active SLE-related cerebritis, seizures, pericarditis, stroke, or stroke syndrome requiring treatment

Exclusion Criteria Specific for IgA Nephropathy Cohort

23. Diagnosis of rapid progressive glomerulonephritis as measured by eGFR loss ≥ 30% over a period of 3 months prior to or during the Screening Period
24. Secondary etiologies of IgA nephropathy (e.g., SLE, cirrhosis, celiac disease)
25. Clinically active Henoch-Schonlein purpura (IgA vasculitis) requiring treatment
26. Prednisone or prednisone equivalent > 20 mg/day for > 14 consecutive days or any other systemic immunosuppression for the treatment of IgA nephropathy ≤ 6 months prior to Screening
27. Blood pressure of ≥ 140/90 mmHg during the Screening Period confirmed on 2 measures > 30 minutes apart
28. Body mass index ≥ 38 kg/m^2

Protocol Amendments (PA): Changes in Eligibility Criteria

In PA2 (Nov 2021), Inclusion Criterion #15 was revised to state that the presence of hematuria was required only if the biopsy used for eligibility was performed > 1 year prior to Screening, and the following was added: “The presence of hematuria documented by the central laboratory may also be acceptable.”

Inclusion Criterion #16 was revised to specify that participants with established intolerance to RAS inhibitors may be included.

Exclusion Criterion #2 was revised to specify that the criterion applies only to patients with eGFR < 45 mL/min/1.73 m^2 at Screening, that ≥ 50% of any of the listed sub-criteria are exclusionary, and “active” was added to “crescent formation.”

Institutionalization was added to Exclusion Criterion #7.

Exclusion Criterion #11 was revised from “Known history of HIV infection (evidenced by HIV type 1 or type 2 [HIV 1, HIV 2] antibody)” to “Known history of human immunodeficiency virus (HIV) infection as documented by HIV-1/HIV-2 testing or positive HIV-1/HIV-2 antibody titer at Screening.”

“Hypersensitivity to any ingredient contained in the study drug, including hypersensitivity to murine proteins” was added to Exclusion Criterion #15.

“With the exception of RAS inhibitors for the IgA nephropathy cohort” was added to Exclusion Criterion #15.

Exclusion Criterion #26 was revised to specify > 20 mg “per day” for prednisone or prednisone equivalent. The criterion was also revised to specify other types of immunosuppression as systemic and that the specified exclusion is for treatments of IgA nephropathy.

Exclusion Criterion #28 was revised to “BMI ≥ 38 kg/m^2.”

In PA4 (Mar 2024), Exclusion Criterion #10 was revised from “Known history of hepatitis B or C viral infection” to “Evidence of active hepatitis B infection (positive hepatitis surface antigen [HbsAg] or positive core antibody (anti-HBc) with negative surface antibody [anti-HBs]) or active hepatitis C viral infection (HCV antibody positive, except for patients with documented successful treatment and documented sustained virologic response [SVR]) at Screening.”

Study Assessments: Additional Detail

Participants provided two separate complete and valid 24-hour urine collections during the screening period, at week 26 (to assess the primary endpoint), and at week 50 (to assess a secondary endpoint). The two valid 24-hour urine collections should have been obtained within 2 weeks before the week 26 and week 50 visits. When two 24-hour urine collections were required and only one collection was available at the visit, then this value was used for analysis. When more than two collections were available at the visit, only two valid collections recorded in the electronic case report form were used for analysis. Completeness of the 24-hour urine collection was estimated from rate of creatinine excretion. Normal values of creatinine excretion vary with age and body weight. Hence, a 24-hour urine collection is considered valid if all the following criteria are met, otherwise the urine collection is required to be repeated:

- The collection is between 22 and 26 hours in duration (i.e., time from the initial discarded void to the last void/attempt to void)

- No voids are missed between the start and end time of the collection as indicated by the participant's urine collection diary
- The 24-hour creatinine content is within 30% of the expected range as estimated by the following formula: [(140-age) × weight]/5000, where weight is in kilograms. This result is multiplied by 0.85 in women
- The maximum variation in total 24-hour urine creatinine between the two urine collections must be ≤ 30%

Urine collections that deviate from the above criteria were reviewed by the Medical Monitoring team and may be considered acceptable if determined to be not clinically significant. If any of the collections do not meet the validity criteria outlined above, the collection must be repeated as soon as possible within the time frames outlined in the Schedule of Assessments in order to ensure that two valid collections are obtained for each of the study time points. Morning spot urine samples were collected at all study visits according to the Schedule of Assessments.

Change in kidney function was monitored using measurements of eGFR (mL/min/1.73m^2) and creatinine clearance on 24-hour urine collection. The eGFR calculation was based on the CKD-EPI formula for all participants using serum creatinine collected prior to study drug administration, if applicable.

Physical examination, vital signs and pulse oximetry, electrocardiograms, labs, and pregnancy tests in women of childbearing potential were conducted at the frequency specified in the Schedule of Assessments. Monitoring for adverse events (AEs) was continuous throughout the study.

Study Endpoints: Additional Detail

Secondary Endpoints

Secondary endpoints were based on the Full Analysis Set. These analyses were descriptive in nature, and no adjustment for multiplicity was performed.

- Percentage change in proteinuria from baseline to week 50 (based on 24-hour urine collection[s] at each time point)
    - The percentage change from baseline in proteinuria at week 50 was analyzed using a mixed-effect model for repeated measures (MMRM) using all available longitudinal data (either complete or partial). Data collected on or after treatment discontinuation were assumed to be missing at random and imputed using multiple imputation. The model included change from baseline in log-transformed proteinuria as the response variable and fixed, categorical effects of treatment group, randomization stratification factor, visit, and treatment group by visit interaction as well as a fixed, continuous effect of baseline log proteinuria as a covariate. An unstructured covariance matrix was used to model the correlations among repeated measurements for each participant. If this analysis failed to converge, a first-order autoregressive covariance matrix was used. The Kenward–Roger approximation was used to estimate denominator degrees of freedom. As patients on placebo switched to ravulizumab at week 26, the percentage change from baseline in proteinuria at week 50 was summarized descriptively for each treatment group and for each visit from the MMRM analysis, and no formal treatment comparison was conducted
- Percentages of participants with >30% and >50% reduction in proteinuria at week 26 and week 50 compared with baseline (based on 24-hour urine collection[s] at each time point)

    - The percentage of participants with >30% and >50% reduction in proteinuria were summarized at week 26 and week 50 by treatment group by calculating the point estimate and two-sided 95% confidence interval (CI), based on exact confidence limits using the Clopper–Pearson method. If a participant discontinued treatment, they were to be considered a nonresponder from the point of treatment discontinuation onward
- Change from baseline in eGFR at week 26 and week 50
    - Kidney function evaluated by eGFR was analyzed using the same MMRM method specified as above
    - If a participant required dialysis for acute kidney injury, a value of 10 mL/min/1.73 m^2 was to be imputed for while the participant was on dialysis (i.e., from the first day of dialysis through 5 days after the end of dialysis)
    - Data collected on or after treatment discontinuation were to be assumed missing and handled in the MMRM as missing at random
- Percentage of participants meeting the criteria for partial remission at week 26 and week 50 (defined as mean proteinuria <1 g/24hrs based on two valid 24-hour urine collections obtained within 2 weeks prior to the study visit [week 26 or week 50])
    - The percentage of participants meeting the criteria for partial remission was summarized at week 26 and week 50 by treatment group by calculating the point estimate and two-sided 95% CI, based on exact confidence limits using the Clopper–Pearson method. If a participant discontinued treatment, they were to be considered a nonresponder from the point of treatment discontinuation onward
- Absolute values and change from baseline in serum C3 and C4 concentrations at week 26 and week 50
    - Serum C3 and C4 concentrations were summarized at baseline and each postbaseline time point by treatment group using descriptive statistics for the

observed value as well as the change from baseline. There was no imputation for missing data or data collected on or after treatment discontinuation

Exploratory endpoints were based on the Full Analysis Set. These analyses were descriptive in nature, and no adjustment for multiplicity was performed:

- Slope of eGFR computed from baseline to week 26 and week 50
    - The slope of eGFR was computed using a mixed-effect model including data through week 26 or week 50. The model included eGFR as the response variable, random patient effects for intercepts and slopes, fixed categorical effects of treatment group, randomization stratification factor, and treatment group by visit interaction as well as a fixed, continuous effect of visit (study year)

Other prespecified proteinuria assessments

In addition to the proteinuria analyses based on 24-hour urine collections, the percentage change from baseline in spot urine protein-to-creatinine ratio (UPCR) at each scheduled visit was analyzed using an MMRM. The primary efficacy endpoint was also analyzed by the following subgroups (mean and CI for percentage change in proteinuria at week 26):

- Age at screening (18 to 65 years, >65 years)
- Gender (female, male)
- Baseline eGFR (30 to 60, >60 to 90, >90 mL/min/1.73m^2)
- Duration of disease (≤1 year, 1 to 5 years, >5 years)
- Race (Asian or Other Pacific Islander, non-Asian or Other Pacific Islander)
- Baseline proteinuria (1 to 2 g/day, >2 g/day)

Safety data were analyzed for the Safety Set, which includes all participants who received ≥1 dose of study intervention. Participants were analyzed according to the study intervention they actually received. AEs were coded in MedDRA version 23.0 or higher and summarized by MedDRA system

organ class (SOC) and preferred term (PT) overall, by severity (toxicity grade), and by relationship to treatment. Participants having multiple AEs within a category (e.g., overall, SOC, PT) were counted once in that category. For severity/relationship tables, the participant's highest grade/most related event within a category was counted. Treatment-emergent AEs (TEAEs) and SAEs were summarized descriptively by treatment group. Percentages were based on the number of treated participants in the Safety Set within a treatment group. safety data included deaths, AEs leading to study treatment discontinuation, AEs leading to withdrawal from the study, and AEs of special interest (meningococcal infections).

Immunogenicity analyses were performed on the Safety Set. The incidence of anti-drug antibodies (ADAs) to ravulizumab were summarized descriptively and separately for each disease cohort by treatment group at baseline and each postbaseline time point. Participants who were ADA positive were further categorized by pre-existing immunogenicity or treatment-emergent ADA response. In addition, ADA-positive samples were further characterized for neutralizing activity in the Nab assay.

Supplemental Results – Immunogenicity

Anti-drug antibody (ADA) response

In the ravulizumab arm, five of 43 (12%) showed pre-existing immunoreactivity and one of 43 (2%) exhibited treatment-boosted ADA response which was persistent. One of 43 (2%) exhibited treatment-emergent ADA response, which was transient. None of these patients had neutralizing antibodies, and the ADA responses had no apparent impact on pharmacokinetics, pharmacodynamics, efficacy, or safety. In the placebo arm during the placebo treatment period, one of 23 (4%) showed pre-existing immunoreactivity, and three of 23 (13%) exhibited treatment-emergent ADA responses, of which one was transient and two were indeterminate. These responses were then negative during the open-label ravulizumab treatment period and no additional ADA-positive responses were observed during this period.

Supplemental Figure 1. Study design.

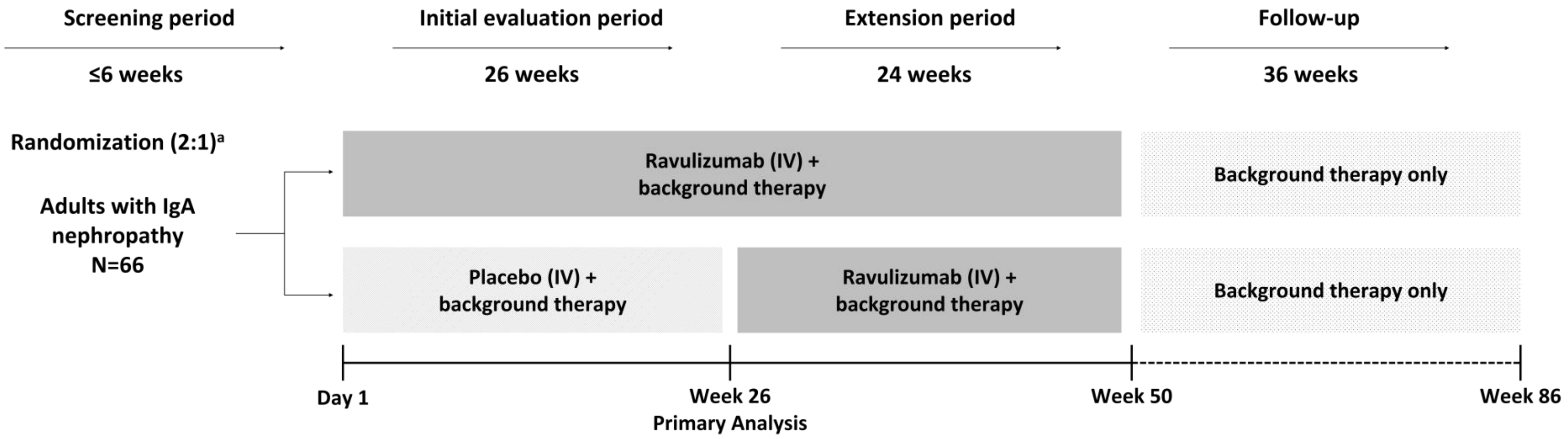

IgA, immunoglobulin A; IV, intravenous.

[a]Randomization was stratified by mean proteinuria (1.0–2.0 g/day versus >2.0 g/day) based on two valid 24-hour urine collections during screening.

Supplemental Figure 2. Percentage of patients with >30% and >50% reduction in proteinuria

A) Percentage of patients with >30% and >50% proteinuria reduction based on 24-hour urine protein (g/day) at week 26 (secondary endpoint)

B) Percentage of patients with >30% and >50% proteinuria reduction based on 24-hour urine protein (g/day) at week 50 (secondary endpoint)

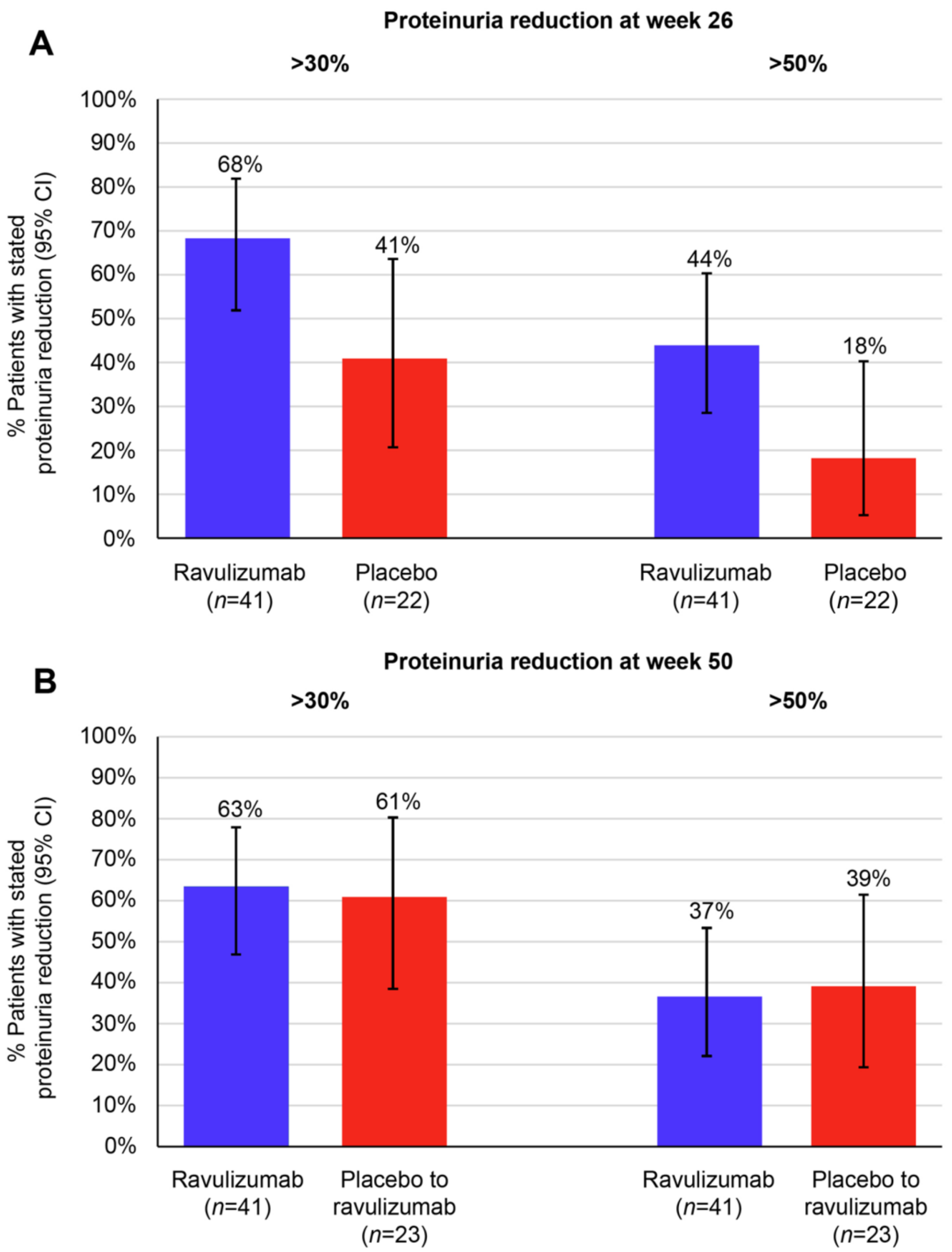

95% confidence intervals for the proportion are based on Cloppers–Pearson method.

CI, confidence interval

Supplemental Figure 3. Percentage of patients meeting the criteria for partial remission (<1 g/day) based on 24-hour urine protein at week 26 and week 50 (secondary endpoints).

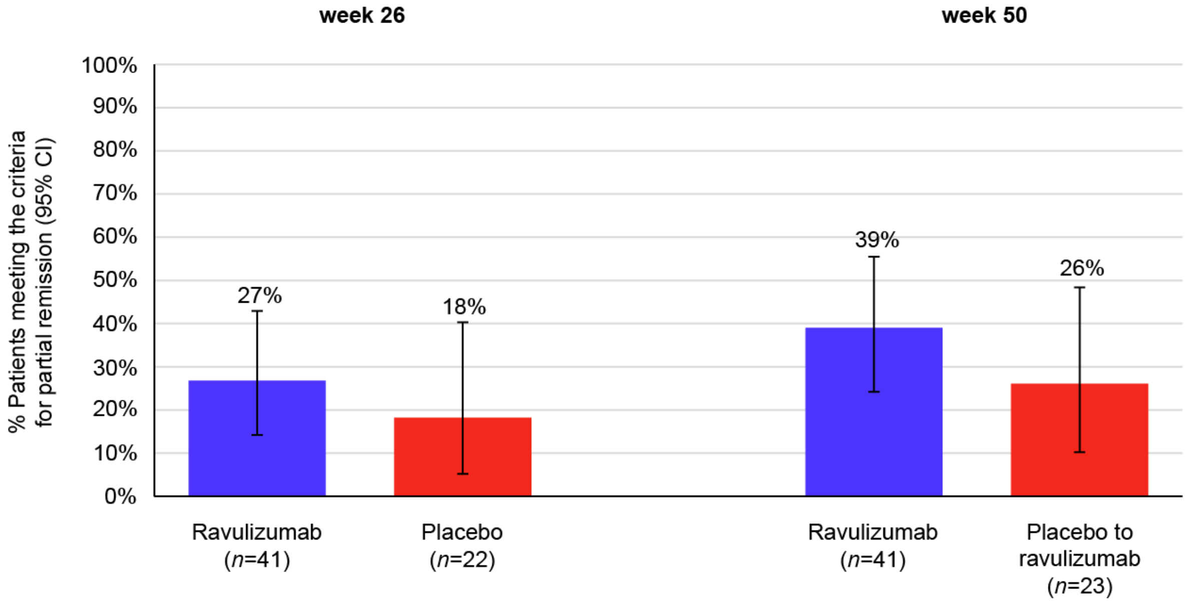

95% confidence intervals for the proportion are based on Clopper–Pearson method.

CI, confidence interval

Supplemental Figure 4. A statistically significant reduction in proteinuria with ravulizumab versus placebo was seen across several subgroups of patients: Difference in change in proteinuria (and 90% confidence interval) from baseline to week 26, ravulizumab versus placebo in subgroups of patients (based on 24-hour urine protein) (exploratory endpoint).

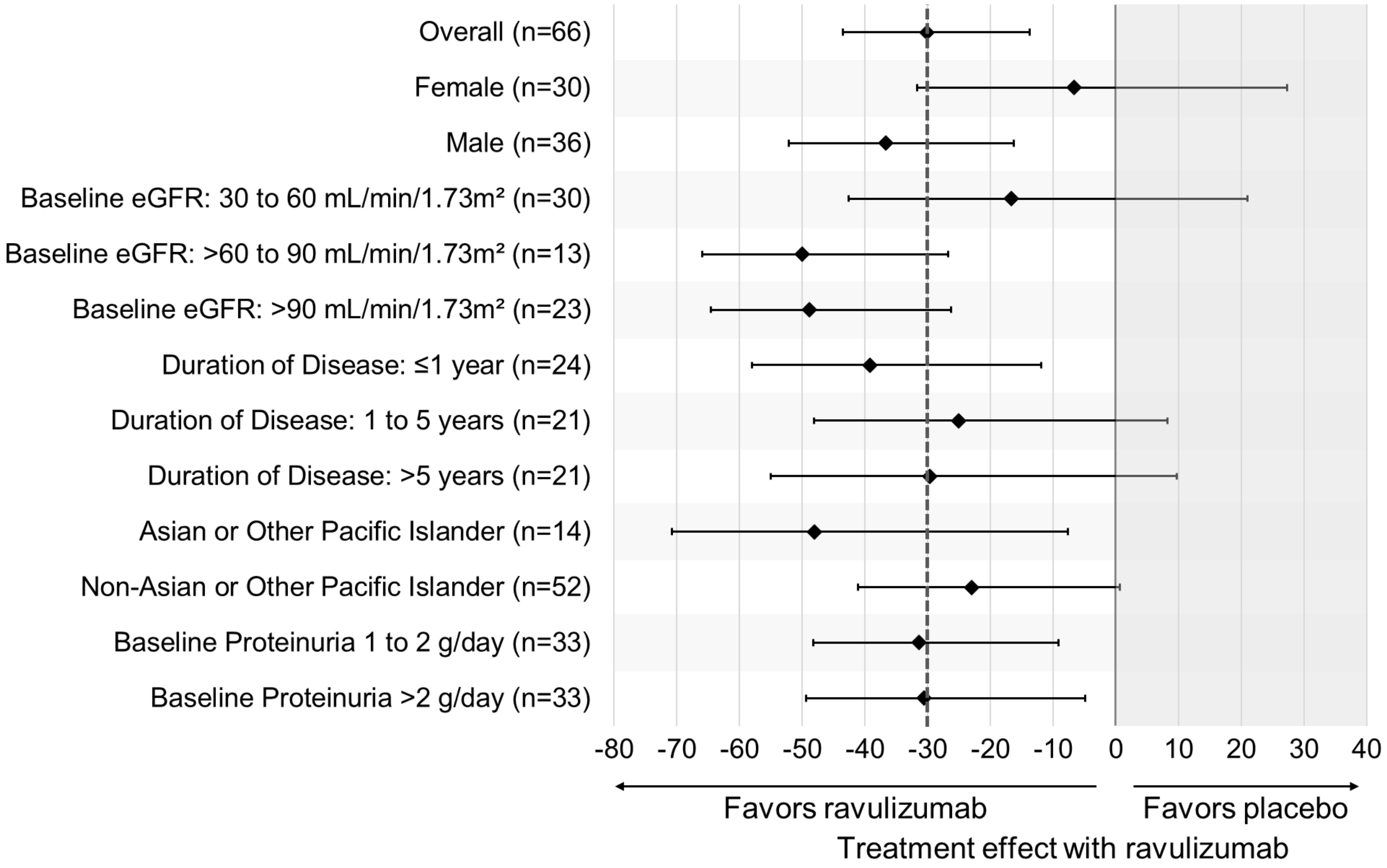

Reference line: −30.1% (90% CI −43.5% to −13.7%; P = 0.0053), primary analysis treatment effect.

CI, confidence interval, eGFR, estimated glomerular filtration rate

Supplemental Figure 5. Percentage of participants with absence of hematuria: ≤5 red blood cells/high-power field on urine sediment from spot samples reported by the central lab, from baseline to week 50 (*post hoc* analysis)

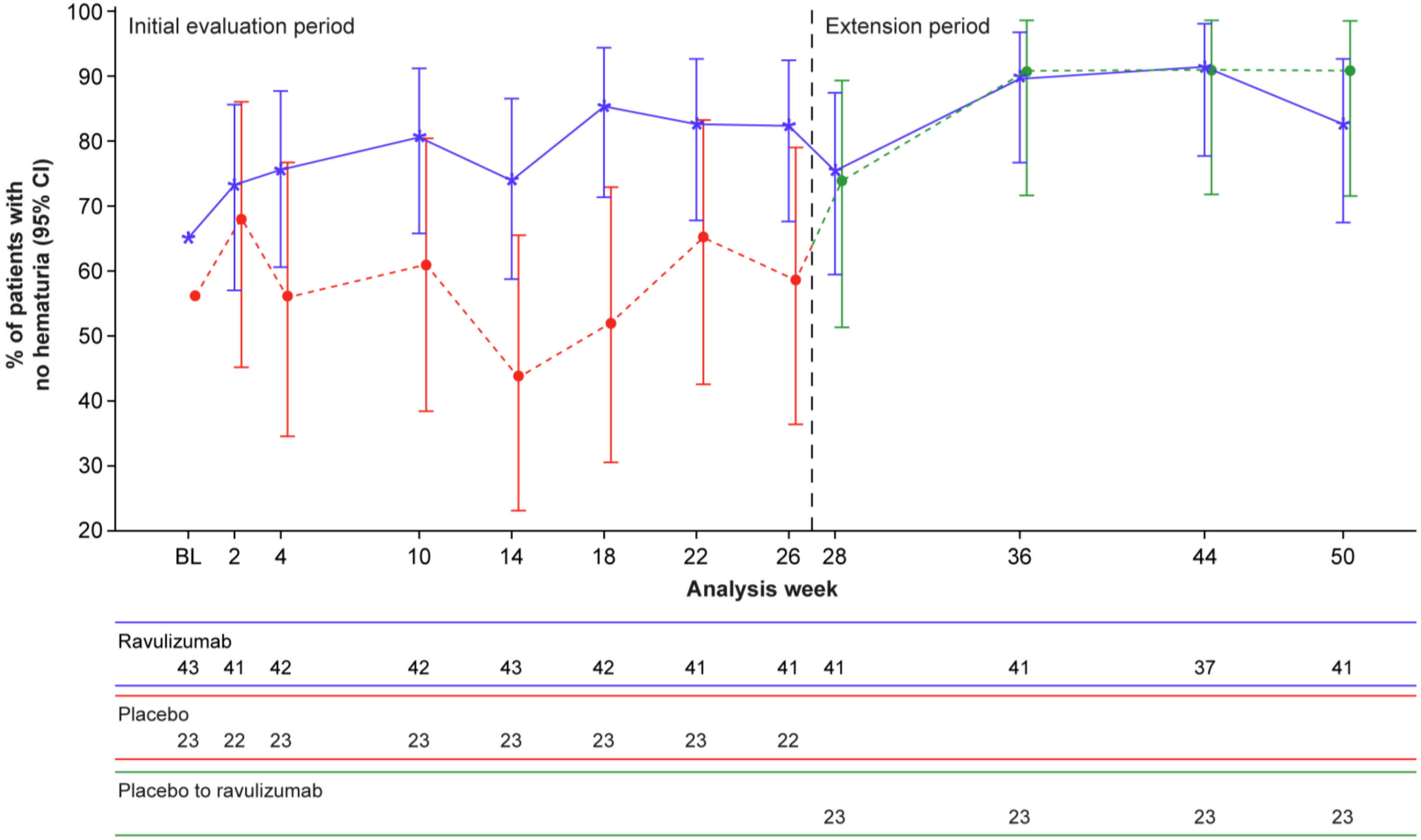

Dotted line indicates crossover from placebo to ravulizumab.

BL, baseline; CI, confidence interval; hpf, high-power field; RBCs, red blood cells

Supplemental Figure 6. Pharmacokinetics: mean (standard deviation) ravulizumab concentration over time by treatment group

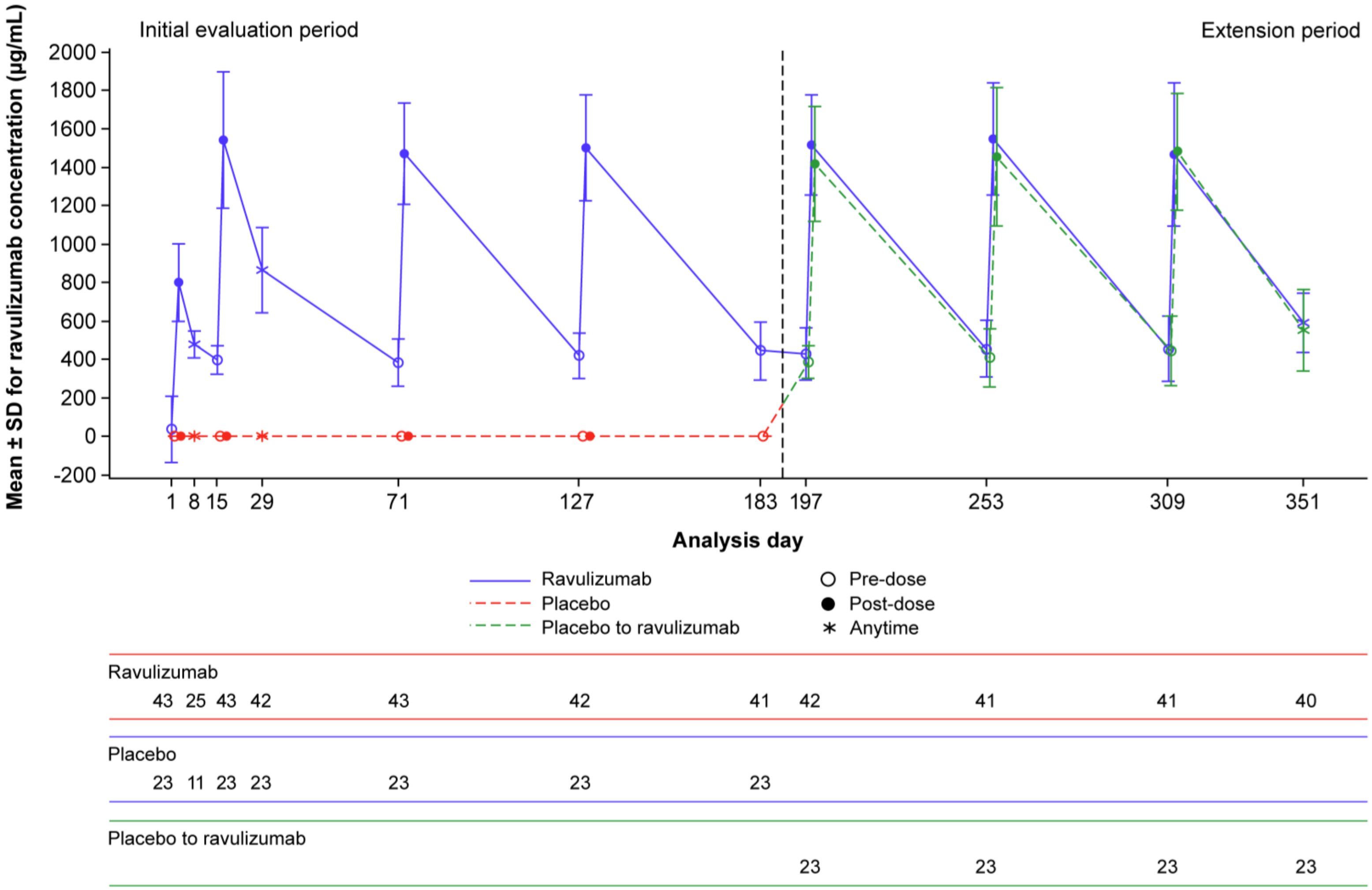

Dotted line indicates crossover from placebo to ravulizumab.

Supplemental Figure 7. Pharmacodynamics: serum free C5 concentration over time by treatment group

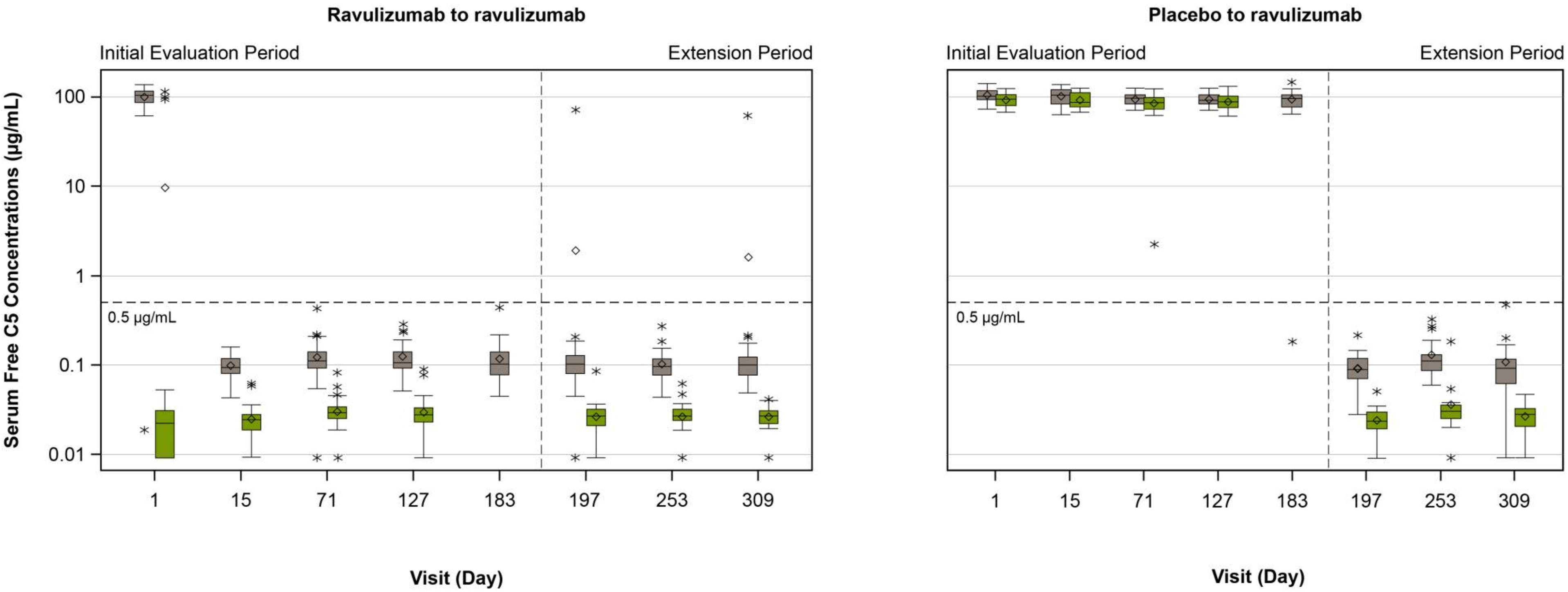

	1	15	71	127	183	197	253	309
Pre-Dose n	43	43	41	39	39	40	38	41
Post-Dose n	43	42	41	41	NA	40	38	41

	1	15	71	127	183	197	253	309
Pre-Dose n	22	23	21	23	23	22	21	21
Post-Dose n	22	22	21	22	NA	20	22	23

Pre-Dose   Post-Dose

* represents assessments outside of the box plot whiskers

Supplemental Table 1. Weight-based dosing of ravulizumab

Body Weight Range (kg)[a]	Ravulizumab Loading Dose (mg)	Ravulizumab Maintenance Dose (mg)
≥ 40 to < 60	2400	3000
≥ 60 to < 100	2700	3900
≥ 100	3000	5400

[a]Dose regimen was based on the last recorded study visit body weight. If the study drug was prepared the night before a visit, the weight from the most recent study visit was used.

Supplemental Table 2. Baseline biopsy characteristics and MEST-C scores in ravulizumab and placebo groups

Baseline characteristic	Ravulizumab (*n* = 43)	Placebo (*n* = 23)	Total (*N* = 66)
Time from eligibility biopsy to randomization			
< 1 month	0 (0)	0 (0)	0 (0)
1 to ≤3 months	5 (12)	4 (17)	9 (14)
3 to ≤ 6 months	4 (9)	5 (22)	9 (14)
6 to ≤12 months	8 (19)	4 (17)	12 (18)
12 to ≤24 months	5 (12)	2 (9)	7 (11)
> 24 months	21 (49)	8 (35)	29 (44)
Baseline MEST-C score based on local pathology			
M-Score			
M0	14 (33)	7 (30)	21 (32)
M1	23 (54)	13 (57)	36 (55)
Not available	6 (14)	3 (13)	9 (14)
E-Score			
E0	19 (44)	14 (61)	33 (50)
E1	17 (40)	6 (26)	23 (35)
Not available	7 (16)	3 (13)	10 (15)
S-Score			
S0	5 (12)	2 (9)	7 (11)
S1	32 (74)	18 (78)	50 (76)
Not available	6 (14)	3 (13)	9 (14)
T-Score			
T0	22 (51)	11 (48)	33 (50)
T1	14 (33)	8 (35)	22 (33)
T2	0 (0)	1 (4)	1 (2)

Not available	6 (14)	3 (13)	9 (14)
Missing	1 (2)	0 (0)	1 (2)
C-Score			
C0	15 (35)	14 (61)	29 (44)
C1	11 (26)	6 (26)	17 (26)
C2	1 (2)	0 (0)	1 (2)
Not available	15 (35)	3 (13)	18 (27)
Missing	1 (2)	0 (0)	1 (2)

Supplemental Table 3. Baseline complement staining in ravulizumab and placebo groups

Baseline characteristic	Ravulizumab *n* (%), (*n* = 43)	Placebo *n* (%), (*n* = 23)	Total *N* (%), (*N* = 66)
Glomerular C3 staining based on local pathology			
0+	4 (9)	3 (13)	7 (11)
1+	14 (33)	3 (13)	17 (26)
2+	9 (21)	12 (52)	21 (32)
3+	10 (23)	3 (13)	13 (20)
Not applicable	5 (12)	2 (9)	7 (11)
Missing	1 (2)	0 (0)	1 (2)
Glomerular C4d staining based on local pathology			
0+	9 (21)	5 (22)	14 (21)
1+	2 (5)	2 (9)	4 (6)
2+	0 (0)	0 (0)	0 (0)
3+	0 (0)	0 (0)	0 (0)
Not applicable	31 (72)	16 (70)	47 (71)
Missing	1 (2)	0 (0)	1 (2)
Glomerular C1q staining based on local pathology			
0+	19 (44)	12 (52)	31 (47)
1+	5 (12)	3 (13)	8 (12)
2+	1 (2)	0 (0)	1 (2)
3+	0 (0)	0 (0)	0 (0)
Not applicable	17 (40)	8 (35)	25 (38)
Missing	1 (2)	0 (0)	1 (2)

MEST-C, mesangial hypercellularity (M), endocapillary hypercellularity (E), segmental glomerulosclerosis (S), tubular atrophy/interstitial fibrosis (T), crescents (C).

Supplemental Table 4. Change from baseline in eGFR over time, by treatment group (secondary endpoint)

Timepoint	LSM change in eGFR, mL/min per 1.73m^2 (95% CI)	
	**Ravulizumab (*n* = 43)**	**Placebo to Week 26; Change to Ravulizumab Week 26 to 50 (*n* = 23)**
Week 2	−0.20 (−2.61 to 2.21)	−1.24 (−4.47 to 1.99)
Week 4	0.65 (−1.50 to 2.80)	−4.11 (−7.01 to −1.21)
Week 10	1.33 (−1.03 to 3.68)	−2.24 (−5.45 to 0.97)
Week 14	0.73 (−2.10 to 3.56)	−3.98 (−7.81 to −0.16)
Week 18	−0.51 (−2.95 to 1.92)	−4.50 (−7.82 to −1.18)
Week 22	−1.41 (−4.32 to 1.49)	−4.24 (−8.13 to −0.35)
Week 26	0.21 (−2.30 to 2.73)	−4.50 (−7.90 to −1.11)
Week 28	0.00 (−2.76 to 2.75)	−2.89 (−6.63 to 0.84)
Week 36	0.20 (−2.62 to 3.02)	−1.07 (−4.88 to 2.74)
Week 44	0.04 (−2.39 to 2.47)	−7.24 (−10.43 to −4.06)
Week 50	−3.87 (−6.41 to −1.33)	−6.33 (−9.72 to −2.94)

eGFR calculation is based on the CKD-EPI formula.

CI, confidence interval; CKD-EPI, Chronic Kidney Disease Epidemiology Collaboration; eGFR, estimated glomerular filtration rate; LSM, least-squares mean.

Supplemental Table 5. Percentage change from baseline in 24-hour urine albumin-to-creatinine ratio (g/mol) (exploratory endpoint)

UACR over time	Ravulizumab (*n = 43*)	Placebo (*n = 23*)
**Baseline**		
**UACR g/mol, mean (SD)**	132.7 (66.5)	144.8 (80.3)
**Week 26[a]**		
**UACR g/mol, mean (SD)**	83.7 (51.1)	133.3 (95.5)
**% change from baseline, mean (SD)**	−36.8 (27.3)	3.6 (59.2)
**Week 50[b]**		
**UACR g/mol, mean (SD)**	84.1 (54.9)	90.3 (62.1)
**% change from baseline, mean (SD)**	−31.5 (42.0)	−32.7 (35.7)

[a]At week 26, n=41 for ravulizumab group and n=22 for placebo group

[b]At week 50, n=41 for ravulizumab group and n=23 for placebo group

Supplemental Table 6. Change from baseline to week 26 in serum C3 and C4 (secondary endpoint)

Parameter	Ravulizumab (*n* = 43)	Placebo (*n* = 23)
Mean change in serum C3, g/L (SD)	+0.027 (0.2389)	−0.019 (0.2366)
Mean change in serum C4, g/L (SD)	−0.005 (0.0576)	−0.001 (0.0566)

At baseline, two patients in the ravulizumab group and one patient in the placebo group had low serum C3, and no patients had low serum C4 (normal range for serum C3: 0.9–1.8 g/L; normal range for serum C4: 0.1–0.4 g/L).

C3, complement component 3; C4, complement component 4; SD, standard deviation.

Plain Language Summary

Efficacy and Safety of Ravulizumab in IgA Nephropathy: Results of a Phase 2 Randomized Double-Blind Placebo-Controlled Trial: *Plain Language Summary*

Richard Lafayette,[1] James Tumlin,[2] Roberta Fenoglio,[3] Jessica Kaufeld,[4] Miguel Angel Pérez Valdivia,[5] Mai-Szu Wu,[6] Shih-Han Susan Huang,[7] Eric Alamartine,[8] Sung Gyun Kim,[9] Min Yee,[10] Andreas Kateifides,[10] Kara Rice,[10] Katherine Garlo,[10] Jonathan Barratt,[11] and the SANCTUARY study investigators

[1]Stanford Glomerular Disease Center, Stanford University Medical Center, Stanford, CA, USA; [2]Department of Medicine, Division of Renal Medicine, Emory University, Atlanta, Georgia, USA; [3]University Center of Excellence on Nephrological, Rheumatological and Rare Diseases including Nephrology and Dialysis Unit and Center of Immuno-Rheumatology and Rare Diseases, Coordinating Center of the Inter-regional Network for Rare Diseases of Piedmont and Aosta Valley (North-West Italy), San Giovanni Bosco Hub Hospital, ASL Città di Torino, and Department of Clinical and Biological Sciences of the University of Turin, Turin, Italy; [4]Medizinische Hochschule Hannover, Hannover, Germany; [5]Servicio de Nefrología, UGC Urología-Nefrología, Hospital Virgen del Rocío, Seville, Spain; [6]Division of Nephrology, Shuang Ho Hospital, Taipei Medical University, Taipei, Taiwan; [7]Kidney Clinical Research Unit, London Health Sciences Center, East London, Ontario, Canada; [8]Service de Néphrologie, Hôpital Nord CHU Saint Etienne, Saint Etienne, France; [9]Department of Internal Medicine, Hallym University Sacred Heart Hospital, Anyang, Korea; [10]Alexion, AstraZeneca Rare Disease, Boston, MA, USA; [11]Department of Cardiovascular Sciences, University of Leicester, Leicester, UK.

INTRODUCTION TO IgA NEPHROPATHY

- **Immunoglobulin A (IgA) nephropathy** is an autoimmune disease that may lead to chronic kidney disease and kidney failure
- Measuring the amount of protein in the urine (proteinuria) is a method to assess disease severity
- Physicians also monitor kidney function, using the estimated glomerular filtration rate (eGFR)
- The **complement system**, a part of the immune system, plays a key role in IgA nephropathy

WHY WAS THIS STUDY NEEDED?

- Researchers wanted to test whether a drug called **ravulizumab** could be a potential treatment for IgA nephropathy
- Ravulizumab is already used by physicians to treat several other conditions
- It works by blocking activation of complement component 5 (C5), which prevents formation of C5a and C5b-9, proteins that cause inflammation and cell damage

HOW WAS THIS STUDY DONE?

- This was a randomized placebo-controlled trial – eligible participants received either ravulizumab or placebo intravenously every 8 weeks for 26 weeks, at which point all were then treated with ravulizumab for the next 24 weeks
- Throughout the clinical trial, patients remained on an angiotensin-converting enzyme inhibitor (ACE-inhibitor) or angiotensin receptor blocker (ARB)

Participants were assigned randomly to ravulizumab or placebo, in a 2:1 ratio. No one involved in the study knew who received ravulizumab or placebo

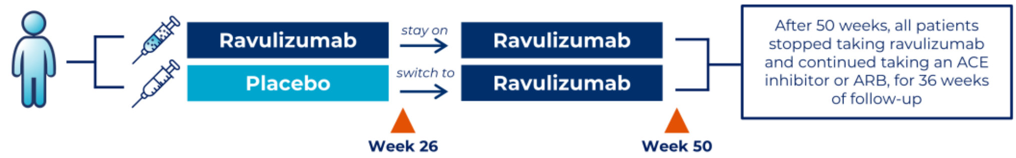

KEY INCLUSION CRITERIA

Key eligibility criteria (does not include all):

- Biopsy-proven IgA nephropathy
- At least 1 gram of proteinuria per day
- eGFR ≥30 mL/min/1.73m²
- On a stable and optimal dose of an ACE-inhibitor or ARB
- Vaccination against meningococcal infection was required

WHAT WAS MEASURED?

- Data were collected and analyzed after patients completed 26 weeks of treatment and after 50 weeks of treatment
- The **primary endpoint** – the main outcome measure to assess how well the drug worked (efficacy) – was the percentage change in proteinuria from baseline (start of study) to Week 26
- Researchers also evaluated **safety**

WHAT DID THIS STUDY FIND?

43 participants were in the ravulizumab group and 23 were in the placebo group

Primary endpoint, at week 26: Participants who received ravulizumab had a 41.9% reduction in proteinuria and those who received placebo had a 16.8% reduction in proteinuria. This difference was statistically significant, meaning that ravulizumab performed better than placebo.

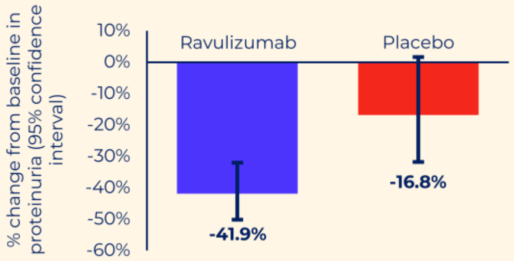

At **week 50**:

- Participants who had been on ravulizumab had a 41.1% reduction in proteinuria from baseline
- Those who were on placebo for the first 26 weeks and switched to ravulizumab for the next 24 weeks had a 43.1% reduction in proteinuria from week 0 to week 50

Safety data were similar with ravulizumab and placebo

- Investigators assessed the majority of adverse events as mild and not related to the study treatment (ravulizumab or placebo)
- There were no serious adverse events that were considered related to study treatment, and no participants had to withdraw from the study due to an adverse event
- No meningococcal infections or deaths occurred

CONCLUSIONS

- The researchers concluded that treatment with ravulizumab led to improved outcomes versus placebo in terms of the reduction of proteinuria
- These positive results suggest that we can proceed with testing ravulizumab in a larger study, with more patients and sites globally (phase 3 trial)
- This phase 3 trial is currently enrolling patients

GLOSSARY

ACE-inhibitor: inhibits angiotensin-converting enzyme and the production of angiotensin 2, leading to less narrowing of blood vessels and lower blood pressure
ARB: blocks the action of angiotensin 2 on receptors, leading to less narrowing of blood vessels and lower blood pressure
Adverse event: any abnormal clinical events or findings that occur while taking a drug, regardless of whether they are related to the drug
Placebo: identical in appearance but does not contain active drug

Abbreviations ACE, angiotensin-converting enzyme; ARB, angiotensin receptor blocker; C5, complement component 5; CI, confidence interval; eGFR, estimated glomerular filtration rate.

TITLE PAGE

STATISTICAL ANALYSIS PLAN

Version Number: 3.0

Protocol Title: A Phase 2, Double-Blind, Randomized, Placebo-Controlled Study to Evaluate the Efficacy and Safety of Ravulizumab in Adult Participants With Proliferative Lupus Nephritis (LN) or Immunoglobulin A Nephropathy (IgAN)

Protocol Number: ALXN1210-NEPH-202

Protocol Amendment Number: Amendment 4 (dated 26 Mar 2024)

Compound: Ravulizumab (ALXN1210)

Short Title: Phase 2 Study of Ravulizumab in Proliferative Lupus Nephritis (LN) or Immunoglobulin A Nephropathy (IgAN)

Sponsor Name: Alexion Pharmaceuticals, Inc.

Legal Registered Address:

121 Seaport Boulevard

Boston, MA 02210

Author: PPD

Version Date: 31 Jul 2024

TABLE OF CONTENTS

TITLE PAGE 1
VERSION HISTORY 6
APPROVAL SIGNATURES 7
1. INTRODUCTION 8
1.1. Objectives and Endpoints 8
1.2. Study Design 10
2. STATISTICAL HYPOTHESES 13
3. SAMPLE SIZE DETERMINATION 14
4. ANALYSIS SETS 15
5. STATISTICAL ANALYSES 17
5.1. General Considerations 17
5.1.1. Data Presentation for the Initial Evaluation Period 17
5.1.2. Data Presentation for the Extension Period 17
5.1.3. Data Presentation for the Post-treatment Follow-up Period 17
5.1.4. Handling of Dropouts or Missing Data 18
5.2. Study Participants 21
5.3. Primary Endpoint Analysis 21
5.3.1. Percentage Change from Baseline in Proteinuria 21
5.3.2. Main Analytical Approach 22
5.3.3. Sensitivity Analysis 22
5.3.4. Supplementary Analysis 23
5.4. Secondary Endpoint Analysis 25
5.4.1. Proteinuria 25
5.4.2. Estimated Glomerular Filtration Rate 26
5.4.3. Serum C3 and C4 Concentrations 27
5.4.4. Complete Renal Response (LN Cohort Only) 27
5.4.5. Partial Renal Response (LN Cohort Only) 28
5.4.6. Time to UPCR < 0.5 g/g (LN Cohort Only) 28
5.4.7. Corticosteroid Taper (LN Cohort Only) 28
5.4.8. Renal Flare (LN Cohort Only) 29
5.4.9. Extrarenal SLE Flare (LN Cohort Only) 29

5.4.10. Suboptimal Response (LN Cohort Only) 30
5.4.11. Treatment Failure (LN Cohort Only) 30
5.4.12. Serum Albumin (LN Cohort Only) 30
5.4.13. Partial Remission (IgAN Cohort Only) 30
5.5. Tertiary/Exploratory Endpoint Analysis....31
5.5.1. Effect on Hematuria....31
5.5.2. SF-36v2 31
5.5.3. EQ-5D-5L 32
5.5.4. Exploratory Biomarkers 32
5.5.5. Time to CRR and PRR (LN Cohort Only) 33
5.5.6. Overall Renal Response (LN Cohort Only)....33
5.5.7. Time to UPCR > 50% Decrease (LN Cohort Only) 33
5.5.8. FACIT-Fatigue (LN Cohort Only)....33
5.5.9. Anti-dsDNA and Anti-C1q antibodies (LN Cohort Only)....34
5.5.10. Histology (LN Cohort Only) 34
5.5.11. Slope of eGFR (IgAN Cohort Only) 34
5.6. Safety Analyses 34
5.6.1. Extent of Exposure 35
5.6.2. Adverse Events....35
5.6.2.1. Overall Summary of AEs 36
5.6.2.2. AEs and SAEs by SOC and PT 36
5.6.2.3. AEs and SAEs by SOC, PT, and Relationship....36
5.6.2.4. AEs and SAEs by SOC, PT, and Severity 36
5.6.2.5. Deaths and Other Significant AEs 36
5.6.3. Additional Safety Assessments....36
5.6.3.1. Laboratory Parameters 36
5.6.3.2. Vital Signs 37
5.6.3.3. Electrocardiogram (ECG)....37
5.6.3.4. Physical Exam....37
5.7. Other Analyses 38
5.7.1. Pharmacokinetic and Pharmacodynamic Analyses 38
5.7.2. Immunogenicity Analyses....38
5.7.3. COVID-Related Analyses 39

5.7.4. Subgroup Analyses 40
5.8. Interim Analyses 42
5.8.1. Dose Confirmation Analysis 42
5.8.2. Early IgAN Interim Analysis 42
5.8.3. Early LN Interim Analysis 42
5.8.4. Week 26 Analysis 43
5.8.5. Week 50 Analysis 43
6. SUPPORTING DOCUMENTATION 44
6.1. Appendix 1: Technical Specifications for Derived Variables 44
6.1.1. Disease Duration 44
6.1.2. Definition of Baseline Values 44
6.1.3. Change from Baseline 44
6.1.4. Percent Change from Baseline 44
6.1.5. Analysis Visits 44
6.1.6. Analysis Value 45
6.1.7. AEs 45
6.1.8. AEs Related to Meningococcal Infection 45
6.1.9. CRR (LN Cohort Only) 46
6.1.10. Concomitant Medications/Therapies 46
6.1.11. Partial Remission (IgAN cohort only) 47
6.1.12. PRR (LN cohort only) 47
6.1.13. Serum Creatinine and eGFR 48
6.2. Appendix 2: Study and Participant Characteristics 48
6.2.1. Demographics, Disease Characteristics, and History 48
6.2.1.1. Demographics 48
6.2.1.2. Disease Characteristics 49
6.2.1.3. Medical/Surgical History and Baseline Physical Examination 51
6.2.2. Prior and Concomitant Medications / Therapies 51
6.2.3. Allowed Concomitant Therapy and Additional Standard-of-Care Therapy 52
6.2.4. Protocol Deviations 52
6.3. Appendix 3: Instrument Scoring Details 53
6.3.1. SF-36v2 53
6.3.1.1. Data Cleaning and Item Recoding 53

6.3.1.2. Item Recalibration....................53
6.3.1.3. Computation of Raw Scores....................53
6.3.1.4. Transformation of Raw Scale Score to 0 to 100 Scores....................53
6.3.1.5. Transformation of 0 to 100 Scores to T-score Based Scores....................54
6.3.1.6. Scoring the SF-36v2 Health Survey Component Summary Measures....................54
6.3.1.7. Handling of Missing Items....................55
6.3.2. EQ-5D-5L....................55
6.3.3. FACIT-Fatigue....................56
6.4. Appendix 4: Additional Details on Statistical Methods....................56
6.4.1. SAS Code for ANCOVA Analysis....................56
6.4.2. SAS Code for MMRM Analysis....................57
6.4.3. SAS Code for Multiple Imputation....................57
6.4.4. SAS Code for Placebo-based Imputation....................58
6.4.5. SAS Code for Tipping Point Sensitivity Analysis....................58
6.5. Appendix 5: Changes to Protocol-planned Analyses....................59
6.6. Appendix 6: List of Abbreviations....................60
7. REFERENCES....................62

LIST OF TABLES

Table 1: Estimands....................19
Table 2: Sensitivity Analyses....................22
Table 3: Supplementary Analyses....................24
Table 4: SF-36v2 Domain Scores....................32
Table 5: Abbreviations and Acronyms....................60

LIST OF FIGURES

Figure 1: Study Design Schematic – LN Cohort....................11
Figure 2: Study Design Schematic – IgAN Cohort....................12

VERSION HISTORY

This Statistical Analysis Plan (SAP) for Study ALXN1210-NEPH-202 is based on Protocol Amendment 4, dated 26 Mar 2024.

SAP Version	Version Date	Change	Rationale
1	16 Nov 2021	Not applicable	Original version
2	28 Apr 2023	Randomization stratification factor<br><br>Hematuria<br><br>Immunogenicity analyses<br><br>Other minor edits	Clarified that the primary endpoint analysis will use verified strata and may add the sensitivity analysis using per-randomized strata<br><br>Updated the analysis method for red blood cells in urine, as it is received by the central laboratory as a categorical variable instead of a continuous variable<br><br>Added definitions of Immunogenicity variables include antidrug antibody (ADA) status categories, ADA response categories, ADA, or neutralizing antibody (NAb) incidence and titer over the duration of the study<br><br>For clarity
3	31 Jul 2024	Terminology<br><br>Estimated Glomerular Filtration Rate<br><br>Complete Renal Response/Partial Renal Response/ Overall Renal Response<br><br>Subgroup Analyses<br><br>Interim Analyses<br><br>Other minor edits<br><br>Allowed Concomitant Therapy and Additional Standard-of-Care Therapy	Based on PA3, change background therapy to allowed concomitant therapy; change rescue therapy to additional standard-of-care therapy<br><br>Clarified screen failed values are not used in analysis<br><br>Added CMH test for renal response analysis<br><br>Added SGLT2 use per team's request<br><br>Added early IA for LN cohort based on PA4<br><br>For clarity<br><br>Per team's comment, LN background therapy should be summarized up to study D1

APPROVAL SIGNATURES

PPD PPD

PPD, PPD, PPD

Date dd Mmm yyyy

PPD PPD

PPD PPD, PPD

Date dd Mmm yyyy

PPD PPD

PPD, PPD, PPD
PPD

Date dd Mmm yyyy

1. INTRODUCTION

This Statistical Analysis Plan (SAP) describes the statistical methods for analyzing data for Protocol ALXN1210-NEPH-202, "A Phase 2, Double-Blind, Randomized, Placebo-Controlled Study to Evaluate the Efficacy and Safety of Ravulizumab in Adult Participants With Proliferative Lupus Nephritis (LN) or Immunoglobulin A Nephropathy (IgAN)." Standard data presentation instructions and table, figure, and listing specifications are contained in the Data Presentation Plan (DPP) in a separate document.

The primary efficacy analysis will be performed for each disease-specific cohort at the end of the 26-week Initial Evaluation Period after all participants in the disease-specific cohort have completed or withdrawn from the 26-week Initial Evaluation Period. This analysis will allow for evaluation of the primary endpoint and Phase 3 planning and will have no impact on the progression of this study. The sponsor will be unblinded at this time to conduct the primary analysis. After completion of the Initial Evaluation Period, participants in the LN cohort will continue receiving their randomized study intervention, and both the participants and the investigative site personnel will remain blinded for the remaining 24-week Extension Period. In the IgAN cohort, participants in the placebo treatment group will switch to receive a blinded loading dose of ravulizumab at Week 26 and participants in the ravulizumab treatment group will receive a blinded ravulizumab dose of 900 mg at Week 26. Starting at Week 28, all participants in the IgAN cohort will receive open-label weight-based doses of ravulizumab once every 8 weeks (q8w) until the end of the Extension Period.

The final analysis will occur at the end of the study when all participants have completed the Post-treatment Follow-up Period or withdrawn from the study early and will include all planned analyses that will be presented in the final Clinical Study Report.

1.1. Objectives and Endpoints

Objectives	Endpoints
**Primary (both cohorts)**	
To evaluate the efficacy of ravulizumab compared with placebo to reduce proteinuria in adult participants with LN or IgAN	Percentage change in proteinuria from baseline to Week 26 (based on 24-hour urine collection[s] at each time point)
**Secondary (both cohorts)**	
To evaluate the efficacy of ravulizumab compared with placebo to improve measures of kidney function in adult participants with LN or IgAN	Percentage change in proteinuria from baseline to Week 50 (based on 24-hour urine collection[s] at each time point)
	Percentage of participants with > 30% and > 50% reduction in proteinuria at Week 26 and Week 50 compared to baseline (based on 24-hour urine collection[s] at each time point)
	Change from baseline in estimated glomerular filtration rate (eGFR) at Week 26 and Week 50
	Absolute values and change from baseline in serum C3 and C4 concentrations at Week 26 and Week 50

Objectives	Endpoints
**Secondary (LN cohort only)**	
To evaluate the efficacy of ravulizumab compared with placebo to improve measures of kidney function in adult participants with LN	Percentage of participants meeting the criteria for complete renal response (CRR) at Week 26 and Week 50
	Percentage of participants meeting the criteria for partial renal response (PRR) at Week 26 and Week 50
	Time to urine protein-to-creatinine ratio (UPCR) < 0.5 g/g as measured by spot urine sample
	Percentage of participants achieving corticosteroid taper to 7.5 mg/day at Weeks 14, 26, and 50
	Percentage of participants with renal flare through Week 50
	Percentage of participants with extrarenal systemic lupus erythematosus (SLE) flare through Week 50
	Percentage of participants with treatment failure through Week 50
	Percentage of participants with suboptimal response through Week 50
	Absolute values and change from baseline in serum albumin at Week 26 and Week 50
**Secondary (IgAN cohort only)**	
To evaluate the efficacy of ravulizumab compared with placebo on measures of kidney function in adult participants with IgAN	Percentage of participants meeting the criteria for partial remission at Week 26 and Week 50
**Pharmacokinetic (PK)/pharmacodynamic (PD)/immunogenicity (both cohorts)**	
To characterize the PK/PD of ravulizumab in adult participants with LN or IgAN	Absolute values and change from baseline in total C5 and free C5 concentrations over time
	Absolute values and change from baseline in ravulizumab concentrations over time
To characterize the potential for immunogenicity of ravulizumab in adult participants with LN or IgAN	Incidence of antidrug antibodies (ADAs) over time
**Safety (both cohorts)**	
To characterize the safety and tolerability of ravulizumab in adult participants with LN or IgAN	Incidence of adverse events (AEs) and serious adverse events (SAEs) over time
**Exploratory (both cohorts)**	
To evaluate the efficacy of ravulizumab compared with placebo on hematuria in adult participants with LN or IgAN	Effect on hematuria as measured by:<br>- Absolute value and change from baseline in red blood cells (RBCs) in urine from baseline to Week 26 and Week 50<br>- Percentage of participants with < 10 RBCs
To assess quality of life (QoL) based on participant-reported outcomes in adult participants with LN or IgAN based on treatment with ravulizumab compared with placebo	Change from baseline in the 36-item Short Form Health Survey (SF-36v2) at Week 26 and Week 50
	Change from baseline in the 5-level EuroQol-5 Dimension (EQ-5D-5L) at Week 26 and Week 50
To evaluate complement and autoimmune biomarkers in adult participants with LN or IgAN	Absolute values and change from baseline in levels of biomarkers in blood, urine, and kidney tissue at Week 26 and Week 50

Objectives	Endpoints
**Exploratory (LN cohort only)**	
To assess the efficacy of ravulizumab in exploratory efficacy endpoints	Time to CRR and PRR (using spot UPCR)
	Percentage of participants with overall renal response at Week 26 and Week 50 (CRR and PRR)
	Time to UPCR > 50% decrease from baseline (using spot UPCR)
To assess QoL based on participant-reported outcomes	Change from baseline in the Functional Assessment of Chronic Illness Therapy (FACIT)-Fatigue score at Week 26 and Week 50
To assess the efficacy of ravulizumab in other exploratory endpoints	Absolute values and change from baseline in anti-double-stranded DNA (anti-dsDNA) and anti-C1q antibodies at Week 26 and Week 50
	Histology changes from baseline to Week 50
**Exploratory (IgAN cohort only)**	
To assess the efficacy of ravulizumab in exploratory efficacy endpoints	Slope of eGFR computed from baseline to Week 26 and Week 50

1.2. Study Design

Study ALXN1210-NEPH-202 is a Phase 2, randomized, double-blind, placebo-controlled, multicenter study of ravulizumab in addition to allowed concomitant therapy consistent with the standard of care in 120 adult participants (18 to 75 years of age) with either proliferative LN or IgAN. All participants must be naïve to complement inhibitor treatment and have either a diagnosis of LN with an active flare or IgAN based on kidney biopsy, eGFR ≥ 30 mL/min/1.73 m^2, and proteinuria (defined as UPCR ≥ 1 g/g from one 24-hour urine collection [LN cohort] or as mean protein ≥ 1 g/24-h from 2 valid 24-hour collections [IgAN cohort]). Participants in the IgAN cohort must have been treated with stable doses of the maximum tolerated renin-angiotensin system (RAS)-inhibiting medications and have controlled, stable blood pressure (< 140/90 mmHg) for ≥ 3 months prior to Screening.

Approximately 60 participants in each disease cohort will be randomly assigned in a 2:1 ratio to receive ravulizumab or placebo (40 ravulizumab, 20 placebo). Randomization will be stratified by whether corticosteroid induction treatment was initiated prior to Screening versus during the Screening Period for participants in the LN cohort and by mean proteinuria (1 to 2 g/day versus > 2 g/day) from 2 valid 24-hour urine collections during the Screening Period for participants in the IgAN cohort.

The study consists of an up to 6-week Screening Period, a 26-week Initial Evaluation Period, a 24-week Extension Period, and a 36-week Post-treatment Follow-up Period. Thus, the total treatment duration is 50 weeks and the total study duration is up to 86 weeks (Figure 1 and Figure 2).

During the Initial Evaluation Period, all participants will receive a weight-based loading dose of ravulizumab or placebo on Day 1, followed by maintenance doses of ravulizumab or placebo on Day 15 and then q8w thereafter. Loading and maintenance doses will be determined based on body weight. All participants will receive allowed concomitant therapy consistent with the standard of care for participants with LN and IgAN throughout the study.

During the 24-week Extension Period, participants in the LN cohort will continue to receive their randomized allocation of study intervention (ravulizumab or placebo) q8w. For the IgAN cohort, participants in the placebo treatment group will receive a blinded loading dose of ravulizumab at Week 26, and participants in the ravulizumab treatment group will receive a blinded ravulizumab dose of 900 mg at Week 26. Starting at Week 28, all participants in the IgAN cohort will receive open-label weight-based doses of ravulizumab q8w until the end of the Extension Period.

During the 36-week Post-treatment Follow-up Period, all participants will continue to receive standard of care and will be monitored for safety, clinical events of interest, and kidney function. Participants who discontinue study intervention early and agree to remain in the study should continue to attend their scheduled protocol visits until Week 50. During these visits, all assessments except for administration of study intervention should be performed according to the Schedule of Assessments. Participants who withdraw from the study will be followed for safety until 8 weeks after the last dose of study intervention. The end of study is defined as the last participant's last visit in the Post-treatment Follow-up Period.

In the LN cohort, participants will receive additional standard-of-care therapy in the event of a protocol-defined renal flare or severe extrarenal SLE flare. After Week 26, participants may also receive additional standard-of-care therapy for protocol-defined suboptimal response.

The primary objective of the study is to evaluate the efficacy of ravulizumab compared with placebo to reduce proteinuria in adult participants with LN or IgAN.

Figure 1: Study Design Schematic – LN Cohort

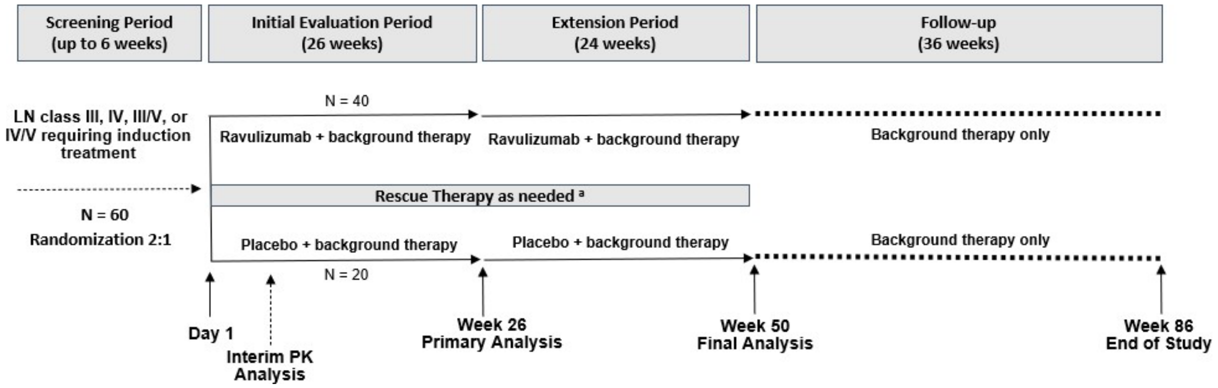

Note: Randomization will be stratified by whether corticosteroid induction treatment was initiated prior to Screening versus during the Screening Period. Allowed concomitant therapy consists of corticosteroids and mycophenolate mofetil. Weight-based dosing regimen (see Protocol Section 1.1) will be based on the last recorded study visit body weight.

[a] Participants will receive additional standard-of-care therapy in the event of a protocol-defined renal flare or severe extrarenal SLE flare. Approved novel treatment(s) for LN is allowed. After Week 26, additional standard-of-care therapy for participants with suboptimal response is allowed at the clinical discretion of the Investigator in conversation with the Medical Monitor.

Abbreviations: LN = lupus nephritis; PK = pharmacokinetics

Figure 2: Study Design Schematic – IgAN Cohort

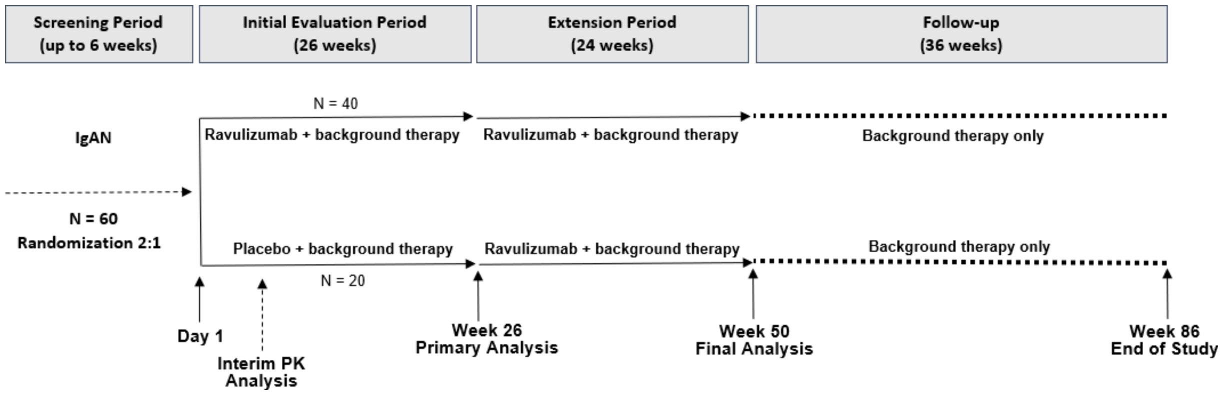

Note: Randomization will be stratified by mean proteinuria (1 to 2 g/day versus > 2 g/day) based on 2 valid 24-hour urine collections during the Screening Period. Allowed concomitant therapy consisting of stable maximally tolerated dose of ACE inhibitors or ARBs. Weight-based dosing regimen (see Protocol Section 1.1) will be based on the last recorded study visit body weight.

Abbreviations: ACE = angiotensin-converting enzyme; ARB = angiotensin II receptor blocker; IgAN = immunoglobulin A nephropathy; PK = pharmacokinetics

2. STATISTICAL HYPOTHESES

The primary hypothesis for this study is that ravulizumab is superior to placebo in decreasing proteinuria. Hypothesis testing will be one-sided and performed at the 0.05 level of significance.

3. SAMPLE SIZE DETERMINATION

This study plans to enroll 60 participants in both the IgAN and LN cohorts in a 2:1 ratio to ravulizumab and placebo for a total of 120 participants.

In March 2018, a National Kidney Foundation-sponsored workshop on surrogate endpoints for clinical studies in early stages of chronic kidney disease proposed that a 20% to 30% reduction in the geometric mean of albuminuria or proteinuria was likely to be necessary to ensure a significant treatment effect on the clinical outcome (Levey, 2020).

Individual patient data from relevant studies in IgAN was previously used to estimate variability and expected changes in proteinuria for patients treated with placebo in addition to background standard-of-care treatment (Fellstrom, 2017). Based on these data, the geometric mean (GM) of the ratio of 26-week to baseline proteinuria values is assumed to be 0.85 (ie, a 15% reduction in proteinuria) for the placebo treatment group. In order to target at least a 30% relative treatment effect (ie, 1 minus geometric mean ratio [GMR] of ravulizumab to placebo at Week 26), the GM of the ratio of 26-week to baseline is assumed to be 0.55 (ie, a 45% reduction in proteinuria) for the ravulizumab treatment group.

Expected changes in proteinuria and associated variability were not available on the log-scale for patients with LN; however, 2 studies of patients with LN who were treated with mycophenolate mofetil (MMF) showed mean proteinuria values of approximately 4.0 g/day at baseline and approximately 2.0 g/day at Week 24 (Appel, 2009; Ginzler, 2005). The GM of the ratio of 26-week to baseline proteinuria values is assumed to be 0.60 (ie, a 40% reduction in proteinuria) for the placebo treatment group. In order to target at least a 30% relative treatment effect, the GM of the ratio of 26-week to baseline proteinuria values is assumed to be 0.40 (ie, a 60% reduction in proteinuria) for the ravulizumab treatment group.

Sample size calculations are based on a 1-sided, 2-sample t-test of log-transformed proteinuria values. The log change from baseline in proteinuria is calculated as log(0.85) and log(0.55) for the placebo and ravulizumab treatment groups, respectively, for the IgAN cohort and as log(0.60) and log(0.40) for the placebo and ravulizumab treatment groups, respectively, for the LN cohort. A common standard deviation (SD) of log change is assumed to be 0.60 (Fellstrom, 2017). Under these assumptions and an anticipated 10% drop out rate, a sample size of 60 participants (40 participants randomized to ravulizumab, 20 participants randomized to placebo) will provide approximately 80% power to detect a treatment difference with a 1-sided significance level of 0.05 in each cohort.

4. ANALYSIS SETS

The following populations are defined for this study:

Analysis Set	Description
Randomized Set	All randomized participants. Participants will be analyzed as randomized for reporting disposition.
Full Analysis Set (FAS)	All randomized participants who receive at least 1 dose of the study intervention. Participants will be analyzed as randomized for reporting efficacy data.
Modified Full Analysis Set (mFAS)	All FAS participants excluding participants who were impacted by COVID-19 as follows:<br>1. Participants who received a dose of ravulizumab ≥ 28 days after the scheduled dosing time point or missed a dose altogether during the 26-week Initial Evaluation Period due to COVID-19<br>2. Participants who withdrew from the study early during the 26-week Initial Evaluation Period due to COVID-19<br>3. Participants who received concomitant treatments for COVID-19 during the 26-week Initial Evaluation Period that could potentially affect efficacy<br>4. Participants who were hospitalized due to a COVID-19-related AE during the 26-week Initial Evaluation Period<br>Inclusion in the mFAS will be determined for each participant prior to database lock.
Per-Protocol Set (PPS)	All randomized participants who receive at least 1 dose of study intervention and without important protocol deviations who meet the following criteria:<br>1. For LN cohort, satisfied the following inclusion criteria (IC)/exclusion criteria (EC):<br>– IC #10: Diagnosis of LN Class III or IV confirmed by biopsy<br>– IC #12: Proteinuria with UPCR ≥ 1 g/g based on one 24-hour urine collection during the Screening Period<br>– EC #1: eGFR < 30 mL/min/1.73 m^2 during Screening<br>– EC #20: Received any of the specified treatments (see Protocol Section 5.2.2)<br>2. For IgAN cohort, satisfied the following inclusion/exclusion criteria:<br>– IC #13: Established diagnosis of primary IgAN based on kidney biopsy<br>– IC #14: Mean proteinuria ≥ 1 g/day on 2 complete and valid 24-hour urine collections during the Screening Period

Analysis Set	Description
	– IC #16: Compliance with stable and optimal dose of RAS inhibitor treatment for ≥ 3 months prior to Screening<br>– EC #1: eGFR $<$ 30 mL/min/1.73 m^2 during Screening<br>3. For LN cohort, received appropriate additional standard-of-care therapy, if required per protocol, during the 26-week Initial Evaluation Period<br>4. Received appropriate allowed concomitant therapy during the 26-week Initial Evaluation Period<br>5. Received all planned number of doses during the 26-week Initial Evaluation Period<br>6. Received correct treatment per randomization schedule<br>7. Did not receive any disallowed medications or therapies during the 26-week Initial Evaluation Period (see Protocol Section 6.7.2)<br>8. Was not unblinded to treatment allocation during the 26-week Initial Evaluation Period<br>Inclusion in the PPS will be determined for each participant prior to database lock.
Safety Set	All participants who receive at least 1 dose of study intervention. Participants will be analyzed according to the study intervention they actually received for reporting exposure and safety data.
PK Analysis Set	All participants who receive at least 1 dose of study intervention and who have evaluable PK data.
PD Analysis Set	All participants who receive at least 1 dose of study intervention and who have evaluable PD data.

5. STATISTICAL ANALYSES

5.1. General Considerations

All data collected in this study will be presented separately by disease cohort using summary tables, figures, and data listings. All analyses will be performed using Statistical Analysis Software® (SAS) release, V 9.4 or higher (SAS Institute Inc., Cary, NC, USA) or other validated statistical software.

All efficacy and safety analyses will be summarized separately for the Initial Evaluation Period, Extension Period, and Post-treatment Follow-up Period. Limited data will be collected during the Post-treatment Follow-up Period and will be summarized descriptively, as appropriate.

The analyses for participants in the LN cohort and participants with IgAN cohort will be conducted and reported separately.

Continuous variables will be summarized using descriptive statistics, including number of observations and mean, SD, median, minimum, and maximum values. Categorical variables will be summarized by frequency counts and percentage of patients.

Unless otherwise specified, data collected at an unscheduled visit will be included in by-patient listings but not in the summary tabulations. However, unscheduled study visits will be used in the calculation of baseline values.

Summaries of study and participant characteristics (eg, demographics and baseline characteristics, medical history, protocol deviations) are described in Section 6.2.

5.1.1. Data Presentation for the Initial Evaluation Period

Data summaries for the Initial Evaluation Period will be presented by randomized treatment group (ie, "Ravulizumab" and "Placebo") and overall, if applicable. For the IgAN cohort, summaries will be presented by treatment sequence (ie, "Ravulizumab to Ravulizumab" and "Placebo to Ravulizumab"). Baseline is defined as the last value prior to the start of study intervention. All assessments for Week 26 will be performed prior to dosing. Dosing on Week 26 will be considered the start of the Extension Period.

5.1.2. Data Presentation for the Extension Period

Data summaries for the Extension Period will be presented by randomized treatment group and overall, if applicable. For the IgAN cohort, summaries will be presented by treatment sequence (ie, "Ravulizumab to Ravulizumab" and "Placebo to Ravulizumab"). In addition, baseline for the IgAN cohort will be defined as the last value prior to their initial exposure to ravulizumab.

5.1.3. Data Presentation for the Post-treatment Follow-up Period

Data summaries for the Post-treatment Follow-up Period will be presented by randomized treatment group and overall, if applicable. For the IgAN cohort, summaries will be presented by treatment sequence (ie, "Ravulizumab to Ravulizumab" and "Placebo to Ravulizumab").

5.1.4. Handling of Dropouts or Missing Data

No imputation will be performed for missing baseline values.

To derive duration of disease, partial date of diagnosis will be imputed. If Day is missing, then impute to the 1st. If Day and Month are missing, then impute to Jan 1st. Concomitant medication start date and AE start date will follow the same imputation rule to derive the study phase.

Missing data for QoL instruments will be handled as specified in the instructions for each instrument in Section 6.3.

In addition, the following intercurrent events may occur during the study:

1. Receipt of additional standard-of-care therapy (LN cohort only)
2. Treatment discontinuation

For the primary efficacy endpoint, only data up to the point of additional standard-of-care therapy will be included as observed. For participants in the LN cohort who receive additional standard-of-care therapy, the proteinuria value from the 24-hour urine collection performed at the time of renal flare or severe extrarenal SLE flare will be carried forward to subsequent visits. Data occurring on or after treatment discontinuation will be assumed missing-at-random (MAR) and imputed using multiple imputation as described in Section 5.3.2. For participants who experience multiple intercurrent events, data will be handled according to the first intercurrent event.

Additional sensitivity analyses are specified in Section 5.3.3.

For secondary responder endpoints, participants who receive additional standard-of-care therapy or discontinue treatment are assigned as nonresponders regardless of whether there is any response observed after these protocol-defined intercurrent events. For missing postbaseline data not due to intercurrent events, no imputation will be performed.

A summary of the primary and secondary estimands for this study is provided in Table 1.

Table 1: Estimands

Estimand	Treatment	Population	Variable	Handling of IE	Summary Measure
Primary	Ravulizumab or placebo	Full Analysis Set	Percentage change from baseline to Week 26 in proteinuria (both cohorts)	Data on or after receipt of additional standard-of-care therapy (LN cohort only) will be imputed using the value collected at the time of renal flare or severe extrarenal SLE flare<br><br>Data on or after treatment discontinuation will be imputed using multiple imputation	Difference in mean of the variable between treatment groups
Secondary (key continuous endpoints)	Ravulizumab or placebo	Full Analysis Set	Percentage change from baseline to Week 50 in proteinuria (both cohorts)<br><br>Change from baseline in eGFR at Week 26 and Week 50 (both cohorts)	Data on or after receipt of additional standard-of-care therapy (LN cohort only) will be imputed using the value collected at the time of renal flare or severe extrarenal SLE flare (or suboptimal after Week 26, if applicable)<br><br>Data on or after treatment discontinuation will be assumed missing and handled in the MMRM as MAR	Mean of the variable in each treatment group

Table 1: Estimands

Estimand	Treatment	Population	Variable	Handling of IE	Summary Measure
Secondary (key binary endpoints)	Ravulizumab or placebo	Full Analysis Set	Percentage of participants with > 30% and > 50% reduction in proteinuria at Week 26 and Week 50 compared to baseline (both cohorts)<br><br>Percentage of participants meeting the criteria for CRR at Week 26 and Week 50 (LN cohort only)<br><br>Percentage of participants meeting the criteria for PRR at Week 26 and Week 50 (LN cohort only)<br><br>Percentage of participants meeting the criteria for Partial Remission at Week 26 and Week 50 (IgAN cohort only)	Participants who receive additional standard-of-care therapy (LN cohort only) or discontinue treatment are assigned as nonresponders	Proportion of the variable in each treatment group

Abbreviations: CRR = complete renal response; eGFR = estimated glomerular filtration rate; IE = intercurrent events; LN = lupus nephritis; MAR = missing-at-random; MMRM = mixed effect model for repeated measures; PK = pharmacokinetics; PRR = partial renal response

The secondary estimands for all other secondary endpoints are similar to above except the intercurrent events will be ignored and all data occurring on or after the intercurrent event will be analyzed as observed.

5.2. Study Participants

A summary of disposition will be presented by treatment group and overall and will include the number and percentage of participants screened, screen failed, randomized, and treated. The number and percentage of participants who completed the study or discontinued from the study (along with the primary reason for discontinuation) will be presented. Discontinuation status will be presented overall as well as separately for discontinuations impacted by COVID-19. Disposition will be presented separately for the Initial Evaluation Period, Extension Period, and Post-treatment Follow-up Period.

The number and percentage of participants in each defined analysis set will also be presented.

A summary of enrolled participants by region will be presented by treatment group and overall.

Participants who are randomized but did not satisfy the inclusion/exclusion criteria will be presented by treatment group and overall for each criterion not satisfied.

By-participant data listings for disposition and analysis populations will be provided, as well as a listing of participants who did not satisfy the inclusion/exclusion criteria.

5.3. Primary Endpoint Analysis

The primary efficacy endpoint analysis will be based on the FAS.

The analyses for participants in the LN cohort and participants in the IgAN cohort will be conducted and reported separately. Participants in each disease-specific cohort will be analyzed as randomized, regardless of actual treatment received.

The randomization stratification factor will be based on strata verified by central laboratory and allowed concomitant therapy data from electronic case report forms (eCRFs). Sensitivity analysis may be performed using strata as randomized in IXRS (Interactive Voice/Web Response System).

5.3.1. Percentage Change from Baseline in Proteinuria

The primary endpoint for the study is percentage change from baseline to Week 26 in proteinuria based on 24-hour urine collection(s) at each timepoint.

Proteinuria will be summarized by absolute urine protein in g/day as well as by UPCR in g/g for each disease-specific cohort.

For participants in the LN cohort, the primary endpoint will be based on UPCR in g/g derived from a single 24-hour urine collection at Screening and the mean of 2 separate 24-hour urine collections within 2 weeks of the Week 26 visit.

For participants in the IgAN cohort, the primary endpoint will be based on absolute urine protein in g/day derived from the mean of 2 valid 24-hour urine collections during the Screening Period and within 2 weeks of the Week 26 visit.

5.3.2. Main Analytical Approach

The primary analysis of the primary efficacy endpoint (percentage change from baseline in proteinuria at Week 26) will be conducted for each disease-specific cohort after all participants have completed the protocol-required assessments in the Initial Evaluation Period.

For the primary efficacy endpoint, when two 24-hour urine collections are required and only 1 collection is available at the visit, then this value will be used for analysis. When more than 2 collections are available at the visit, only 2 valid collections recorded in the eCRF will be used for analysis.

To reduce skewness, the natural logarithm will be used to transform proteinuria values before analysis. An analysis of covariance (ANCOVA) will be used for the primary efficacy endpoint to compare reductions in proteinuria between the ravulizumab and placebo treatment groups. The ANCOVA model will include change from baseline in log-transformed proteinuria as the response variable and will adjust for baseline log proteinuria and the randomization stratification factor. The treatment effect will be evaluated using the least squares mean difference between treatment groups. The point estimate and 2-sided 90% confidence interval (CI) for the mean difference of log-transformed proteinuria will be back transformed (via exponentiation) to obtain the GMR and corresponding 2-sided 90% CI. The values will then be expressed as percentage change in adjusted geometric mean of proteinuria at Week 26 relative to baseline.

Data collected on or after receipt of additional standard-of-care therapy (for LN cohort) will be imputed using the proteinuria value from the 24-hour urine collection performed at the time of renal flare or extrarenal SLE flare. Data collected on or after treatment discontinuation will be assumed MAR and imputed using multiple imputation.

5.3.3. Sensitivity Analysis

Table 2 shows the sensitivity analyses that will be produced for the primary endpoint.

Table 2: Sensitivity Analyses

Sensitivity Analysis	Methodology	Population	Handling of Intercurrent Events
Placebo-based multiple imputation	ANCOVA	FAS	- Data collected on or after receipt of additional standard-of-care therapy (for the LN cohort only) will be imputed using the value collected at the time of renal flare or extrarenal SLE flare<br>- Data collected on or after treatment discontinuation will be imputed using placebo-based multiple imputation
Tipping point analysis	ANCOVA	FAS	- Participants who receive additional standard-of-care therapy (for the LN cohort only), discontinue treatment, withdraw early or die are assumed to have outcomes that are worse than otherwise similar participants who remain in the study or did not receive additional standard-of-care therapy or discontinue treatment

Table 2: Sensitivity Analyses

Sensitivity Analysis	Methodology	Population	Handling of Intercurrent Events
Per-randomized stratification analysis	ANCOVA	FAS	- Data collected on or after receipt of additional standard-of-care therapy (LN cohort only) will be imputed using the value collected at the time of renal flare or severe extrarenal SLE flare<br>- Data collected on or after treatment discontinuation will be imputed using multiple imputation

Abbreviations: ANCOVA = analysis of covariance; FAS = Full Analysis Set; LN = lupus nephritis; SLE = systemic lupus erythematosus

Further details of these sensitivity analyses are below.

- Placebo-based multiple imputation: The primary endpoint will be analyzed in the same manner as the primary analysis, except data collected on or after treatment discontinuation will be assumed missing and imputed using placebo-based multiple imputation where it will be assumed that outcomes occurring after treatment discontinuation will follow the trajectory of outcomes similar to the one in the placebo treatment group, taking into account observed values prior to treatment discontinuation (Ratitch, 2014).
- Tipping point analysis: An additional sensitivity analysis will be performed based on the delta-adjusted stress testing method (tipping point analysis). This approach assumes that patients who discontinue from ravulizumab treatment or who receive additional standard-of-care therapy (for the LN cohort only) experience worsening, defined by a prespecified adjustment (delta) in the primary efficacy endpoint compared with the observed efficacy score of patients that continue the study to next visit. For each value of delta, the treatment effect will be determined, and the value of delta for which the nominal 2-sided p-value crosses 0.10, will be considered as the 'tipping point' in the sense that the conclusion drawn from the primary analysis is reversed when patients who drop out or who receive additional standard-of-care therapy are assumed to experience this fixed adjustment after the discontinuation visit or receipt of additional standard-of-care therapy. After such a tipping point is determined, clinical judgment will be applied as to the plausibility of the assumptions underlying this tipping point. This methodology is expected to inform what it would take to overturn study conclusions based on varying assumptions about missing data (Ratitch, 2013; Ratitch, 2014).
- Per-randomized stratification analysis: The primary endpoint will be analyzed in the same manner as the primary analysis, except using per-randomized stratification factor from IXRS in the analysis.

5.3.4. Supplementary Analysis

Table 3 shows the supplementary analyses that will be produced for the primary endpoint.

Table 3: Supplementary Analyses

Supplemental Analysis	Methodology	Population	Handling of Intercurrent Events
Randomized Set analysis	ANCOVA	Randomized Set	- Data collected on or after receipt of additional standard-of-care therapy (for the LN cohort only) will be imputed using the value collected at the time of renal flare or extrarenal SLE flare<br>- Data collected on or after treatment discontinuation will be imputed using multiple imputation
mFAS analysis	ANCOVA	mFAS	- Data collected on or after receipt additional standard-of-care therapy (for the LN cohort only) will be imputed using the value collected at the time of renal flare or extrarenal SLE flare<br>- Data collected on or after treatment discontinuation will be imputed using multiple imputation
PPS analysis	ANCOVA	PPS	- Data collected on or after receipt of additional standard-of-care therapy (for the LN cohort only) will be imputed using the value collected at the time of renal flare or extrarenal SLE flare<br>- Data collected on or after treatment discontinuation will be imputed using multiple imputation
Treatment policy analysis	ANCOVA	FAS	- All observed data is used regardless of receipt of additional standard-of-care therapy (for the LN cohort only) or treatment discontinuation.

Abbreviations: ANCOVA = analysis of covariance; mFAS = Modified Full Analysis Set; FAS = Full Analysis Set; LN = lupus nephritis; PPS = Per-protocol Set; SLE = systemic lupus erythematosus

Further details of these supplementary analyses are below.

- Randomized Set analysis: The primary endpoint will be analyzed in the same manner as the primary analysis, except using the Randomized Set instead of the FAS. The Randomized Set will only be analyzed if the FAS population excludes more than 10% of the Randomized Set.
- mFAS analysis: The primary endpoint will be analyzed in the same manner as the primary analysis, except using the mFAS instead of the FAS. The mFAS will only be analyzed if this population excludes more than 10% of the FAS.
- PPS analysis: The primary endpoint will be analyzed in the same manner as the primary analysis, except using the PPS instead of the FAS.
- Treatment policy analysis: The primary endpoint will be analyzed in the same manner as the primary analysis, except all observed data will be included, regardless of receipt of additional standard-of-care therapy or treatment discontinuation.

5.4. Secondary Endpoint Analysis

The secondary efficacy analyses will be based on the FAS. These analyses will be descriptive in nature, and no adjustment for multiplicity will be performed.

The analyses for participants in the LN cohort and participants in the IgAN cohort will be conducted and reported separately. Participants in each disease-specific cohort will be analyzed as randomized, regardless of actual treatment received.

Summary statistics will be computed and displayed by treatment group and by visit, where applicable. Descriptive statistics for continuous variables will minimally include the number of participants, mean, SD, minimum, median, and maximum. For categorical variables, frequencies and percentages will be presented.

For the IgAN cohort, participants initially randomized to the placebo treatment group will receive ravulizumab in the Extension Period. Therefore, analysis of the secondary endpoints during the Extension Period (Week 26 through Week 50) will be summarized separately for each treatment group assigned during the Initial Evaluation Period (Baseline through Week 26). During the Extension Period, baseline for the placebo treatment group will be redefined as the last measurement taken before the first dose of ravulizumab during the Extension Period (ie, the Week 26 measurement).

5.4.1. Proteinuria

The percentage change from baseline in proteinuria at Week 50 will be analyzed using a mixed-effect model for repeated measures (MMRM) using all available longitudinal data (either complete or partial). The model will include change from baseline in log-transformed proteinuria as the response variable and fixed, categorical effects of treatment group, randomization stratification factor, visit, and treatment group by visit interaction as well as a fixed, continuous effect of baseline log proteinuria as a covariate. An unstructured covariance matrix will be used to model the correlations among repeated measurements within each participant. If this analysis fails to converge, a first-order autoregressive covariance matrix will be used. The Kenward-Roger approximation will be used to estimate denominator degrees of freedom.

For the IgAN cohort, participants initially randomized to the placebo treatment group will switch to receive ravulizumab at Week 26. Therefore, percentage change from baseline in proteinuria at Week 50 will be summarized descriptively for each treatment group and for each visit from the MMRM analysis, and no formal treatment comparison will be made.

For the LN cohort, the treatment effect will be evaluated using a contrast for treatment group-by-visit term at Week 50. The point estimate and 2-sided 90% CI for the mean difference of log-transformed proteinuria will be back-transformed (via exponentiation) to obtain the GMR and corresponding 2-sided 90% CI. The values will then be expressed as percentage change in adjusted geometric mean of proteinuria at Week 50 relative to baseline.

This analysis will be performed 2 ways:

- Data collected on or after receipt of additional standard-of-care therapy (for the LN cohort only) will be imputed using the value collected at the time of renal flare, extrarenal SLE flare, suboptimal response, and data collected on or after treatment discontinuation will be assumed missing and handled in the MMRM as MAR.
- Data collected on or after receipt of additional standard-of-care therapy or treatment discontinuation will be assumed missing and handled in the MMRM as MAR.

In addition to the proteinuria analyses based on 24-hour urine collections, the percentage change from baseline in spot UPCR at each scheduled visit will be analyzed using an MMRM in a similar manner as above.

In addition to the MMRM analysis, observed values, change from baseline, and percent change from baseline in proteinuria will be summarized descriptively by treatment group at baseline and at each postbaseline time point separately by study phase.

The trajectory of proteinuria over time will be visually presented by plotting the mean proteinuria based on 24-hour urine collections at each time point by treatment group. This plot will be repeated for proteinuria based on spot UPCR.

The percentage of participants with > 30% reduction in proteinuria and > 50% reduction in proteinuria at Week 26 and Week 50 will be summarized by treatment group by calculating the point estimate and 2-sided 95% CI, based on exact confidence limits using the Clopper-Pearson method. Participants who receive additional standard-of-care therapy (for the LN cohort only) or discontinue treatment will be considered nonresponders from the point of treatment failure or treatment discontinuation onward.

5.4.2. Estimated Glomerular Filtration Rate

Kidney function evaluated by eGFR will be summarized at baseline and each postbaseline time point by treatment group using descriptive statistics for the observed value as well as the change from baseline and percentage change from baseline.

For participants in the LN cohort, baseline eGFR will be defined as the lowest measurement available prior to the first dose of study intervention. Screen failed values will not be considered.

For participants in the IgAN cohort, baseline eGFR will be defined as the mean of the Screening and Day 1 measurements.

For participants requiring dialysis for acute kidney injury, a value of 10 mL/min/1.73 m^2 will be imputed for while participants are on dialysis (ie, from the first day of dialysis through 5 days after the end of dialysis).

The longitudinal changes in eGFR will also be analyzed using the same MMRM method specified in Section 5.4.1.

This analysis will be performed 2 ways:

- Data collected on or after receipt of additional standard-of-care therapy (for the LN cohort only) will be imputed using the value collected at the time of renal flare, extrarenal SLE flare, suboptimal response, and data collected on or after treatment discontinuation will be assumed missing and handled in the MMRM as MAR.
- Data collected on or after receipt of additional standard-of-care therapy or treatment discontinuation will be assumed missing and handled in the MMRM as MAR.

The trajectory of eGFR over time will be visually presented by plotting the mean eGFR at each time point by treatment group.

5.4.3. Serum C3 and C4 Concentrations

Serum C3 and C4 concentrations will be summarized at baseline and each postbaseline time point by treatment group using descriptive statistics for the observed value as well as the change from baseline. There will be no imputation for missing data or data collected on or after the receipt of additional standard-of-care therapy (for LN cohort) or treatment discontinuation.

The trajectory of serum C3 and serum C4 over time will be visually presented by plotting the mean values at each time point by treatment group.

5.4.4. Complete Renal Response (LN Cohort Only)

For the LN cohort, CRR is defined as meeting all 3 of the following criteria:

- A decrease in mean UPCR to ≤ 0.5 g/g based on two 24-hour urine collections obtained within 2 weeks prior to the study visit (Week 26 or Week 50).
- eGFR > 60 mL/min/1.73 m^2 or no eGFR reduction ≥ 20% from the baseline value based on the mean of 2 values. The first eGFR value must be obtained within 2 weeks prior to the study visit (Week 26 or Week 50), and the second eGFR value will be obtained on the study visit (Week 26 or Week 50).
- No treatment failure (as defined in Section 5.4.11).

Participants who receive additional standard-of-care therapy or discontinue treatment prior to the endpoint assessment will be considered nonresponders.

The percentage of participants meeting the criteria for CRR as well as individual components of CRR will be analyzed using a CMH test accounting for the stratification factor at Week 26 and Week 50 and will be summarized by treatment group by calculating the point estimate and 2-sided 95% CI based on exact confidence limits using the Clopper-Pearson method.

A bar chart will also be provided displaying the proportion of participants meeting the criteria for CRR as well as individual components of CRR at Week 26 and Week 50 by treatment group.

5.4.5. Partial Renal Response (LN Cohort Only)

For the LN cohort, PRR is defined for participants not achieving CRR as meeting all 3 of the following criteria:

- A decrease in UPCR > 50% compared to the baseline value based on mean of two 24-hour urine collections obtained within 2 weeks prior to the study visit (Week 26 or Week 50).
- eGFR > 60 mL/min/1.73 m^2 or no eGFR reduction ≥ 20% from the baseline value based on the mean of 2 values. The first eGFR value must be obtained within 2 weeks prior to the study visit (Week 26 or Week 50) and the second eGFR value will be obtained on the study visit (Week 26 or Week 50).
- No treatment failure (as defined in Section 5.4.11).

Participants who receive additional standard-of-care therapy or who discontinue treatment prior to the endpoint assessment will be considered nonresponders.

The percentage of participants meeting the criteria for PRR will be analyzed using a CMH test accounting for the stratification factor at Week 26 and Week 50 and will be summarized by treatment group by calculating the point estimate and 2-sided 95% CI based on exact confidence limits using the Clopper-Pearson method.

A bar chart will also be provided displaying the proportion of participants meeting the criteria for PRR as well as individual components of PRR at Week 26 and Week 50 by treatment group.

5.4.6. Time to UPCR < 0.5 g/g (LN Cohort Only)

For the LN cohort, time to UPCR ≤ 0.5 g/g will be summarized based on spot urine samples. Participants will be assigned as responders at the time of achievement of UPCR < 0.5 g/g or censored at the earliest of their treatment discontinuation time, receipt of additional standard-of-care therapy, study withdrawal, or death or at Week 50 if they have not responded or received additional standard-of-care therapy by then.

A Kaplan-Meier cumulative distribution curve will be generated for treatment group, and a log-rank test comparing the curves will be performed. The corresponding summary table will present by treatment group the cumulative distribution function (CDF) estimate, the number of participants at risk, the number of participants responding, and the number of participants censored at each postbaseline time point. The table will also present the first quartile, median, and third quartile, along with 2-sided 95% CI, of time to UPCR ≤ 0.5 g/g.

5.4.7. Corticosteroid Taper (LN Cohort Only)

For the LN cohort, the percentage of participants receiving ≤ 7.5 mg/day will be summarized at Week 14, Week 26, and Week 50 by treatment group by calculating the point estimate and 2-sided 95% CI, based on exact confidence limits using the Clopper-Pearson method.

5.4.8. Renal Flare (LN Cohort Only)

For the LN cohort, renal flare is determined in the opinion of the Investigator in addition to the criteria outlined below and will be recorded on the Renal Flare eCRF:

- For participants who achieve CRR, a renal flare is the reproducible recurrence of proteinuria ≥ 1 g/g.
- For all other participants, a renal flare is either of the following:
  - Reproducible increase of serum creatinine > 25% higher than baseline or above the upper limit of normal, plus any 1 of the following:
    - Reproducible proteinuria ≥ 75% higher than baseline
    - Worsening active urinary sediment compared to baseline as defined by an increase of ≥ 5 RBCs/high power field or new RBC casts (based on local laboratory results from at least 2 samples)
    - Kidney biopsy newly conducted since the biopsy used for eligibility demonstrating LN Class III or IV activity
  - Reproducible doubling of the UPCR from a 24-hour urine collection compared with the lowest previous value obtained after the first dose of study intervention.

Reproducibility of proteinuria requires that the proteinuria based on a UPCR from a morning spot urine collection is confirmed by UPCR calculated on a 24-hour urine collection obtained within a 2-week period.

Reproducibility of serum creatinine requires 2 blood tests within a 2-week period.

The percentage of participants meeting the criteria for protocol-defined renal flare through Week 50 will be summarized by treatment group by calculating the point estimate and 2-sided 95% CI, based on exact confidence limits using the Clopper-Pearson method.

5.4.9. Extrarenal SLE Flare (LN Cohort Only)

The Systemic Lupus Erythematosus Disease Activity Index Safety of Estrogen in Lupus Erythematosus National Assessment (SELENA) Modification (SLEDAI-2K) tool assesses disease activity across 24 disease descriptors, with a total score ranging from 0 to 105. For the LN cohort, 18 extrarenal disease descriptors of the SLEDAI-2K assessment will be used for the determination of extrarenal SLE flare. An extrarenal SLE flare is defined as an increase in SLEDAI-2K ≥ 4 points that is not accounted for by the renal disease descriptors of proteinuria, hematuria, urinary cellular casts, and hypocomplementemia or an increase in anti-double-stranded DNA (anti-dsDNA) antibody level. Participants who meet the criteria for extrarenal SLE flare may receive additional standard-of-care therapy, if considered clinically appropriate by the Investigator. If additional standard-of-care therapy is administered, the event is considered a severe extrarenal SLE flare. All instances are recorded on the Extrarenal SLE Flare eCRF.

The trajectory of SLEDAI-2K score (excluding renal disease descriptors) over time will be visually presented by plotting the mean values at each time point by treatment group.

The percentage of participants meeting the criteria for protocol-defined extrarenal SLE flare and severe extrarenal SLE flare through Week 50 will be summarized by treatment group by calculating the point estimate and 2-sided 95% CI, based on exact confidence limits using the Clopper-Pearson method.

5.4.10. Suboptimal Response (LN Cohort Only)

For the LN cohort, suboptimal response is determined in the opinion of the Investigator in addition to the criteria outlined below after the Week 26 visit:

- Reproducible proteinuria ≤ 25% decreased compared to baseline

Reproducibility of proteinuria requires that the proteinuria based on a UPCR from a morning spot urine collection is confirmed by a central laboratory UPCR calculated on a 24-hour urine collection obtained within a 2-week period.

The percentage of participants meeting the criteria for suboptimal response through Week 50 will be summarized by treatment group by calculating the point estimate and 2-sided 95% CI, based on exact confidence limits using the Clopper-Pearson method.

5.4.11. Treatment Failure (LN Cohort Only)

For the LN cohort, treatment failure is defined as receipt of additional standard-of-care therapy at any time up to Week26 or Week 50 for protocol-defined renal flare, severe extrarenal SLE flare, or suboptimal response as recorded on the Rescue Therapy eCRF.

Increase in corticosteroids for extrarenal SLE flare not meeting the protocol definition of severe extrarenal SLE flare, renal flare not meeting protocol definition for renal flare, lack of response not meeting the protocol definition for suboptimal response, other medical conditions or surgery limited to ≤ 14 days duration are **not** included in treatment failure.

The percentage of participants meeting the criteria for treatment failure through Week 50 will be summarized by treatment group by calculating the point estimate and 2-sided 95% CI, based on exact confidence limits using the Clopper-Pearson method.

5.4.12. Serum Albumin (LN Cohort Only)

Serum albumin will be summarized at baseline and each postbaseline time point by treatment group using descriptive statistics for the observed value as well as the change from baseline.

There will be no imputation for missing data or data collected on or after the receipt of additional standard-of-care therapy (for LN cohort) or treatment discontinuation.

The trajectory of serum albumin over time will be visually presented by plotting the mean values at each time point by treatment group.

5.4.13. Partial Remission (IgAN Cohort Only)

For the IgAN cohort, Partial Remission is defined as mean proteinuria < 1 g/day based on 2 valid 24-hour urine collections obtained within 2 weeks prior to the study visit (Week 26 or Week 50).

Participants who discontinue treatment prior to the endpoint assessment will be considered nonresponders.

The percentage of participants meeting the criteria for partial remission will be summarized at Week 26 and Week 50 by treatment group by calculating the point estimate and 2-sided 95% CI, based on exact confidence limits using the Clopper-Pearson method.

A bar chart will also be provided displaying the proportion of participants meeting the criteria for partial remission at Week 26 and Week 50 by treatment group.

5.5. Tertiary/Exploratory Endpoint Analysis

The exploratory analyses will be based on the FAS. These analyses will be descriptive in nature, and no adjustment for multiplicity will be performed. In addition, there will be no imputation for missing data or data collected on or after the receipt of additional standard-of-care therapy (for LN cohort) or treatment discontinuation unless otherwise specified below.

Summary statistics will be computed and displayed by treatment group and by visit, where applicable. Descriptive statistics for continuous variables will minimally include the number of participants, mean, SD, minimum, median, and maximum. For categorical variables, frequencies and percentages will be presented.

The analyses for participants in the LN cohort and participants in the IgAN cohort will be conducted and reported separately. Participants in each disease-specific cohort will be analyzed as randomized, regardless of actual treatment received.

5.5.1. Effect on Hematuria

The effect on hematuria will be summarized by category of RBCs in urine.

The RBCs in urine will be summarized at each time point by treatment group using frequency statistics for categorical variables.

In addition, the percentage of participants with < 10 RBCs will be summarized at each scheduled visit by calculating the point estimate and 2-sided 95% CI, based on exact confidence limits using the Clopper-Pearson method.

5.5.2. SF-36v2

For both cohorts, the SF-36v2 total score, Physical Component Summary (PCS), Mental Component Summary (MCS), and domain scores as defined in Table 4 will be summarized by treatment group at baseline and each postbaseline time point using descriptive statistics for continuous variables for the observed value as well as the change from baseline.

The longitudinal changes in SF-36v2 scores will also be analyzed using the same MMRM method specified in Section 5.4.1. Data collected on or after receipt of additional standard-of-care therapy or treatment discontinuation will be assumed missing and handled in the MMRM as MAR.

Refer to Section 6.3.1 for a more detailed description of the SF-36v2 calculation and scoring methods.

Table 4: SF-36v2 Domain Scores

Scale	Number of Items	Definition of Scale
Physical Functioning (PF)	10	Limitations in physical activity because of health problems
Social Functioning (SF)	2	Limitations in social activities because of physical or emotional problems
Role Limitations Due To Physical Health (RP)	4	Limitations in usual role activities because of physical health problem
Bodily Pain (BP)	2	Prescence of pain and limitations due to pain
General Health (GH)	5	Self-evaluation of personal health
Mental Health (MH)	5	Psychological distress and well-being
Role Limitations Due To Emotional Problems (RE)	3	Limitations in usual role activities because of emotional problems
Vitality (VT)	4	Energy and fatigue

Abbreviations: SF-36v2 = 36-item Short Form Health Survey Version 2

5.5.3. EQ-5D-5L

The EQ-5D-5L is a self-assessed, health-related QoL questionnaire. It is a measure of health status consisting of 2 parts. The first part assesses health in 5 dimensions including mobility, self-care, usual activities, pain/discomfort, and anxiety/depression. Each level is rated on a scale that describes the degree of problems in that area (ie, I have no problems walking about, slight problems walking, moderate problems walking, severe problems walking, or unable to walk). This part of the EQ-5D-5L questionnaire provides a descriptive profile that can be used to generate a health state profile. For example, a participant in health state 12345 would have no problems with mobility, slight problems with self-care (washing or dressing), moderate problems with doing usual activities, severe pain or discomfort and extreme anxiety or depression. The EQ-5D-5L health states can be represented by a single summary number (index value), which reflects how good or bad a health state is according to the preferences of the general population of a country/region. A health state index score will be calculated using a US-specific value set (Pickard, 2019). The second part consists of a visual analog scale (VAS) on which the participant rates their perceived health from 0 (the worst imaginable health) to 100 (the best imaginable health).

For both cohorts, the EQ-5D-5L index score and VAS score will be summarized by treatment group at baseline and each postbaseline time point using descriptive statistics for continuous variables for the observed value as well as the change from baseline.

The longitudinal changes in EQ-5D-5L scores will also be analyzed using the same MMRM method specified in Section 5.4.1. Data collected on or after receipt of additional standard-of-care therapy or treatment discontinuation will be assumed missing and handled in the MMRM as MAR.

Refer to Section 6.3.2 for a more detailed description of the EQ-5D-5L calculation and scoring methods.

5.5.4. Exploratory Biomarkers

Exploratory blood, urine, and kidney biomarker analyses will be documented in a separate biomarker SAP.

5.5.5. Time to CRR and PRR (LN Cohort Only)

For the LN cohort, time to CRR and time to PRR will be summarized using spot urine samples. Participants will be assigned as responders at the time of their CRR or PRR, respectively, or censored at the earliest of their treatment discontinuation time, receipt of additional standard-of-care therapy, study withdrawal or death, or at Week 50 if they have not responded or received additional standard-of-care therapy by then.

Kaplan-Meier cumulative distribution curves will be generated for each treatment group, and a log-rank test comparing the curves will be performed. A corresponding summary table will present the CDF estimate, the number of participants at risk, the number of participants responding, and the number of participants censored at each postbaseline time point by treatment group. The table will also present first quartile, median, and third quartile, along with corresponding 2-sided 95% CI, of time to response.

5.5.6. Overall Renal Response (LN Cohort Only)

For the LN cohort, overall renal response is defined as meeting either CRR or PRR.

Participants who receive additional standard-of-care therapy or who discontinue treatment prior to the endpoint assessment will be considered nonresponders.

The percentage of participants meeting the criteria for overall renal response will be analyzed using a CMH test accounting for the stratification factor at Week 26 and Week 50 and will be summarized by treatment group by calculating the point estimate and 2-sided 95% CI based on exact confidence limits using the Clopper-Pearson method.

5.5.7. Time to UPCR > 50% Decrease (LN Cohort Only)

For the LN cohort, time to UPCR > 50% decrease from baseline will be summarized using spot urine samples. Participants will be assigned as responders at the time of their > 50% decrease from baseline in UPCR or censored at the earliest of their treatment discontinuation time, receipt of additional standard-of-care therapy, study withdrawal or death, or at Week 50 if they have not responded or received additional standard-of-care therapy by then.

Kaplan-Meier cumulative distribution curves will be generated for each treatment group, and a log-rank test comparing the curves will be performed. A corresponding summary table will present the CDF estimate, the number of participants at risk, the number of participants responding, and the number of participants censored at each postbaseline time point by treatment group. The table will also present first quartile, median, and third quartile, along with corresponding 2-sided 95% CI, of time to response.

5.5.8. FACIT-Fatigue (LN Cohort Only)

For the LN cohort, the FACIT-Fatigue score will be summarized by treatment group at baseline and each postbaseline time point using descriptive statistics for continuous variables for the observed value as well as the change from baseline.

The longitudinal changes in FACIT-Fatigue score will also be analyzed using the same MMRM method specified in Section 5.4.1. Data collected on or after receipt of additional standard-of-care therapy or treatment discontinuation will be assumed missing and handled in the MMRM as MAR.

Refer to Section 6.3.3 for a more detailed description of the FACIT-Fatigue calculation and scoring methods.

In addition, the proportion of participants who showed an improvement of at least 3 points for the FACIT-Fatigue scores will be summarized descriptively at each study visit by treatment group by calculating the point estimate and 2-sided 95% CI, based on exact confidence limits using the Clopper-Pearson method.

5.5.9. Anti-dsDNA and Anti-C1q antibodies (LN Cohort Only)

For the LN cohort, anti-dsDNA and anti-C1q antibodies will be summarized at baseline and each postbaseline time point by treatment group using descriptive statistics for the observed value as well as the change from baseline.

In addition, the proportion of participants with anti-dsDNA antibodies below the lower limit of normal (LLN) and with anti-C1q antibodies below the LLN will be summarized descriptively at each study visit by treatment group by calculating the point estimate and 2-sided 95% CI, based on exact confidence limits using the Clopper-Pearson method.

5.5.10. Histology (LN Cohort Only)

For participants in the LN cohort who complete an optional post-treatment biopsy at Week 50, changes in histology will be summarized using the central pathology biopsy results.

Activity index and chronicity index scores will be summarized at baseline and each postbaseline time point by treatment group using descriptive statistics for the observed value as well as the change from baseline.

In addition, shift tables will display the change in LN class, C3 staining, C4d staining, and C1q staining from pretreatment renal biopsy to post-treatment renal biopsy.

5.5.11. Slope of eGFR (IgAN Cohort Only)

For the IgAN cohort, slope of eGFR will be computed using a mixed effect model including data through Week 26 or Week 50, respectively. The model will include eGFR as the response variable, random patient effects for intercepts and slopes, fixed categorical effects of treatment group, randomization stratification factor, and treatment group by visit interaction as well as a fixed, continuous effect of visit (study year).

5.6. Safety Analyses

All safety analyses will be performed on the Safety Set and will be based on the actual treatment received. All safety data will be provided in patient listings by treatment group.

AEs will be coded in MedDRA V 23.0 or higher and presented by MedDRA system organ class (SOC) and preferred term (PT).

No formal hypothesis is planned. Safety data will be presented separately for the Initial Evaluation Period, Extension Period, and Post-treatment Follow-up Period.

5.6.1. Extent of Exposure

Study intervention duration, compliance, and exposure will be summarized overall and by treatment group using the Safety Set.

Treatment duration will be calculated (in weeks) starting from the reference day and summarized using descriptive statistics for continuous variables.

Treatment compliance will be calculated as a percentage based on the number of infusions received out of the number of infusions expected.

Exposure will be calculated as the total number of infusions received and summarized using descriptive statistics for continuous variables as well as using number and percentage of participants in each category.

In addition, the total number of participants with missed infusions and reasons for missed infusions (COVID-19 related and other) will be summarized by treatment group and overall. A by-participant listing of patients with missed infusions due to COVID-19 will also be produced.

5.6.2. Adverse Events

The following definitions will be used for AEs:

- **Pretreatment AE:** Any AE that starts after providing informed consent, but before the first infusion of study intervention (ravulizumab or placebo).
- **Treatment-emergent adverse event (TEAE):** Any AE that starts between the start of the first infusion of study intervention and up to 56 days after the last infusion of study intervention.
- **Treatment-emergent SAE (TESAE):** A TEAE that is serious.
- **Post-treatment AEs:** Any AE that starts 56 days or later after the last infusion of study intervention.

All AEs will be coded using MedDRA V 23 or higher and will be summarized by SOC and PT overall, by severity, and by relationship to treatment. Participants having multiple AEs within a category (eg, overall, SOC, PT) will be counted once in that category. For severity/relationship tables, the participant's highest grade/most related event within a category will be counted.

TEAEs and SAEs will be summarized descriptively by treatment group. Percentages will be based on the number of treated participants in the Safety Set within a treatment group. Tables will be sorted by alphabetic order of SOC and by descending frequency of PT within an SOC.

Pretreatment and post-treatment AEs will be provided in by-participant listings and will not be included in summary tables.

In addition, AEs that occur after the receipt of additional standard-of-care therapy will be provided in a separate listing.

5.6.2.1. Overall Summary of AEs

An overall summary of AEs and SAEs will be presented. The number of events (n) and number of participants with events (n, %) will be shown for the following event subcategories:

- Any TEAE
- TEAEs leading to study treatment discontinuation
- Related TEAEs

These statistics will be prepared separately for all AEs and SAEs. Additionally, the number and percentage of participants who died on study will be presented.

5.6.2.2. AEs and SAEs by SOC and PT

The number of TEAEs and TESAEs and the number and percentage of participants with events will be presented by SOC and PT. Patients are counted once in each SOC and PT. Percentages will be based on the total number of treated patients in the treatment group. SOCs will be listed in descending order of frequency of occurrence.

Additional summary tables stratifying AEs by age, gender, and race will also be provided.

5.6.2.3. AEs and SAEs by SOC, PT, and Relationship

The number of TEAEs and TESAEs and the number and percentage of participants with events will be presented by SOC and PT as described above by relationship (related, not related). If a patient has more than 1 occurrence of an AE, the strongest relationship to study treatment will be used in the summary table. SAEs will be summarized similarly.

5.6.2.4. AEs and SAEs by SOC, PT, and Severity

The number of TEAEs and the number and percentage of participants with events will be presented by SOC and PT as described above by toxicity grade (Grade 1, Grade 2, Grade 3, Grade 4, Grade 5). If a patient has more than 1 occurrence of an AE, the most severe occurrence will be used in the summary table.

5.6.2.5. Deaths and Other Significant AEs

Individual listings will be presented for AEs leading to study treatment discontinuation, AEs leading to withdrawal from the study, AEs starting during administration of study intervention, and fatal AEs.

Adverse events of special interest include meningococcal infections as defined in Section 6.1.8. A summary table and a listing of AEs related to meningococcal infections will be provided.

5.6.3. Additional Safety Assessments

5.6.3.1. Laboratory Parameters

Laboratory assessments are defined in Section 10.2 of Protocol ALXN1210-NEPH-202.

Observed values and changes from baseline in clinical chemistry, hematology, and urinalysis will be summarized descriptively by treatment group at baseline and at each postbaseline time point separately for each disease cohort. For laboratory results that can be classified as normal, low, or high based on normal range values, shifts from baseline in classification will be summarized for all study visits. For analysis purposes, laboratory results based upon standardized units will be used.

Box plots will be presented for the following central lab parameters by visit: alanine aminotransferase, aspartate transaminase, and creatinine.

All data will be presented in listings, and a specific listing of abnormal results will be provided.

5.6.3.2. Vital Signs

Vital signs will include systolic and diastolic blood pressure (mmHg), heart rate (beats per minute), respiratory rate (breaths per minute), temperature (degrees Celsius [°C] or degrees Fahrenheit [°F]), and pulse oximetry (%).

Observed values as well as changes from baseline in body weight and vital signs (blood pressure, heart rate, respiratory rate, and temperature) will be summarized descriptively at each time point by treatment group separately for each disease cohort.

A listing of vital signs will be presented for each disease cohort by treatment group, patient, vital sign, and visit.

5.6.3.3. Electrocardiogram (ECG)

A single 12-lead ECG will be conducted according to the schedule of activities in the protocol to obtain heart rate, PR, QRS, QT and corrected QT (QTc) intervals (QT interval will be corrected for heart rate using Fridericia's formula [QTcF]).

A listing of ECG results will be presented for each disease cohort by treatment group, patient, and visit. Electrocardiograms will be evaluated and summarized as normal, abnormal not clinically significant, or abnormal clinically significant. A shift from baseline to worst on-study ECG table will be presented for ECG results. Observed values and change from baseline in ECG intervals (PR, RR, QT, and QTc) will be summarized descriptively at baseline and each postbaseline time point. The QT interval will be corrected for heart rate using Fridericia's formula (QTcF).

5.6.3.4. Physical Exam

A complete physical examination will include, at a minimum, assessments of the following organs/body systems: skin, head, ears, eyes, nose, throat, neck, lymph nodes, chest, heart, abdomen, extremities, musculoskeletal, and neurological state (with emphasis on presence/degree of edema). An abbreviated physical exam will include, at a minimum, a body-system relevant examination based upon Investigator judgment and participant symptoms.

Adverse changes from baseline in physical examination findings will be classified as AEs and analyzed accordingly.

5.7. Other Analyses

5.7.1. Pharmacokinetic and Pharmacodynamic Analyses

Individual PK/PD data will be collected for all participants. PK analyses will be performed on the PK analysis set and PD analyses will be performed on the PD analysis set.

Serum ravulizumab concentration will be summarized over time with descriptive statistics including the number of observations, arithmetic mean, SD, median, minimum, maximum, coefficient of variation, geometric mean, and geometric coefficient of variation. For the calculation of summary statistics, values below the limit of quantification (BLQ) values will be set to half of the lower limit of quantification (LLOQ) unless a BLQ value falls between 2 measurable values, in which case it will be omitted.

A listing of serum ravulizumab concentration will be presented. BLQ values will be displayed as "< LLOQ."

Graphs of mean serum ravulizumab concentration-time profiles will be constructed. Graphs of serum concentration-time profiles for individual participants may also be provided. Actual dose administration and sampling times will be used for all calculations.

Descriptive statistics will be calculated for all PD endpoints at each sampling time, as appropriate. The PD effects of ravulizumab will be evaluated by assessing the absolute values and changes and percentage changes from baseline in serum concentrations of total C5 and free C5 over time, as appropriate.

5.7.2. Immunogenicity Analyses

All immunogenicity analyses will be performed on the Safety Set.

The incidence of ADAs to ravulizumab will be summarized descriptively separately for each disease cohort by treatment group at baseline and each postbaseline time point. Immunogenicity variables include ADA status categories, ADA response categories, ADA, or neutralizing antibody (NAb) incidence and titer over the duration of the study as follows:

ADA status categories:

- **ADA negative:** An ADA-negative signal in the ADA assay at all timepoints collected for ADA analysis
- **ADA positive:** An ADA-positive signal in the ADA assay at any timepoint collected for ADA analysis

Participants who are ADA positive may be further categorized into ADA response categories as follows:

- **Pre-existing immunoreactivity:** An ADA-positive response with either of the following 2 conditions met:
  - ADA-positive response at baseline with all post-first-dose ADA results negative

    OR
  - ADA-positive response at baseline with all post-first-dose ADA responses less than 4-fold over the baseline titer level
- **Treatment-emergent ADA responses:** An ADA-positive response post-first-dose when baseline results are negative or missing
- **Treatment-boosted ADA responses:** An ADA-positive response post-first-dose that is ≥ 4-fold over the baseline titer level when the baseline result is positive

Treatment-emergent or treatment-boosted ADA responses may be further categorized as follows:

- **Persistent:** ADA responses with 2 or more consecutive ADA-positive samples separated by at least a 16-week period, with no ADA-negative samples in between, irrespective of missing samples
- **Indeterminate:** ADA-positive sample only at the last collected sample
- **Transient:** ADA response that is neither a persistent nor an indeterminate response

ADA-positive samples will be further characterized for neutralizing activity in the NAb assay. NAb status categories are as follows:

- NAb positive
- NAb negative

Associations between ADA response categories and serious and severe adverse events may be explored, including SAEs like systemic hypersensitivity, anaphylaxis, injection/infusion site reactions lasting more than 24 hours, and other immune related SAEs.

Associations between ADA response categories and key efficacy endpoints or variables may be explored to assess the potential impact of immunogenicity on drug efficacy.

All ADA samples will be provided along with titer values in by-participant listings.

5.7.3. COVID-Related Analyses

The following COVID-19 related data will be collected in this study:

- Modified and missed study visits (and COVID-19 related reasons)
- Discontinuations impacted by COVID-19
- COVID-19 exposure
- Missed infusions due to COVID-19

- AEs related to COVID-19
- Protocol deviations related to COVID-19

A listing of all participants affected by these COVID-19 related study disruptions will be provided by participant identifier and investigative site, including a description of the COVID-19 related study disruption.

The number of participants with missed study visits and the reasons for missed study visits (COVID-related or other) will be summarized by treatment groups and overall.

In addition, the number of participants with modified study visits and the reasons for modified study visits (COVID related or other) will be summarized by treatment group and overall. Similarly, the number of participants with modified study visits and the ascertainment method for the modified visit will be summarized by treatment group and overall. A by-participant listing of visit status and assessment ascertainment for participants with modified visits will also be produced.

To assess the impact of change in endpoint ascertainment, descriptive statistics of the primary and key secondary endpoints by method of assessment (in clinic versus alternative method) will be provided using the FAS.

The number of participants with known exposure to COVID-19 will be summarized for pretreatment known exposure and treatment-emergent known exposure by treatment group and overall. A by-participant listing of participants with COVID-19 known exposure or diagnosis will also be produced.

Missed infusions due to COVID-19 will be summarized with overall study intervention exposure as specified in Section 5.6.1.

An overall summary table of TEAEs related to COVID-19 will be presented. In addition, the number of TEAEs related to COVID-19 and the number and percentage of participants with TEAEs related to COVID-19 will be presented by SOC and PT.

Protocol deviations related to COVID-19 will be summarized with the overall protocol deviations as specified in Section 6.2.4 .

5.7.4. Subgroup Analyses

Summaries of the primary efficacy endpoint will be presented by the following subgroups for both cohorts:

- Age at Screening (18 to 65 years, > 65 years)
- Gender (female, male)
- Baseline eGFR (30 to 60, > 60 to 90, > 90 mL/min/1.73 m^2)
- Baseline C3/C4 (at least 1 C3/C4 low, no low C3/C4)

For the LN cohort, summaries of the primary efficacy endpoint will be presented by the following additional subgroups:

- Duration of disease (≤ 1 year, > 1 year)
- Race/ethnicity (African American, non-African American)
- Baseline proteinuria (≤ 3.5 g/g, > 3.5 g/g)
- Corticosteroid induction timing (prior to Screening, during Screening Period)
- LN class based on central pathology (III or III/V, IV or IV/V)
- TMA on renal biopsy based on central pathology (yes, no)
- Disease status (naïve, relapse)
- Baseline renal biopsy activity index based on central pathology (≤ median, > median)
- Baseline renal biopsy chronicity index based on central pathology (≤ median, > median)

For the IgAN cohort, summaries of the primary efficacy endpoint will be presented by the following additional subgroups:

- Duration of disease (≤ 1 year, 1 to 5 years, > 5 years)
- Race (Asian or Other Pacific Islander, non-Asian or Other Pacific Islander)
- Baseline proteinuria (1 to 2 g/day, > 2 g/day)
- Baseline MEST-C score based on central pathology (M0, M1, E0, E1, S0, S1, T0, T1, T2, C0, C1, C2)
- Baseline C3 staining based on central pathology (presence, absence)
- Baseline C4d staining based on central pathology (presence, absence)
- SGLT2 use (yes, no)

For the IgAN cohort, summaries of the primary efficacy endpoint by subgroups of baseline MEST-C score, baseline C3 staining, and baseline C4d staining will be presented separately for participants with biopsies within 1 year of randomization and for participants with biopsies greater than 1 year before randomization, if feasible, based on sample size.

Given that the number of participants in some subgroups may be limited, subgroup categories may be combined as appropriate. Otherwise the subgroup analysis may not be performed if deemed infeasible based on sample size.

Forest plots will be produced showing the results of the primary efficacy endpoint (percentage change in proteinuria from baseline to Week 26) with means and corresponding CIs for each subgroup described above.

5.8. Interim Analyses

5.8.1. Dose Confirmation Analysis

To ensure the adequacy of the dose regimen, an interim PK/PD analysis for dose confirmation will be conducted by an independent clinical pharmacologist. The interim PK confirmation analysis will be conducted using masked PK/PD data from the first 10 participants treated with ravulizumab (a minimum of 3 participants in each disease-specific cohort), using data cut when the tenth participant reaches 2 weeks post-first-dose (ie, at Day 15). The PK dataset for review will include:

- Day 1 maximum concentration (C_{max}), Day 15 trough concentration (C_{trough}), and C_{max} for all 10 participants.
- PK data beyond Day 15 C_{max} timepoint (eg, Day 29 PK) may be included in the dataset (availability depending upon the enrollment rate).
- Total and free C5 data associated with above timepoints and ADA data will be included in the dataset as supportive evidence.

If observed Day 1 C_{max}, Day 15 C_{max} and C_{trough} values, and other available PK/PD data are within the expected range, the study will proceed unchanged. If the totality of the available PK/PD data are not within the expected range, a dose regimen adjustment may be necessary for all participants or for a subset of participants. If all participants require a dose regimen adjustment, enrollment will be paused until a new regimen is determined. If dose regimen adjustment is only required for a subset of participants, enrollment may continue in the subset not requiring dose adjustment. In the event of dose adjustments, the participants treated with the previous dose will switch over to the new dose and continue treatment on study but will be excluded from the primary efficacy analysis. Replacement participants may be enrolled to preserve study power.

5.8.2. Early IgAN Interim Analysis

If appropriate, an early interim analysis may be conducted for the IgAN disease cohort at the discretion of Alexion for the purpose of facilitating Phase 3 study planning and will have no impact on progression of the study. This interim analysis may occur when at least 50% of participants have been randomly assigned to study treatment and have completed the 26-week Initial Evaluation Period. This interim analysis, if performed, will be conducted by a separate unblinded team.

This interim analysis will include all planned efficacy, PK/PD, and safety analyses.

5.8.3. Early LN Interim Analysis

An early interim analysis may be conducted for the LN disease cohort when at least 50% of participants have been randomly assigned to study treatment and have completed the 26-week Initial Evaluation Period. This interim analysis, if performed, will be conducted by a separate unblinded team and will have no impact on the progression of the study.

This interim analysis will include all planned efficacy, PK/PD, and safety analyses.

5.8.4. Week 26 Analysis

A primary efficacy analysis will be performed for each disease-specific cohort at the end of the 26-week Initial Evaluation Period after all participants in the disease-specific cohort have completed or withdrawn from the 26-week Initial Evaluation Period.

This interim analysis will include all planned efficacy and safety analyses and available PK/PD.

5.8.5. Week 50 Analysis

An interim efficacy analysis will be performed for each disease-specific cohort at the end of the 50-week Extension Period after all participants in the disease-specific cohort have completed the Week 50 visit or withdrawn prior to the Week 50 visit.

This interim analysis will include all planned efficacy, PK/PD, and safety analyses.

6. SUPPORTING DOCUMENTATION

6.1. Appendix 1: Technical Specifications for Derived Variables

The following derived data will be calculated prior to analysis.

6.1.1. Disease Duration

Disease duration will be presented as the number of years between date of first infusion and date of diagnosis as reported on the medical history eCRF. Missing dates will be handled as in Section 5.1.4.

6.1.2. Definition of Baseline Values

For the Initial Evaluation Period, baseline is defined as the last available assessment value prior to the first dose of study intervention. For the Extension Period, baseline is defined as the last available assessment prior to the first dose of study intervention administered in the Extension Period.

6.1.3. Change from Baseline

Change from baseline will be calculated as the baseline value subtracted from the value at a particular time point. If 1 of the values is missing and there are no prespecified missing value imputation rules (see Section 5.1.4), then a change from baseline will not be calculated.

6.1.4. Percent Change from Baseline

Percent change from baseline will be calculated as (Change from baseline/Baseline value) x 100. If 1 of the values is missing and there are no prespecified missing value imputation rules (see Section 5.1.4) or if the baseline value is zero, then a percent change from baseline will not be calculated.

6.1.5. Analysis Visits

Summaries over postbaseline time points or analyses at specific postbaseline time points will be performed based on the list of visits described in the schedule of assessment of the protocol. For all assessments, the number of days from baseline will be calculated using the following formula: (date of assessment) - (date of first study treatment) + 1. Relative days prior to Day 1 are calculated as (date of assessment – date of Day 1 visit). This number of days will be used to assign analysis visit.

The analysis visit assignment for a specific assessment will be based on visit windows around each scheduled visit for that specific assessment. The windows for each scheduled visit will go from the midpoint (in days) between the current visit and the previous scheduled visit to the midpoint between the current visit and the subsequent scheduled visit. If the interval separating 2 scheduled visits is an even number of days, that middle day will be included in the later visit window and excluded from the prior visit window. For example, for an assessment with a scheduled visit Day 127, and a prior scheduled visit Day of 113 and subsequent scheduled visit Day of 141, the window will start at 120 days from baseline and will go to 133 days from baseline.

6.1.6. Analysis Value

The values being considered for analysis at a specific postbaseline time point will be based on the analysis visit assigned to that value. If there is more than 1 nonmissing value for a specific assessment with the same analysis visit, the value used for analysis will be the 1 for which the calculated number of days from baseline is closest to the scheduled visit day. If 2 values have the same analysis visit and are the same distance away from the scheduled visit day, the earlier of the 2 values will be used for analysis.

6.1.7. AEs

The analysis of AEs is described in detail in Section 5.6.2.

Treatment-emergent AEs (TEAEs) are events with start dates and start times on or after the date and time of the first dose of study intervention and up to 56 days after the last dose of study intervention. If the start date of an AE is partially or completely missing and the end (stop) date and time of the AE does not indicate that it occurred prior to first dose or 56 days after the last dose, then the determination of treatment-emergent status will be based on the following:

- If the start year is after the year of the first dose of study intervention and before the year of the last dose of study intervention, then the AE is treatment-emergent; else
- If the start year is the same as the year of the first dose of study intervention and
  - the start month is missing, then the AE is treatment-emergent; else if
  - the start month is present and is the same or after the month of the first dose of study intervention and is the same or before the last dose of study intervention, then the AE is treatment-emergent; else,
- If the start date is completely missing, then the AE is treatment-emergent.

All other AEs are considered pretreatment AEs if the start date is prior to the first dose of study intervention or post-treatment AEs if the start date is 56 days or later after the last dose of study intervention.

6.1.8. AEs Related to Meningococcal Infection

To find meningococcal events, the AE dataset will be searched for the following MedDRA PTs:

- Meningitis meningococcal
- Meningococcal bacteraemia
- Meningococcal infection
- Meningococcal sepsis
- Meningococcal carditis
- Encephalitis meningococcal
- Endocarditis meningococcal
- Myocarditis meningococcal

- Optic neuritis meningococcal
- Pericarditis meningococcal

In addition, a medical review will be done to ensure that no relevant events were missed.

6.1.9. CRR (LN Cohort Only)

CRR is defined as meeting all 3 of the following criteria:

- A decrease in mean UPCR to ≤ 0.5 g/g
- eGFR > 60 mL/min/1.73 m^2 or no eGFR reduction ≥ 20% from the baseline value
- No treatment failure

Only central laboratory results from both scheduled and unscheduled visits will be used to assess CRR.

Proteinuria values will meet the criteria for CRR if the mean of two 24-hour UPCR values at the scheduled visit (Week 26 or Week 50) and within 2 weeks prior to the scheduled visit, determined using the actual date of the measurement taken, is ≤ 0.5 g/g.

In the presence of missing central laboratory values, the following rules will be applied:

- If only 1 nonmissing 24-hour urine collection is available at the visit, then this value will be used for analysis.
- If both collections are missing at the visit, then CRR will be treated as missing.

eGFR values will meet the criteria for CRR if the mean of 2 collections at the scheduled visit (Week 26 or Week 50) and within 2 weeks prior to the scheduled visit, determined using the actual date of the measurement taken, is > 60 mL/min/1.73 m^2 or result in a reduction from baseline that is < 20%.

In the presence of missing central laboratory values, the following rules will be applied:

- If only 1 value is available at the visit, then this value will be used for analysis.
- If both values are missing at the visit, then CRR will be treated as missing.

No treatment failure will be determined as the absence of additional standard-of-care therapy received at any time up to Week 26 or Week 50 for protocol-defined renal flare, severe extrarenal SLE flare, or suboptimal response as recorded on the Rescue Therapy eCRF.

6.1.10. Concomitant Medications/Therapies

The analysis of concomitant medications and therapies is described in detail in Section 6.2.2.

Concomitant medications or therapies are defined as any nonstudy medications or therapies that were taken or given while the patient also received study intervention. A medication or therapy will be considered concomitant if the start date is on or after the date of the first dose of study intervention or if the start date is before the first dose of study intervention and the end (stop) date is after the first dose of study intervention. If the start date of a medication/therapy is partially or completely missing and the end (stop) date of the medication/therapy does not indicate that it ended prior to first dose of study intervention, then the determination of the concomitant status will be based on the following:

- If the start year is after the year of the first dose of study intervention, then the medication/therapy is concomitant; else,
- If the start year is the same as the year of the first dose of study intervention and
  - the start month is missing, then the medication/therapy is concomitant; else if
  - the start month is present and is the same or after the month of the first dose of study intervention, then the medication/therapy is concomitant; else,
- If the start date is completely missing, then the medication/therapy is concomitant.

All other medications/therapies are considered prior medications/therapies.

6.1.11. Partial Remission (IgAN cohort only)

Partial remission is defined as meeting the following criteria:

- Proteinuria < 1 g/day

Proteinuria values will meet the criteria for partial remission if the mean of two 24-hour urine collections at the scheduled visit (Week 26 or Week 50) and within 2 weeks prior to the scheduled visit, determined using the actual date of the measurement taken, is < 1 g/24-h (day).

Only central laboratory results from both scheduled and unscheduled visits will be used to assess partial remission.

In the presence of missing central laboratory values, the following rules will be applied:

- If only 1 nonmissing 24-hour urine collection is available at the visit, then this value will be used for analysis.
- If both collections are missing at the visit, then partial remission will be treated as missing.

6.1.12. PRR (LN cohort only)

PRR is defined as meeting all 3 of the following criteria:

- A decrease in UPCR > 50% compared to the baseline value
- eGFR > 60 mL/min/1.73 m^2 or no eGFR reduction ≥ 20% from the baseline value
- No treatment failure

Only central laboratory results from both scheduled and unscheduled visits will be used to assess PRR.

Proteinuria values will meet the criteria for PRR if the mean of two 24-hour UPCR values at the scheduled visit (Week 26 or Week 50) and within 2 weeks prior to the scheduled visit, determined using the actual date of the measurement taken, is > 0.5 g/g and results in a reduction from baseline that is > 50%.

In the presence of missing central laboratory values, the following rules will be applied:

- If only 1 nonmissing 24-hour urine collection is available at the visit, then this value will be used for analysis.
- If both collections are missing at the visit, then PRR will be treated as missing.

eGFR values will meet the criteria for PRR if the mean of 2 collections at the scheduled visit (Week 26 or Week 50) and within 2 weeks prior to the scheduled visit, determined using the actual date of the measurement taken, is > 60 mL/min/1.73 m^2 or results in a reduction from baseline that is < 20%.

In the presence of missing central laboratory values, the following rules will be applied:

- If only 1 value is available at the visit, then this value will be used for analysis.
- If both values are missing at the visit, then PRR will be treated as missing.

No treatment failure will be determined as the absence of additional standard-of-care therapy received at any time up to Week 26 or Week 50 for protocol-defined renal flare, severe extrarenal SLE flare or suboptimal response as recorded on the Rescue Therapy eCRF.

6.1.13. Serum Creatinine and eGFR

Serum creatinine measurements are not reliable with concurrent dialysis. Therefore, all serum creatinine values obtained while a participant is on dialysis will be excluded from all analyses. eGFR will be imputed with a value of 10 (in mL/min/1.73 m^2) while a participant is on dialysis. A participant will be considered on dialysis from the first day of dialysis through 5 days after the end of dialysis. This rule will only be applied to postbaseline assessments of serum creatinine and eGFR.

6.2. Appendix 2: Study and Participant Characteristics

Summaries that are presented as the Table 14.1 or 14.3 series, which are not described in the body of the SAP, are provided here.

6.2.1. Demographics, Disease Characteristics, and History

All demographic and baseline disease characteristics will be summarized using the FAS. Summary statistics will be presented by treatment group and overall. By-participant listings of these data will also be provided.

6.2.1.1. Demographics

The following demographic variables will be summarized:

- Sex
- Race

- Ethnicity
- Age at Screening (years): descriptive statistics (n, mean, median, SD, minimum, maximum) and by frequency of participants in each age category: 18 to 65 years, > 65 years
- Baseline weight (kg): descriptive statistics (n, mean, median, SD, minimum, maximum) and frequency of participants in the following categories: 40 to < 60 kg, 60 to < 100 kg, ≥ 100 kg
- Baseline height (cm)
- Baseline body mass index (kg/m^2)
- Geographical region (North America, Europe, Asia Pacific)

6.2.1.2. Disease Characteristics

The following disease characteristic will be summarized.

- Duration of disease (years): descriptive statistics (n, mean, median, SD, minimum, maximum)
- Baseline eGFR (mL/min/1.73 m^2): descriptive statistics (n, mean, median, SD, minimum, maximum) and frequency of participants in the following categories: 30 to 60, > 60 to 90, > 90
- Baseline C3: descriptive statistics (n, mean, median, SD, minimum, maximum) and frequency of participants with low C3
- Baseline C4: descriptive statistics (n, mean, median, SD, minimum, maximum) and frequency of participants with low C4
- Baseline serum albumin: descriptive statistics (n, mean, median, SD, minimum, maximum)
- Baseline SF-36v2 score: descriptive statistics (n, mean, median, SD, minimum, maximum)
- Baseline EQ-5D-5L: descriptive statistics (n, mean, median, SD, minimum, maximum)

For the LN cohort, the following additional disease characteristics will be summarized:

- Time from eligibility biopsy to randomization (< 1 month, 1 to 3 months, 3 to 6 months, > 6 months)
- LN class based on central pathology (III or III/V, IV or IV/V)
- LN class based on local pathology (III or III/V, IV or IV/V)
- Glomerular C3 staining based on central pathology (0+, 1+, 2+, 3+)
- Glomerular C4d staining based on central pathology (0+, 1+, 2+, 3+)
- Glomerular C1q staining based on central pathology (0+, 1+, 2+, 3+)

- Tubulointerstitial C3 staining based on central pathology (0+, 1+, 2+, 3+)
- Tubulointerstitial C4d staining based on central pathology (0+, 1+, 2+, 3+)
- Tubulointerstitial C1q staining based on central pathology (0+, 1+, 2+, 3+)
- Vascular C3 staining based on central pathology (0+, 1+, 2+, 3+)
- Vascular C4d staining based on central pathology (0+, 1+, 2+, 3+)
- Vascular C1q staining based on central pathology (0+, 1+, 2+, 3+)
- Renal biopsy activity index based on central pathology: descriptive statistics (n, mean, median, SD, minimum, maximum)
- Renal biopsy chronicity index based on central pathology: descriptive statistics (n, mean, median, SD, minimum, maximum)
- TMA on renal biopsy based on central pathology (yes, no)
- Baseline 24-hour UPCR (g/g): descriptive statistics (n, mean, median, SD, minimum, maximum) and frequency of participants in the following categories: ≤ 3.5 g/g, > 3.5 g/g
- Baseline 24-hour urine protein (g/day): descriptive statistics (n, mean, median, SD, minimum, maximum) and frequency of participants in the following categories: 1 to 2 g/day, > 2 g/day
- Baseline spot UPCR (g/g): descriptive statistics (n, mean, median, SD, minimum, maximum) and frequency of participants in the following categories: ≤ 3.5 g/g, > 3.5 g/g
- Baseline disease status (naïve, relapse)
- Corticosteroid induction timing (prior to Screening, during Screening Period)
- Baseline SLEDAI-2K score: descriptive statistics (n, mean, median, SD, minimum, maximum)
- Baseline FACIT-Fatigue score: descriptive statistics (n, mean, median, SD, minimum, maximum)
- Baseline anti-dsDNA autoantibodies: titer (≤ 1:160, ≤ 1:640, ≤ 1:2560)
- Baseline anti-C1q autoantibodies: descriptive statistics (n, mean, median, SD, minimum, maximum) and frequency of participants with antibody levels < LLN

For the IgAN cohort, the following additional disease characteristics will be summarized:

- Baseline 24-hour urine protein (g/day): descriptive statistics (n, mean, median, SD, minimum, maximum) and frequency of participants in the following categories: 1 to 2 g/day, > 2 g/day
- Baseline 24-hour UPCR (g/g): descriptive statistics (n, mean, median, SD, minimum, maximum)

- Time from eligibility biopsy to randomization (< 1 month, 1 to 3 months, 3 to 6 months, 6 to 12 months, 12 to 24 months, > 24 months)
- MEST-C score based on central pathology (M0, M1, E0, E1, S0, S1, T0, T1, T2, C0, C1, C2)
- C3 staining based on central pathology (0+, 1+, 2+, 3+)
- C4d staining based on central pathology (0+, 1+, 2+, 3+)
- C1q staining based on central pathology (0+, 1+, 2+, 3+)

The agreement between local and central pathology diagnosis will be assessed for each disease cohort by interobserver reliability analysis using the kappa statistic reported with 95% CIs. In addition, if central pathology results are limited then local pathology results may be summarized as well in the disease characteristic summary.

6.2.1.3. Medical/Surgical History and Baseline Physical Examination

Medical history will be classified by SOC and PT using the latest available version of standardized MedDRA and will be reported by treatment group and overall for the Safety Set.

Abnormal physical examination results at baseline and postbaseline will be summarized by treatment group for the Safety Set.

By-participant listings of medical/surgical history and abnormal physical examination results will also be produced.

6.2.2. Prior and Concomitant Medications / Therapies

Prior and concomitant medications and procedures will be summarized using the Safety Set.

Prior medications or procedures are defined as medications or procedures taken within 30 days before the start of Screening or during the Screening Period before the first dose of study intervention, as well as any meningococcal vaccine administered within the last 3 years prior to the first dose of study intervention.

Concomitant medications or procedures are defined as medications or procedures received by the participants on or after the first study intervention date (Day 1), including those started before Day 1 and continued after Day 1.

Medications will be coded using the World Health Organization Drug Dictionary (WHO-DRUG) version in use by Alexion at the time of the analysis while nonpharmacologic therapies and procedures (any therapeutic intervention, such as surgery/biopsy or physical therapy) will be coded using MedDRA.

Prior medication and concomitant medication summaries will be presented by WHO-DRUG Anatomical Therapeutic Chemical (ATC) Level 3 and by WHO-DRUG generic name using number [%] of participants by treatment group. Procedures will be summarized similarly but presented by MedDRA Class and PT. In addition, protocol-required vaccinations will be summarized similarly.

By-participant listings of prior and concomitant medications, procedures, and protocol-required vaccinations will also be produced.

6.2.3. Allowed Concomitant Therapy and Additional Standard-of-Care Therapy

Allowed concomitant therapy and additional standard-of-care therapy medications will be summarized using the Safety Set.

Allowed concomitant therapy for participants in the LN cohort consists of standard-of-care treatment for induction and maintenance of LN including corticosteroids and MMF or MMF equivalent. Allowed concomitant therapy for participants in the IgAN consists of standard-of-care treatment including maximally tolerated dose of RAS-blocking agents such as angiotensin-converting enzyme (ACE) inhibitors or angiotensin II receptor blockers (ARBs).

Allowed concomitant therapy use within 3 months prior to Screening and up to the first dose of study intervention will be presented by WHO-DRUG ATC Level 3 and by WHO-DRUG generic name using number [%] of participants by treatment group. In addition, allowed concomitant therapy use at Baseline, including the daily dose at baseline (LN cohort only), will be summarized by treatment group. Changes in allowed concomitant therapy during the study as well as the reason for change will be summarized by treatment group. For the LN cohort, allowed concomitant therapy use at Baseline will include all therapies during the Screening Period and the first study day.

For the LN cohort, participants will receive additional standard-of-care therapy in the event of a protocol-defined renal flare, severe extrarenal SLE flare, or suboptimal response. Additional standard-of-care therapy is defined as intensification of current standard of care or introduction of new immunosuppressive therapies. Additional standard-of-care therapy will be presented by WHO-DRUG ATC Level 3 and by WHO-DRUG generic name by type of flare using number [%] of participants by treatment group.

By-participant listings of allowed concomitant therapy and additional standard-of-care therapy will also be produced.

6.2.4. Protocol Deviations

Protocol deviations will be determined per the standard operating procedure “Identification, Handling, and Documentation of Protocol Deviations” (SOP-G-CL-0044). The number and percent of patients with specific protocol deviations will be summarized for all enrolled participants by important and nonimportant deviations. Protocol deviations will be presented overall as well as separately for those related to COVID-19.

To ensure completeness of the list of protocol deviations, the following will be verified programmatically from the database:

1. Participants from whom informed consent was not obtained
2. Participants who violated any inclusion/exclusion criteria.
3. Participants who received any disallowed medication or therapy
4. Participants randomized to the ravulizumab treatment group who did not receive all of the planned number of doses during the 26-week randomized treatment period.

Protocol deviations from monitoring reports and other relevant sources will also be reviewed, and any important deviations will be included in the list that is summarized and reported.

Summary statistics will be presented by treatment group and overall.

6.3. Appendix 3: Instrument Scoring Details

6.3.1. SF-36v2

The SF-36v2 is a self-administered questionnaire designed to assess generic health-related QoL in healthy and ill adult populations. Eight health domain scores (PF, SF, RP, BP, GH, MH, RE, and VT) and 2 component scores (PCS and MCS) will be calculated.

The SF-36v2 Health Survey with the standard (4-week) recall period will be used in this study. The OPTUM PRO CoRE 1.5 Smart Measurement System will be used to derive the 8 domain scores and 2 component scores. The algorithms used by the software are described below (as excerpted from the User's Guide).

6.3.1.1. Data Cleaning and Item Recoding

First, the data are checked for out-of-range values. Out-of-range values are any values that are outside the range of acceptable item response values for the SF-36v2 Health Survey. Out-of-range values will be converted to missing values. Next, 10 items (BP01, BP02, GH01, GH03, GH05, VT01, VT02, SF01, MH03, MH05) are reverse scored. Reverse scoring of these items is required so that a higher item response value indicates better health for all SF-36v2 Health Survey items and summary measures.

6.3.1.2. Item Recalibration

For most of the SF-36v2 Health Survey items, research to date offers good support for the assumption of a linear relationship between the item scores and the underlying health concept defined by their scales. However, empirical work has shown that 2 items, items GH01 and BP01, require recalibration to satisfy this important scaling assumption. The Bodily Pain (BP) scale requires additional scoring rules because the items offer both different numbers and different content of response choices and administration of item BP02 depended upon the response to an item like item BP01 in past studies.

6.3.1.3. Computation of Raw Scores

After recoding and recalibrating the required item values, a raw score is computed for each scale. This score is the simple algebraic sum of the final values for all items in that scale.

6.3.1.4. Transformation of Raw Scale Score to 0 to 100 Scores

The next step involves transforming each raw scale score to a 0 to 100 scale. This transformation converts the lowest and highest possible scores to zero and 100, respectively. Scores between these values represent the percentage of the total possible score achieved.

6.3.1.5. Transformation of 0 to 100 Scores to T-score Based Scores

The first step in T-score based scoring consists of standardizing each SF-36v2 Health Survey scale using a z-score transformation. A z-score indicates how far a score deviates from the mean in SD units. The z-score for each scale is computed by subtracting the mean 0 to 100 score observed in the 2009 general US population from each SF-36v2 Health Survey scale score (0 to 100) scale and dividing the difference by the corresponding scale SD observed in the 2009 general US population. The means and SD s utilized are dependent upon the recall period option chosen by the user, based on the SF-36v2 Health Survey form used to collect the data being scored.

The next step of the T-score based scoring is to linearly transform each SF-36v2 Health Survey z-score to have a mean score of 50 and a SD of 10. This is done by multiplying each SF-36v2 Health Survey z-score by 10 and adding the resulting product to 50. These are referred to as ‘norm-based’ scores. The norm-based scores will be used for the 8 domain scores.

6.3.1.6. Scoring the SF-36v2 Health Survey Component Summary Measures

The first step in scoring the component summary measures consists of standardizing each SF-36v2 Health Survey scale using a z-score transformation as described previously. The z-score for each scale is computed by subtracting the mean 0 to 100 score observed in the 2009 general US population from each SF-36v2 Health Survey scale score (0 to 100) scale and dividing the difference by the corresponding scale SD observed in the 2009 general US population. The means and SD utilized are dependent upon the recall period option chosen by the user, based on the SF-36v2 Health Survey form used to collect the data being scored.

After a z-score has been computed for each SF-36v2 Health Survey scale, the second step involves computation of aggregate scores for the physical and mental summaries using weights (factor score coefficients) derived from the 1990 general US population. These are the same weights as those used to score PCS and MCS from the SF-36 Health Survey. An aggregate physical score is computed by multiplying the z-score of each SF-36v2 Health Survey scale by its associated physical factor score coefficient and summing the 8 products. If any of the scale scores are missing, then the aggregate physical score is not computed. An aggregate mental score is computed by multiplying the z-score of each SF-36v2 Health Survey scale by its associated mental factor score coefficient and summing the 8 products. If any of the scale scores are missing, then the aggregate mental score is not computed.

The third step involves transforming the aggregate physical and mental summary scores to the T-score Based (50, 10) scoring. This is done by multiplying each aggregate summary score obtained from Step 2 by 10 and adding the resulting product to 50.

6.3.1.7. Handling of Missing Items

The maximum data recovery option will be used for missing data estimation. This results in the application of algorithms that compute a scale score for those respondents whom have answered at least 1 item that represents that construct. For the PF scale, item parameters obtained through item response theory methods are used to estimate a missing value on an item based upon a respondent's responses to answered items. For the 7 remaining scales, a person-specific estimate based on the mean response to the answered items on the scale is used to estimate a missing value. Additionally, a PCS and MCS score is calculated for those respondents whom have calculated scores on at least 7 of the 8 SF-36v2 Health Survey scales. However, PCS is not estimated if the PF scale is missing, and MCS is not estimated if the MH scale is missing.

6.3.2. EQ-5D-5L

The EQ-5D-5L version will be used in this study. EQ-5D health states, defined by the EQ-5D descriptive system, may be converted into a single summary index by applying a formula that attaches values (also called weights) to each of the levels in each dimension. The index can be calculated by deducting the appropriate weights from 1, the value for full health (ie, state 11111). The collection of index values (weights) for all possible EQ-5D health states is called a value set. EQ-5D-5L index scores for this study will be obtained using the composite time trade-off (cTTO) method based on the Tobit model (Pickard, 2019). The calculation is illustrated below.

**US cTTO**		**Example: the value for health state 21354**
Full health (11111)		Full Health = 1
Mobility level 2	-0.096	-0.096
Mobility level 3	-0.122	
Mobility level 4	-0.237	
Mobility level 5	-0.322	
Self-Care level 2	-0.089	0
Self-Care level 3	-0.107	
Self-Care level 4	-0.220	
Self-Care level 5	-0.261	
Usual Activity level 2	-0.068	
Usual Activity level 3	-0.101	-0.101
Usual Activity level 4	-0.255	
Usual Activity level 5	-0.255	
Pain/Discomfort level 2	-0.060	
Pain/Discomfort level 3	-0.098	
Pain/Discomfort level 4	-0.318	
Pain/Discomfort level 5	-0.414	-0.414
Anxiety/Depression level 2	-0.057	
Anxiety/Depression level 3	-0.123	
Anxiety/Depression level 4	-0.299	-0.299
Anxiety/Depression level 5	-0.321	

US cTTO	Example: the value for health state 21354
Health State Index Score	= 1 - 0.096 - 0 - 0.101 - 0.414 - 0.299 = 0.090

If data for some but not all dimensions are missing at a specific time point, data for the missing dimension(s) from the last available assessment prior to the time point with missing data will be used in the US cTTO calculation. If all dimensions are missing, the last available US cTTO score prior to the time point with missing data will be used.

The EQ VAS records the respondent's self-rated health on a vertical VAS where the endpoints are labeled 'The best health you can imagine' for 100 and 'The worst health you can imagine' for 0. Missing values will be coded as '999.' If there is a discrepancy between where the respondent has placed the X and the number written in the box, number in the box will be used for VAS.

6.3.3. FACIT-Fatigue

The FACIT-Fatigue questionnaire consists of 13 items scored on a 5-point Likert scale (0 = not at all, 4 = very much). The FACIT-Fatigue subscale scoring guideline (V 4) will be used as follows (https://www.facit.org/measures/FACIT-Fatigue):

- All negatively stated items (ie, all items except An5 and An7 from the eCRF) are to be reversed by subtracting the response from 4.
- After reversing the proper items, all items are summed to obtain a score.
- The Fatigue subscale score is then calculated by multiplying the sum of the item scores by 13, then dividing by the number of items answered.

When there are missing data, prorating by subscale in this way is acceptable as long as more than 50% of the items were answered. The score has a range of 0 to 52 with higher scores indicating better QoL.

6.4. Appendix 4: Additional Details on Statistical Methods

6.4.1. SAS Code for ANCOVA Analysis

The primary analysis of the primary endpoint utilizes ANCOVA analysis for evaluating the percentage change from baseline to Week 26 in proteinuria. Sample code is provided below:

Step 1: Impute data on or after receipt of additional standard-of-care therapy (LN cohort only) using the value collected at the time of renal flare or extrarenal SLE flare

Step 2: Impute data on or after treatment discontinuation using multiple imputation as described in Section 6.4.3.

Step 3: Analyze imputed datasets using ANCOVA analysis

```
proc glm data=ADEFF;
    by _imputation_;
    class trt01pn strat;
    model chg= trt01pn base strat/ solution;
    lsmeans trt01pn / cl diff stderr;
run;
```

where trt01pn is the randomized treatment group, base is the log(proteinuria) value at baseline, strat is the randomization stratification variable and chg is the change from baseline in log(proteinuria).

Step 4: Inferences from each complete data set will be combined to obtain an overall test statistic for each treatment effect.

```
proc mianalyze data=diff2;
    by avisitn;
    modeleffects estimate;
    stderr;
run;
```

6.4.2. SAS Code for MMRM Analysis

The secondary endpoint of percentage change from baseline to Week 50 in proteinuria utilizes MMRM analysis. Other secondary endpoints assessing longitudinal changes will also utilize MMRM analysis. Sample code is provided below:

Step 1: Impute data on or after receipt of additional standard-of-care therapy (LN cohort only) using the value collected at the time of renal flare, extrarenal SLE flare, or suboptimal response, if applicable

Step 2: Analyze imputed dataset using MMRM analysis

```
proc mixed data=ADEFF method=reml;
    class subjid trt01pn avisitn strat;
    model chg= trt01pn avisitn trt01pn*avisitn base strat /ddfm=kr solution;
    repeated avisitn/type=un subject=subjid;
    lsmeans trt01pn *avisitn/cl diff;
run;
```

where subjid is the patient identifier variable, trt01pn is the randomized treatment group, avisitn is the visit variable, base is the log(proteinuria) value at baseline, strat is the randomization stratification variable and chg is the change from baseline in log(proteinuria).

6.4.3. SAS Code for Multiple Imputation

For the primary analysis of the primary endpoint, responses after treatment discontinuation will be imputed using a multiple imputation approach assuming the data are MAR and using a regression model. Sample code is provided below:

Step 1: Imputation using a monotone regression model at Week X

```
proc mi data=ADEFF out=outmi seed=123 nimpute=1000;
    class trt01pn strat;
    var trt01pn base strat Week26;
    monotone reg(/details);
run;
```

where trt01pn is the randomized treatment group, base is the log(proteinuria) value at baseline, strat is the randomization stratification variable, and Week 26 is the change from baseline in log(proteinuria) at Week 26.

6.4.4. SAS Code for Placebo-based Imputation

A sensitivity analysis for the primary endpoint will be performed using placebo-based imputation for responses after treatment discontinuation. In this analysis, data occurring after treatment discontinuation will be set to missing and multiple imputation will then be performed, using a regression method obtained only from placebo-treated participants with terms for baseline value and the randomization stratification variable.

The following is a partial SAS code for the placebo-based pattern imputation at Week 26:

```
proc mi data=ADEFF out=outmi seed=123 nimpute=1000;
    class trt01pn strat;
    var base strat Week26;
    monotone reg(/details);
    mnar model (Week26/modelobs=(trt01pn='Placebo'));
run;
```

where trt01pn is the randomized treatment group, base is the log(proteinuria) value at baseline, strat is the randomization stratification variable, and Week26 is the change from baseline in log(proteinuria) at Week 26.

The 1000 imputed data sets are then analyzed using ANCOVA (refer to Section 6.4.1 for the SAS code), and the PROC MIANALYZE procedure will be used to generate valid statistical inferences about these parameters.

6.4.5. SAS Code for Tipping Point Sensitivity Analysis

A sensitivity analysis for the primary endpoint will be performed using the tipping point approach where a search is conducted for a tipping point that reverses the study conclusion from being favorable to ravulizumab to being unfavorable. For the tipping point sensitivity analysis, the missing data mechanism for the missing change from baseline in log proteinuria values at Week 26 will be considered to be missing-not-at-random (MNAR). Imputations are performed for missing change observations for the ravulizumab treated patients assuming not the full treatment effect, but the treatment effect adjusted by a shift parameter delta. After obtaining complete data sets for multiple shift parameters, the complete data sets will be used in the MMRM analysis, and inferences from each complete dataset will be combined using SAS PROC MIANALYZE to obtain an overall test statistic for each shift value. Multiple shift parameters will be tested until the inference concludes that statistical significance disappears.

The following is a partial SAS code for the multiple imputation analysis for a specified shift parameter at Week 26:

```
proc mi data=ADEFF out=outmi seed=123 nimpute=1000;
    class trt01pn strat;
    monotone method=reg ;
    var trt01pn strat base Week26;
    mnar adjust (Week 26 /shift=delta adjustobs=(trt01pn='Ravulizumab'));
```

where trt01pn is the randomized treatment group, base is the log(proteinuria) value at baseline, Week26 is the change from baseline in log(proteinuria), and strat is the randomization stratification.

The 1000 imputed data sets are then analyzed using ANCOVA (refer to Section 6.4.1 for the SAS code), and the PROC MIANALYZE procedure will be used to generate valid statistical inferences about these parameters.

6.5. Appendix 5: Changes to Protocol-planned Analyses

Based on actual data received from the central laboratory, the RBCs in urine is categorical data. Hence, the endpoint of hematuria will be analyzed as a categorical variable instead of a continuous variable.

6.6. Appendix 6: List of Abbreviations

Table 5 shows the abbreviations and acronyms that are used in this SAP.

Table 5: Abbreviations and Acronyms

Abbreviation or acronym	Explanation
ACE	Angiotensin-converting enzyme inhibitors
ADA	antidrug antibody
AE	adverse event
ANCOVA	analysis of covariance
anti-dsDNA	anti-double-stranded DNA
ARB	angiotensin II receptor blockers
ATC	Anatomical Therapeutic Chemical
BLQ	below the limit of quantification
CDF	cumulative distribution function
CI	confidence interval
C_{max}	maximum concentration
CMH	Cochran–Mantel–Haenszel
CRR	complete renal response
Ctrough	trough concentration
ECG	electrocardiogram
eCRF	electronic case report form
eGFR	estimated glomerular filtration rate
EQ-5D-5L	5-level EuroQol-5 Dimension
FACIT-Fatigue	Functional Assessment of Chronic Illness Therapy – Fatigue
FAS	Full Analysis Set
GMR	geometric mean ratio
IC	inclusion criteria
IgAN	immunoglobulin A nephropathy
IXRS	Interactive Voice/Web Response System
LLN	Lower limit of normal
LLOQ	lower limit of quantification
LN	lupus nephritis
MedDRA	Medical Dictionary for Regulatory Activities
MAR	missing-at-random
MSC	Mental Component Summary
mFAS	Modified Full Analysis Set
MMF	mycophenolate mofetil
MMRM	mixed effect model for repeated measures
MNAR	missing-not-at-random
NAb	neutralizing antibody
PD	pharmacodynamic(s)
PK	pharmacokinetic(s)
PPS	Per-protocol Set
PRR	partial renal response
PSC	Physical Component Summary
PT	preferred term (MedDRA)
QoL	quality of life
QTc	corrected QT
QTcF	QT interval corrected using Fridericia's formula
RAS	renin-angiotensin system

Table 5: Abbreviations and Acronyms

Abbreviation or acronym	Explanation
RBC	red blood cell
SAE	serious adverse event
SAS®	Statistical Analysis Software®
SAP	Statistical Analysis Plan
SD	standard deviation
SF-36v2	36-item Short Form Health Survey Version 2
SLE	systemic lupus erythematosus
SLEDAI-2K SELENA Modification	Systemic Lupus Erythematosus Disease Activity Index Safety of Estrogen in Lupus Erythematosus National Assessment Modification
SOC	system organ class (MedDRA)
TEAE	treatment-emergent adverse event
TESAE	treatment-emergent serious adverse event
UPCR	urine protein-to-creatinine ratio
VAS	visual analog scale
WHO-DRUG	World Health Organization Drug Dictionary

7. REFERENCES

Appel GB, Contreras G, Dooley MA, et al. Mycophenolate mofetil versus cyclophosphamide for induction treatment of lupus nephritis. J Am Soc Nephrol. 2009;20(5):110-112.

Fellstrom BC, Barratt J, Cook H, et al. Targeted-release budesonide versus placebo in patients with IgA nephropathy (NEFIGAN): a double-blind, randomised, placebo-controlled phase 2b trial. Lancet. 2017;289(10084):2117-2127.

Ginzler EM, Dooley MA, Aranow C, et al. Mycophenolate mofetil or intravenous cyclophosphamide for lupus nephritis. N Engl J Med. 2005;353(21):2219-2228.

Levey AS, Gansevoort RT, Coresh J, et al. Change in albuminuria and GFR as endpoints for clinical trials in early stages of CKD: a scientific workshop sponsored by the National Kidney Foundation in collaboration with the US Food and Drug Administration and European Medicines Agency. Am J Kidney Dis. 2020;75(1):84-104.

Pickard AS, et al. United States Valuation of EQ-5D-5L Health States Using an International Protocol. Value Health. 2019;22(8):931-941.

Ratitch B, O'Kelly M, Tosiello R. Missing data in clinical trials: from clinical assumptions to statistical analysis using pattern mixture models. Pharm Stat. 2013;12(6):337-347.

Ratitch B, O'Kelly M. Clinical Trials with Missing Data: A Guide for Practitioners. Chichester: John Wiley & Sons, 2014.

ALXN1210-NEPH-202 SAP v3.0_31Jul2024

Final Audit Report 2024-08-13

Created:	2024-08-13 (Greenwich Mean Time)
By:	PPD (PPD)
Status:	Signed
Transaction ID:	PPD

"ALXN1210-NEPH-202 SAP v3.0_31Jul2024" History

Document created by PPD (PPD)
2024-08-13 - 1:56:58 AM GMT

Document emailed to PPD (PPD) for signature
2024-08-13 - 1:57:58 AM GMT

Document emailed to PPD (PPD) for signature
2024-08-13 - 1:57:58 AM GMT

Document emailed to PPD (PPD) for signature
2024-08-13 - 1:57:58 AM GMT

PPD (PPD) authenticated with Adobe Acrobat Sign.
Challenge: The user opened the agreement.
2024-08-13 - 1:58:31 AM GMT

PPD (PPD) authenticated with Adobe Acrobat Sign.
Challenge: The user completed the signing ceremony.
2024-08-13 - 1:59:11 AM GMT

Document e-signed by PPD (PPD)
Signing reason: I approve this document
Signature Date: 2024-08-13 - 1:59:12 AM GMT - Time Source: server

Email viewed by PPD (PPD)
2024-08-13 - 1:16:15 PM GMT

PPD (PPD) authenticated with Adobe Acrobat Sign.
Challenge: The user opened the agreement.
2024-08-13 - 1:17:14 PM GMT

PPD (PPD) authenticated with Adobe Acrobat Sign.

Challenge: The user completed the signing ceremony.

2024-08-13 - 1:18:44 PM GMT

Document e-signed by PPD (PPD)

Signing reason: I approve this document

Signature Date: 2024-08-13 - 1:18:45 PM GMT - Time Source: server

PPD (PPD) authenticated with Adobe Acrobat Sign.

Challenge: The user opened the agreement.

2024-08-13 - 2:08:28 PM GMT

PPD (PPD) authenticated with Adobe Acrobat Sign.

Challenge: The user completed the signing ceremony.

2024-08-13 - 2:10:12 PM GMT

Document e-signed by PPD (PPD)

Signing reason: I approve this document

Signature Date: 2024-08-13 - 2:10:13 PM GMT - Time Source: server

Agreement completed.

2024-08-13 - 2:10:13 PM GMT
